# Make it Double: [4]Cumulene Thermal Core Transformation and Chemical Reduction

**DOI:** 10.1021/acs.orglett.5c03034

**Published:** 2025-09-23

**Authors:** Zheng Zhou, Zheng Wei, Matthew A. Johnson, Yikun Zhu, Yu Qiu, Rik R. Tykwinski, Marina A. Petrukhina

**Affiliations:** † Department of Chemistry, 1084 University at Albany, State University of New York, Albany, New York 12222, United States; ‡ School of Materials Science and Engineering, Tongji University, Shanghai 201804, China; § Department of Chemistry, 3158 University of Alberta, Edmonton, Alberta T6G 2G2, Canada

## Abstract

Heating of [4]­cumulene **1** at 235 °C in vacuo affords the head-to-head [4]­radialene dimer **2**, which subsequently sublimes to produce single crystals. Reduction of **2** with Cs produced a ring-opened cumulene dimer dianion (**2**_**TR**_^2–^) crystallized as [Cs^+^(18-crown-6)_2_]­[Cs^+^(**2**_**TR**_^2–^)]·C_6_H_14_ (**3**). X-ray diffraction analysis revealed an unusual core transformation during the 2-fold reduction, with increased bond length alternation resembling an alkyne. Moreover, oxidation of **2**_**TR**_^2–^ regenerated **1**, demonstrating reversible cumulene–radialene interconversion.

## Graphical abstract

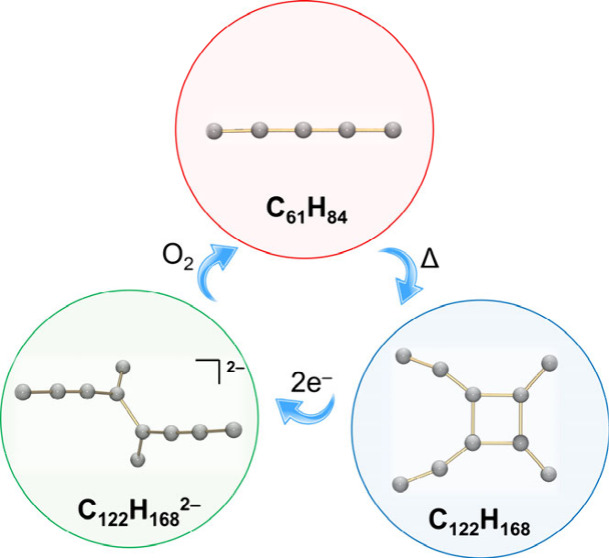

[*n*]­Cumulenes are a class of linear carbon-rich molecules with consecutive C=C double bonds, composed entirely of sp-hybridized carbon atoms.^1–5^ Their unique π-conjugation imparts intriguing physical and chemical properties, making them useful as reactive intermediates, molecular wires, and components of conjugated materials.^6–10^ A different number of double bonds leads to the formation of “odd” or “even” cumulenes (Scheme 1a). Odd cumulenes, with coplanar end groups, are more synthetically accessible and thus better studied.^11,12^ Even-numbered cumulenes (*n* ≥ 4) feature orthogonal end groups, which lead to higher reactivity but poor stability (only [4]­cumulenes have been reported to date).^10,13,14^

**Scheme 1:**
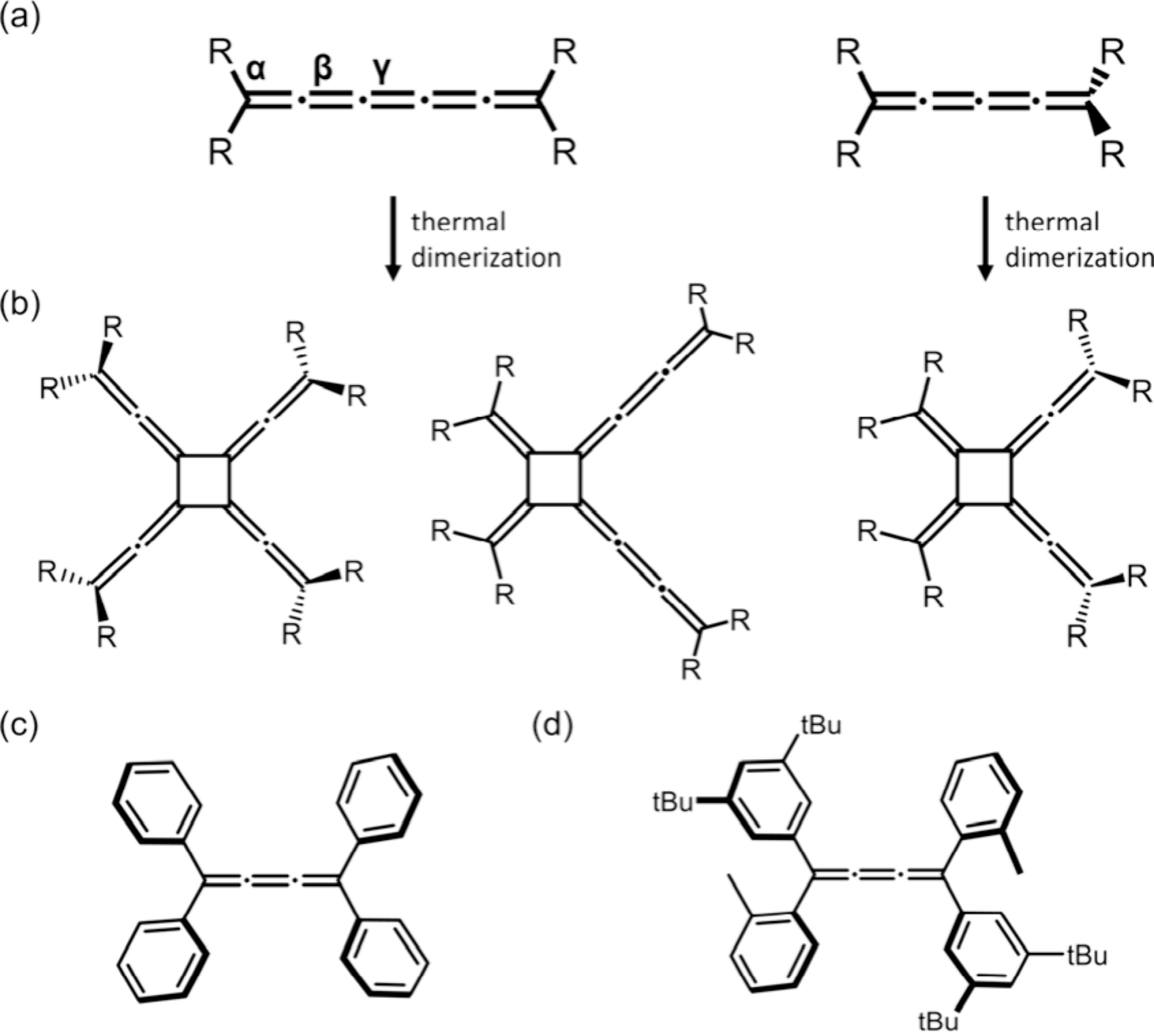
(a) Depictions of an Odd [5]­Cumulene (Left) and an Even [4]­Cumulene (Right), (b) Thermal Dimerization Reactions to Afford [4]­Radialenes, and Structures of (c) [3]­Ph and (d) [3]­TrTol

[*n*]­Cumulenes can undergo dimerization via thermal or photochemical cyclization, typically leading to a radialene framework. The reactivity reported in previous studies can often be correlated to steric or electronic effects from terminal groups.^8,11,15^ For example, [5]­cumulenes with aryl or alkyl substituents preferentially dimerize at the β- or γ-position, forming symmetric or asymmetric [4]­radialenes (Scheme 1b).^16,17^ With small groups such as Me, α-dimerization becomes dominant.^18,19^ Expanded radialenes are accessible from higher cumulenes,^15^ but [4]­cumulenes remain rarely studied, with only three dimeric [4]­radialenes reported to date.^20–23^ While the forward cyclization of cumulenes is established, the reverse transformation of radialenes back to cumulenes remains unknown.

Chemical reduction offers a powerful tool to probe structural and electronic features of π-systems. Studies of bowl-shaped molecules, helicenes, nanohoops, and contorted nanographenes have revealed that electron addition can induce topology-driven transformations and supramolecular reorganizations.^24–35^ Moreover, reduction can promote bond activation or rearrangement, especially in strained π-systems, making it a valuable strategy for synthesizing novel carbon-rich materials.^36–40^

Despite the unsaturated, π-rich framework of cumulenes and radialenes, their redox chemistry is poorly understood. Previous chemical reduction studies are largely limited to [3]­cumulenes.^41–45^ For instance, Bock and Kemula reported that 2-fold reduction of [3]­Ph (Scheme 1c) leads to a twisted cumulene backbone with enhanced bond length alternation (BLA).^41,42^ In contrast, we previously showed that a substituted [3]­cumulene ([3]­TrTol, Scheme 1d) remains linear upon reduction to a dianion, though BLA increases progressively.^46^ In that system, both solvent-separated and contact ion pairs were isolated, and metal binding further enhanced BLA. We recently reported the first reduction of an even [4]­cumulene ([4]­DtBP, **1**, Scheme 2 bottom), isolating both mono- and doubly reduced products.^47^ The cumulenic core bends upon reduction, and its end groups twist from orthogonal to near-planar, demonstrating unique structural adaptability.

**Scheme 2:**
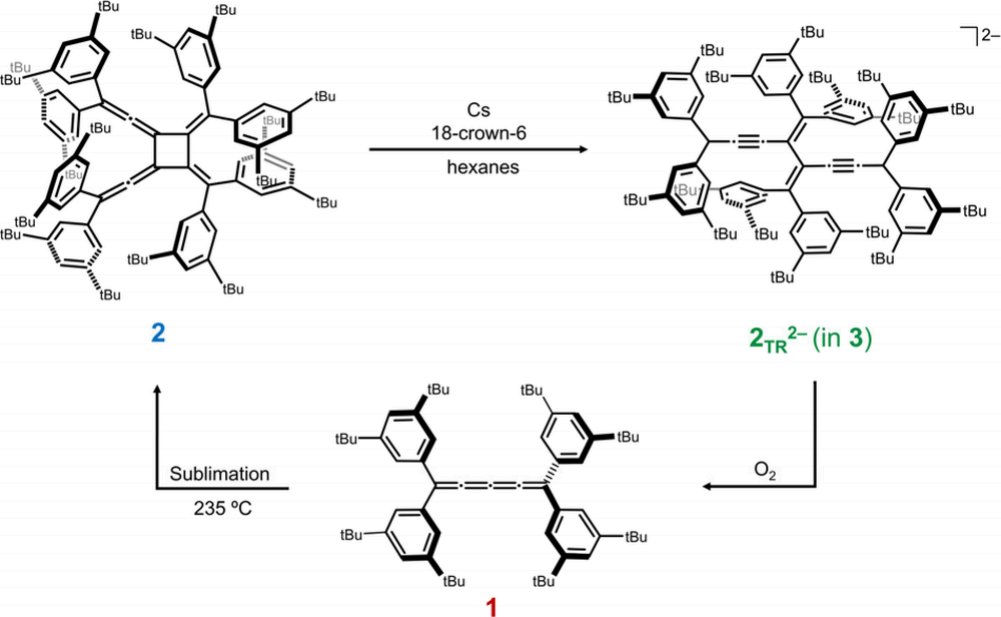
Thermal Dimerization of **1** and Sublimation to Afford **2**, Reduction of **2** with Cs Metal in Hexane to Give **2**_**TR**_^**2–**^, and Oxidation to Regenerate **1**

In this work, we discovered that [4]­DtBP readily forms a radialene upon heating, followed by sublimation to produce single crystals. As no chemical reduction studies of such π-systems have been reported, we were interested to reveal: (i) How does an unsymmetric [4]­radialene respond to an electron addition? (ii) How many electrons can it readily accept? (iii) Does the reduction lead to new chemistry? To address these questions, we report the first reactivity study of a radialene (**2**) using chemical reduction methods. The resulting products with two heavy alkali metals, Cs and Rb, were isolated and fully characterized by X-ray crystallography combined with UV–vis and NMR spectroscopies. This analysis revealed unique reduction-induced core transformation of a radialene **2** to form a new dimerized dianion, **2**_**TR**_^2–^ (TR stands for transformation). Remarkably, the latter can be converted back to the parent cumulene upon oxidation.

[4]­Cumulene **1** was heated in vacuo at 235 °C, which initiated the solid-state dimerization to afford **2** (see SI for details). At this temperature, **2** sublimes to yield orange block-shaped crystals of **2** in 90% yield (Scheme 2). Phase purity was confirmed by ^1^H NMR (Figures S5–S6 and S20) and powder XRD (Figure S25 and Table S2). This solvent- and catalyst-free sublimation is significantly more efficient than the reported solvent-mediated method (<5% yield in toluene over days).^23^ X-ray diffraction analysis confirmed **2** as a new nonsolvated polymorph (C_122_H_168_) crystallizing in the orthorhombic *P*2_1_2_1_2_1_ space group, in contrast to the triclinic *P*–1 structure of the solvated form.^23^

To explore the unreported redox behavior of [4]­radialenes, we investigated the chemical reduction of **2** using Cs metal in hexane at room temperature (Scheme 2). The solution rapidly turned dark green (monoanion) and then red brown, indicating formation of a doubly reduced species (Figures S1–S2). After treatment with 18-crown-6, dark-brown crystals of **3** ([Cs^+^(18-crown-6)_2_]­[Cs^+^(**2**_**TR**_^2–^)]·C_6_H_14_) were obtained. X-ray diffraction analysis confirmed a ring-open structure. Remarkably, oxidation of **3** with O_2_ regenerated neutral cumulene **1** (Figures S22–S23), demonstrating a reversible transformation between cumulene and radialene frameworks.

Compounds **1**–**3** were first analyzed by UV–vis and NMR spectroscopy. As shown in the UV–vis spectra (Figure 1a), **1** displays a λ_max_ absorption at 440 nm (red), which shifts slightly to 460 nm in dimerized **2** (blue). In contrast, the doubly reduced product **3** exhibits a pronounced bathochromic shift with λ_max_ at 726 nm and additional bands at 459 and 542 nm, indicating significant electronic changes upon two-electron reduction. In the ^1^H NMR spectra (Figure 1b), the symmetric aryl signals of **1** become split in **2** due to the asymmetry of the radialene core (6.12–7.99 ppm), a pattern that is further resolved at −80 °C. Similar splitting is observed in **3**, with slight downfield shifts attributed to electron addition. The ^133^Cs NMR spectrum of **3** confirms the presence of both the solvent-separated Cs^+^ (1.7 ppm) and coordinated Cs^+^ (−44.2 ppm) ions.

**Figure 1:**
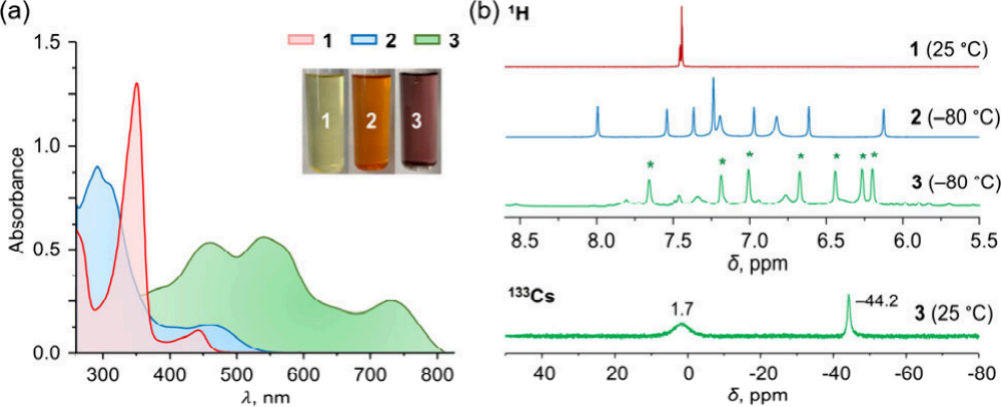
(a) UV–vis absorption spectra of **1**–**3** in THF at 25 °C. (b) ^1^H NMR spectra of **1**–**3** in aromatic regions (* indicates signals of compound **3** in the spectrum) and ^133^Cs NMR spectrum of **3** in THF-*d*_8_.

X-ray crystallographic study of [4]­radialene **2** (Figure 2a) reveals dimerization of **1** at the C3 and C4 positions, forming new C–C bonds (C3–C8:1.519(5) Å; C4–C9:1.499(5) Å, Figure 2b). The elongation of C3–C4 and C8–C9 bonds (1.274(3) to 1.493(5) Å) indicates conversion from double bonds of **1** to single bonds of **2** (Table 1). Compared to related alkyl-substituted radialenes,^21^ these bonds are shorter. Notably, **2** crystallizes in a chiral orthorhombic space group, distinct from the solvated polymorph.^23^ Significant distortion is observed in the allene moieties of **2**, with an average deviation from linearity of 18.4°, far greater than in the previously reported [4]­radialenes (3–7°).^8,15,21,48,49^ This is likely due to intramolecular π···π and C–H···π interactions between bulky substituents (Figure S46). Vinylidene angles range over 131.1–136.9°, which is consistent with literature values.^8,15,21,49,50^ The four aryl groups adopt torsion angles of 47.7–81.9°, twisted out of conjugation.

**Figure 2:**
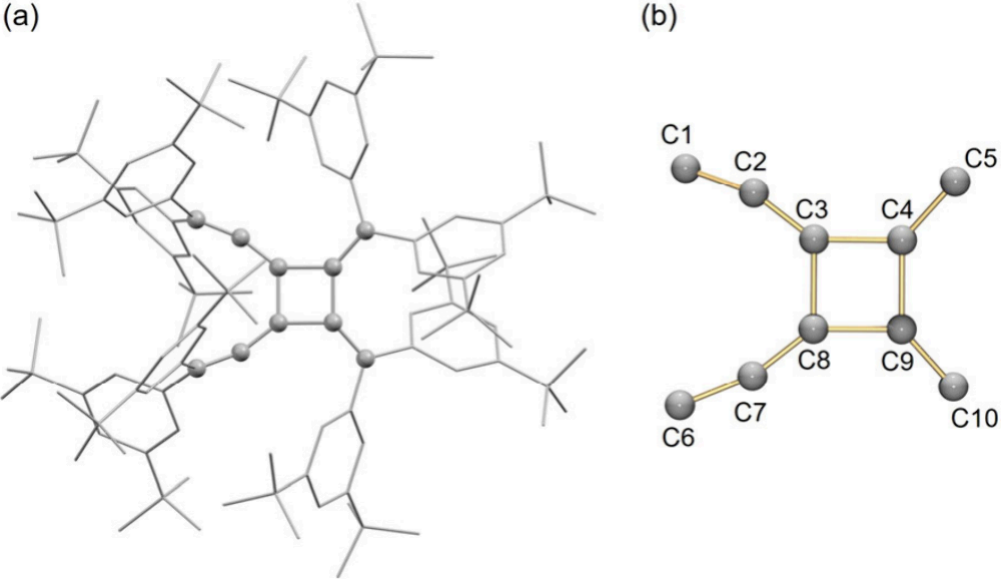
(a) Crystal structure of **2** in a mixed model and (b) its central carbon core in a ball-and-stick model. H atoms and all substituents are omitted for clarity.

**Table 1:**
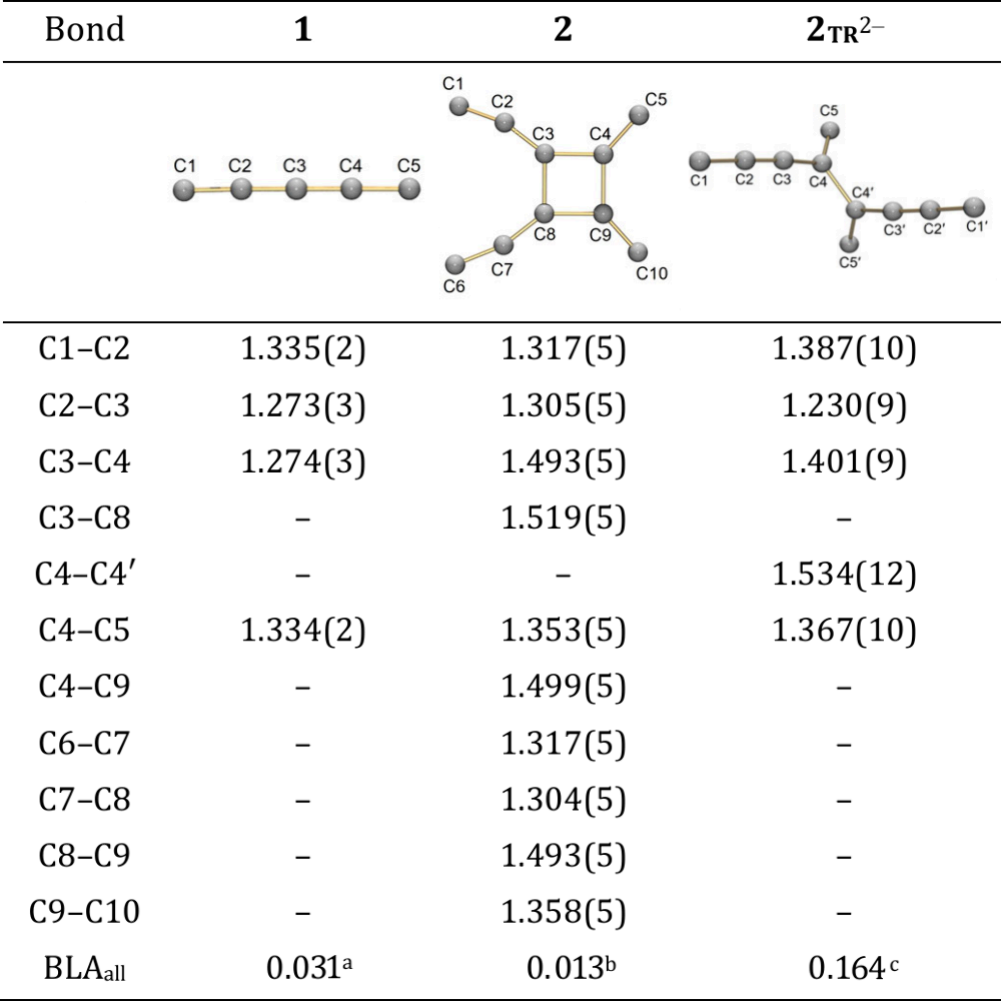
Key Bond Distances (Å) in **1**, **2**, and **2**_**TR**_^2–^, along with the Labeling Schemes

a BLA is calculated as (C2–C3) – (C3–C4).

b BLA is calculated as [(C1–C2) – (C2–C3)]/2 + [(C6–C7) – (C7–C8)]/2.

c BLA is calculated as [(C1–C2) + (C3–C4)]/2 – (C2–C3).

Reduction of **2** with Cs yields the new dianion **2**_**TR**_^2–^, crystallized as **3** (Figure 3a). The product comprises a contact-ion pair of [Cs^+^(C_122_H_168_^2–^)]^−^ with a solvent-separated cation, [Cs^+^(18-crown-6)_2_]^+^. The bound Cs1 ion interacts with both cumulene and aryl carbon sites (Cs···C: 3.143(9)–3.791(10) Å), while Cs2 is fully encapsulated by two crown ethers (Cs···O: 3.189(11)–3.358(14) Å). These two distinct Cs^+^ environments match the ^133^Cs NMR data (Figure 1b).

**Figure 3:**
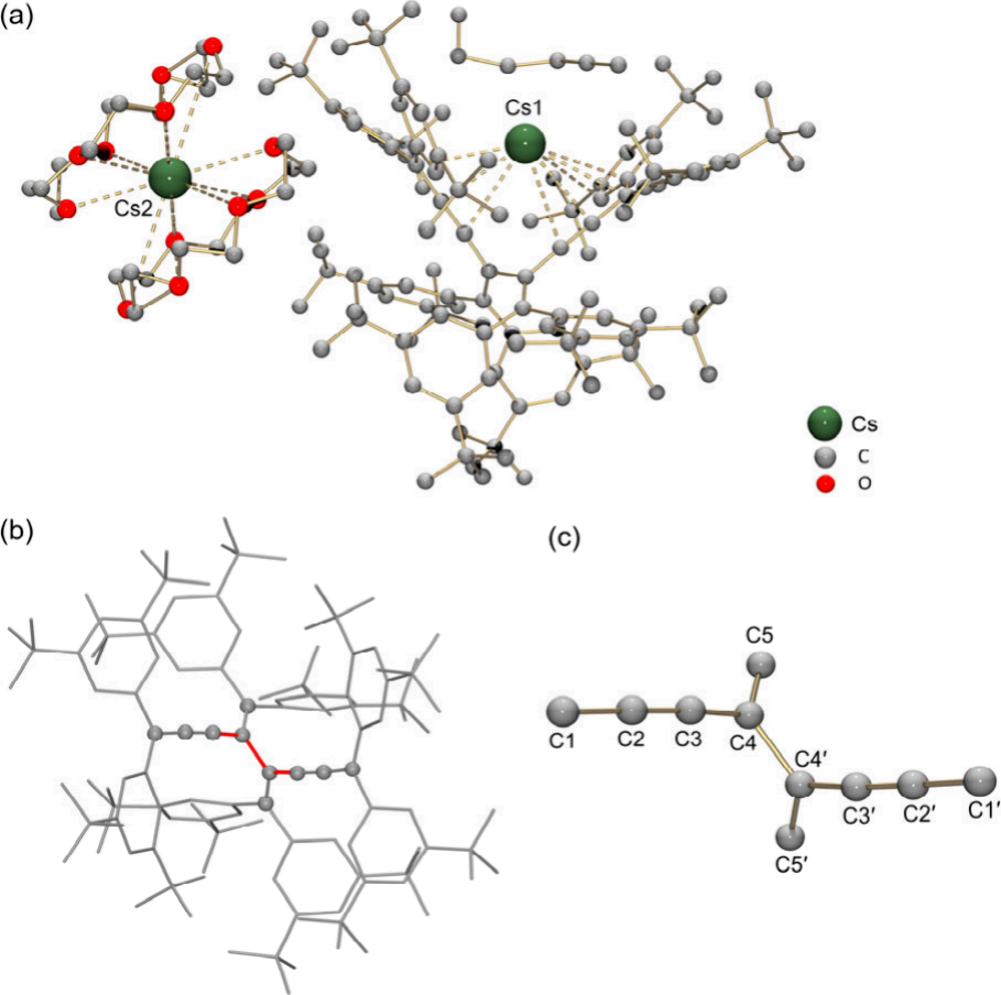
(a) Crystal structure of **3** in a ball-and-stick model, (b) the **2**_**TR**_^2–^ anion in a mixed model, and (c) its central core of **2**_**TR**_^2–^ with C-labels, ball-and-stick model. H-atoms are omitted.

Notably, reduction induces cleavage of the C3–C8 bond, converting the radialene core into a cross-conjugated dendralenic structure,^51^ an unprecedented transformation for [*n*]­radialenes (Figures 3b, c). Bond metrics of **2**_**TR**_^2–^ reflect this change: C3–C4 shortens to 1.401(9) Å, C1–C2 elongates to 1.387(10) Å, and C2–C3 becomes 1.230(9) Å (Table 1). The central single bond, C4–C4′, is somewhat elongated at 1.534(12) Å but remains in the range of structurally similar systems (1.49–1.53 Å).^15,52^ Thus, the cleavage at C4–C4′ **2_TR_^2−^** to **1** does not appear to be driven by an abnormally weak (long) bond (*vide infra*). The bond-length alternation across the C1–C5 chain resembles an extended polyyne, which is supported by the Localized Orbital Locator, LOL-π analysis (Figure S47). The LOL-π analysis is a computational tool that visualizes the spatial distribution and delocalization of π electrons, where higher values correspond to stronger localization and vice versa. Geometrically, the cumulene units in **2**_**TR**_^2–^ show slight bending (2.3–5.5°, Table S8), similar to other reduced cumulenes,^11,46^ but the torsion angle between the two cumulene subunits increases dramatically to 77.6°, compared to 8.8° in **2**, indicating a major core reorganization and potential site for direct metal coordination.

An interesting observation is that addition of a few drops of THF to hexane during the reduction of **2** afforded dark-brown plates. X-ray diffraction analysis identified complex **4**, [Cs^+^(18-crown-6)_2_]­[Cs^+^(THF)_2_(**2**_**TR**_^2–^)] (Figure 4a), structurally similar to **3** except for two THF ligands coordinated to Cs1 (Cs···C: 3.184(10)–3.839(11) Å; Cs···O_THF_: 2.608(15)/3.142(15) Å). Reducing **2** with Rb metal yielded complex **5**, [Rb^+^(18-crown-6)_2_]­[Rb^+^(**2**_**TR**_^2–^)]·C_6_H_14_ (Figure 4b), with Rb···C contacts of 3.035(5)–3.380(6) Å. Complexes **3**–**5** crystallize in the same space group (*C*2/*c*) with similar unit cell parameters and display 1D columns formed via C–H···π interactions (2.583(16)–2.629(15) Å) between anions and crown ether moieties (Figures S43–S45). The persistent geometry of **2**_**TR**_^2–^ across different products highlights its potential as a versatile host for alkali metal ions.

**Figure 4:**
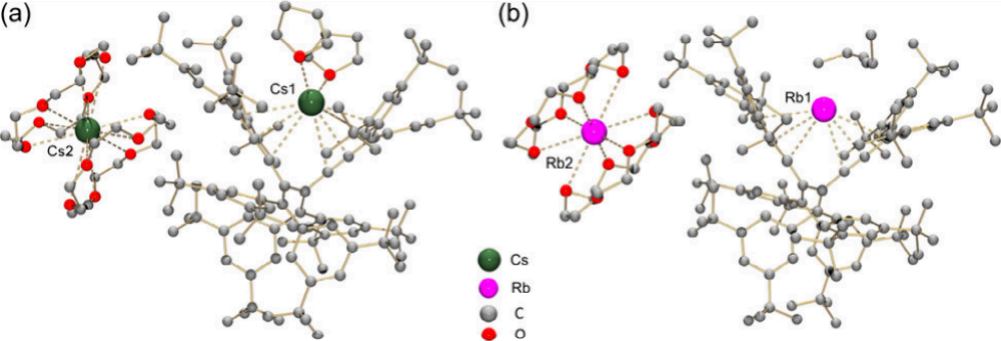
Crystal structures of (a) **4** and (b) **5**, ball-and-stick models. Cs···O_crown_: 3.147(16)–3.441(19) Å, Rb···O_crown_: 3.136(11)–3.378(16) Å. H-atoms are omitted.

To understand the core transformation, DFT calculations (B3LYP/6-31G­(d,p)) were conducted. The process was divided into three steps (Table S9, excluding reduction): (a) dimerization of cumulene **1** to give radialene **2**, (b) transformation of **2** ^*n*–^ to **2**_**TR**_^*n*–^ (*n* = 0, 1, 2), and (c) oxidation of **2**_**TR**_^2–^ back to **1**. Step (a) has a calculated Δ*G* = 30 kcal/mol, consistent with thermal dimerization of [*n*]­cumulenes,^15,23^ while step (c) is exergonic (Δ*G* = −5.8 kcal/mol, Table S9). In step (b), the transformation is thermodynamically favorable in the dianionic state. Structural analysis revealed a sharp decrease in BLA upon reduction (0.15 → 0.08 → 0.01 Å, Figure S48), indicating enhanced delocalization. LOL-π plots confirm preserved cross-conjugation^53^ in **2** and **2** ^–^ but its disruption in **2** ^2–^ (Figure S49). This breakdown of cross-conjugation, along with the unusually low BLA, suggests that **2** ^2–^ is electronically unstable. The transition-state calculations (Figure S50) further confirm the feasibility of the transformation from **2** ^2–^ to **2**_**TR**_^2–^, where the reduction lowers the energy barrier and thereby triggers the core transformation.^54^

In summary, we describe a reversible transformation between [4]­cumulene (**1**) and [4]­radialene (**2**). Heating of **1** induces solvent-free thermal dimerization to **2**, confirmed by X-ray diffraction analysis. Chemical reduction of **2** with Cs yields a novel dianionic product **2**_**TR**_^2–^ via unexpected ring-opening, isolated as complex [Cs^+^(18-crown-6)_2_]­[Cs^+^(**2**_**TR**_^2–^)]·C_6_H_14_ (**3**). Remarkably, oxidation of **3** regenerates parent cumulene **1**. The same dianion can be accessed via reduction of **1** with Cs/THF (**4**) or Rb metal (**5**) showing similar core structures. Structural analysis of **3**–**5** reveals a distinct core rearrangement with enhanced bond length alternation, resembling a polyyne. This work highlights a rare cumulene-radialene interconversion and provides new insights into redox-triggered transformations of carbon-rich π-frameworks.

## Supplementary Material



## Data Availability

The data underlying this study are available in the published article and its Supporting Information.

## Notes

Full experimental details, UV–vis and ^1^H NMR spectra, X-ray crystallographic data for all prepared compounds, and computational details (PDF)
