# Supplementary material for: Make it Double: [4]Cumulene Thermal Core Transformation and Chemical Reduction
Source: Org Lett. 2025 Sep 23;27(39):10993–8. doi: 10.1021/acs.orglett.5c03034 (PMC12501943; doi:10.1021/acs.orglett.5c03034)
Supplement: Supplementary file 1 [file ol5c03034_si_001.pdf]

Make it Double: [4]Cumulene Thermal Core Transformation and Chemical Reduction

Zheng Zhou,[a,b] Zheng Wei,[a] Matthew A. Johnson,[c] Yikun Zhu,[a] Yu Qiu,[c] Rik R. Tykwinski,[c,*] Marina A. Petrukhina[a,*]

[a] Department of Chemistry, University at Albany, State University of New York, Albany, NY 12222, USA

[b] School of Materials Science and Engineering, Tongji University, Shanghai 201804, China

[c] Department of Chemistry, University of Alberta, Edmonton, Alberta, Canada T6G 2G2

*mpetrukhina@albany.edu, rik.tykwinski@ualberta.ca

Supporting Information

I. **Materials and Methods** 2

II. **UV-vis Spectroscopic Investigation** 5

III. **NMR Spectroscopic Investigation** 7

IV. **Redox Reversibility Test** 16

V. **Powder X-Ray Diffraction Investigation** 17

VI. **Thermal Analysis Investigations** 19

VII. **Crystal Structure Solution and Refinement Details** 27

VIII. **Computational Details** 36

IX. **References** 63

I. Materials and Methods

All manipulations were carried out using break-and-seal[1] and glove-box techniques under an atmosphere of argon. Tetrahydrofuran (THF) and hexane (Sigma Aldrich) were dried over Na/benzophenone and distilled prior to use. Cesium metal (98%), rubidium metal (98%), and 18-crown-6 ether (99%) were purchased from Sigma Aldrich and used as received. Tetrahydrofuran-d_8 (Sigma Aldrich) was dried over NaK_2 alloy and distilled prior to use. The sample of **1** was synthesized according to the reported procedure.[2] UV-Vis absorption spectra were recorded on a Thermo Scientific Evolution 201 UV-Visible Spectrophotometer. NMR spectra were measured using a Bruker Ascend-500 spectrometer (500 MHz for ^{1}H, 126 MHz for ^{13}C, 104 MHz for ^{133}Cs) with a 5 mm BBO probe and referenced to the resonances of the solvent used. Low-temperature NMR experiment was controlled by a Cryo Diffusion cryogenic tank probe, and liquid N_2 was used as a cooling source. The temperature correction for –40 °C~–80 °C was calibrated by pure MeOH. The cooling power was set as 15~35% (15% at 0 °C and 35% at –80 °C). The X-ray powder diffraction data of **1** and **2** were collected on a Bruker D8 Advance diffractometer (Cu Kα radiation, focusing Göbel Mirror, LynxEye one-dimensional detector, a step of 0.02° 2θ, 20 °C). Le Bail fit for powder diffraction patterns was performed using TOPAS, version 4 software package (Bruker AXS, 2006). For the *ex-situ* powder XRD analysis, data were collected on a Rigaku multipurpose *θ-θ* X-ray SmartLab SE diffractometer (Cu Kα radiation, HyPix-400 two-dimensional advanced photon counting hybrid pixel array detector, step of 0.01° 2*θ* for **1** before heating and 0.005° 2*θ* for **1** after heating, 20 °C). Le Bail fit for powder diffraction patterns has been performed using TOPAS version 5 software package (Bruker AXS, 2014).

Sublimation of 1 to afford $C_{122}H_{168}$ (2)

5 mg of **1** was sealed in a glass ampule (L. 15 cm, O.D. 1 cm) under vacuum and placed into an electric oven having a small temperature gradient along the length of the tube. The temperature was set at 200 °C. In 2 days, the traces of solid and oil were observed in the cold zone of the tube. The yellow-orange solid from the hot zone was collected and sealed in a new glass ampule (L. 10 cm, O.D. 1 cm) which was placed in the oven with the temperature set at 235 °C. After 5 days, orange block-shaped crystals were present in high yield. Yield: 4.5 mg, 90%. UV-Vis (THF, nm): λ_{max} 290, 460. ^{1}H NMR (THF-d_8, –60 °C, ppm): δ 6.15–7.98 (24H, $C_{122}H_{168}$), 0.93–1.24 (144H,

$C_{122}H_{168}$). ^{13}C NMR (THF-d_8, 25 °C, ppm): δ 149.03, 140.78, 140.37, 138.82, 136.88, 136.10, 122.28, 120.15, 118.56, 111.47, 34.32, 31.04, 30.77.

[Cs^+(18-crown-6)$_2$][Cs^+($C_{122}H_{168}^{2-}$)]·C_6H_{14} (3)

Hexane (1.5 mL) was added to a customized glass system containing excess Cs metal (2 mg, 0.015 mmol) and **2** (2 mg, 0.0018 mmol). The mixture was allowed to stir under argon at 25 ˚C in a closed system. The initial color of the suspension was pale orange (neutral ligand), then it changed to green-brown after 3 minutes, and further deepened to purple-brown after 15 minutes. After 24 hours of stirring, the suspension was filtered, and the purple-brown filtrate was treated with 18-crown-6 ether (1 mg, 0.0038 mmol). The L-shaped ampule was sealed and placed near a sand bath for slow evaporation. Brown plates were present in good yield after 5 days. Yield: 3.2 mg, 66%. UV-Vis (THF, nm): λ_{max} 459, 542, 726. ^1H NMR (THF-d_8, –60 °C, ppm): δ 6.21–8.55 (48H, $C_{122}H_{168}^{2-}$), 0.93–1.46 (288H, $C_{122}H_{168}^{2-}$). ^{13}C NMR (THF-d_8, 25 °C, ppm): δ 147.81, 146.23, 145.64, 145.60, 145.05, 127.09, 125.37, 124.71, 119.71, 117.29, 116.46, 116.46, 116.20, 11.35, 107.44, 34.48, 34.23, 34.21, 31.74, 31.68, 31.61, 31.58, 31.55, 31.33. ^{133}Cs NMR (THF-d_8, 25 °C, ppm): δ 1.69, –44.21.

[Cs^+(18-crown-6)$_2$][Cs^+($C_{122}H_{168}^{2-}$)(THF)$_2$] (4)

Hexane (1.5 mL) and two drops of THF were added to a customized glass system containing excess Cs metal (2 mg, 0.015 mmol) and **2** (2 mg, 0.0018 mmol). The mixture was allowed to stir under argon at 25 ˚C in a closed system. The initial color of the suspension was pale orange (neutral ligand), then it changed to green-brown after 3 minutes, and further deepened to purple brown after 15 minutes. After 24 hours of stirring, the suspension was filtered, and the purple-brown filtrate was treated with 18-crown-6 ether (1 mg, 0.0038 mmol)' The L-shaped ampule was sealed and placed near a sand bath for slow evaporation. Brown plates were present in good yield after 6 days. Yield: 2.9 mg, 60%. UV-Vis (THF, nm): λ_{max} 458, 540, 726.

[Rb^+(18-crown-6)$_2$][Rb^+($C_{122}H_{168}^{2-}$)]·C_6H_{14} (5)

Hexane (1.5 mL) was added to a customized glass system containing excess Rb metal (2 mg, 0.029 mmol) and **2** (2 mg, 0.0018 mmol). The mixture was allowed to stir under argon at 25 ˚C for 24 hours in a closed system. The initial color of the suspension was pale orange (neutral ligand), then

it changed to green-brown after 5 minutes, and further deepened to purple brown after 20 minutes. After 24 hours of stirring, the suspension was filtered, and the purple-brown filtrate was treated with 18-crown-6 ether (1 mg, 0.0038 mmol). The L-shaped ampule was sealed and placed near a sand bath for slow evaporation. Brown plates were present in good yield after 5 days. Yield: 2.8 mg, 60%. UV-Vis (THF, nm): λ_{max} 447, 534, 715.

II. UV-vis Spectroscopic Investigation

Sample preparation: THF (2 mL) was added to a glass ampule (O.D. 1 cm) containing excess Cs metal (~30 eq.) and ~0.2 mg of **2** (1.2×10^{-4} mmol). The ampule was sealed under argon and UV-vis absorption spectra were monitored at different reaction times (total 24 hours) at room temperature.

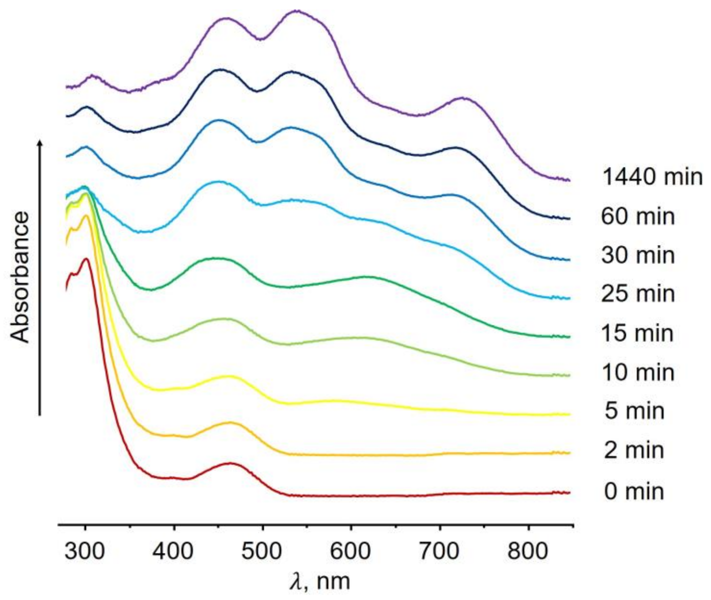

Figure S1. UV-vis spectra of **2**/Cs in hexane.

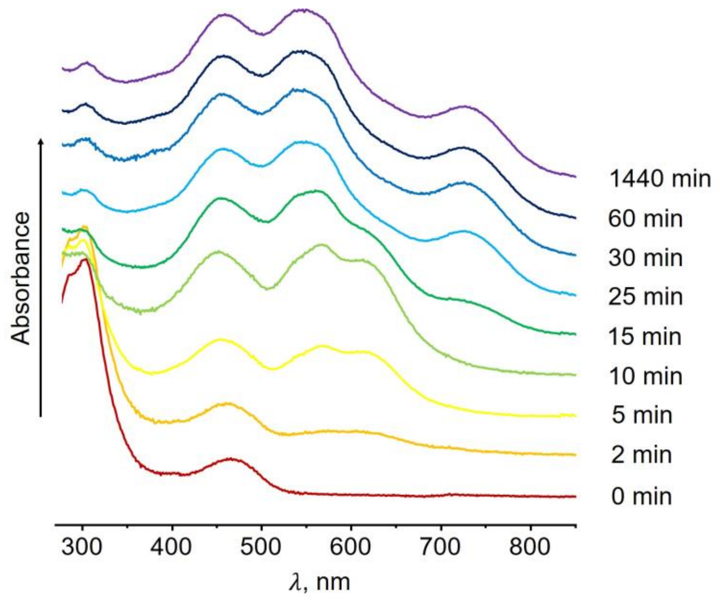

Figure S2. UV-vis absorption spectra of **2**/Cs/18-crown-6 in hexane.

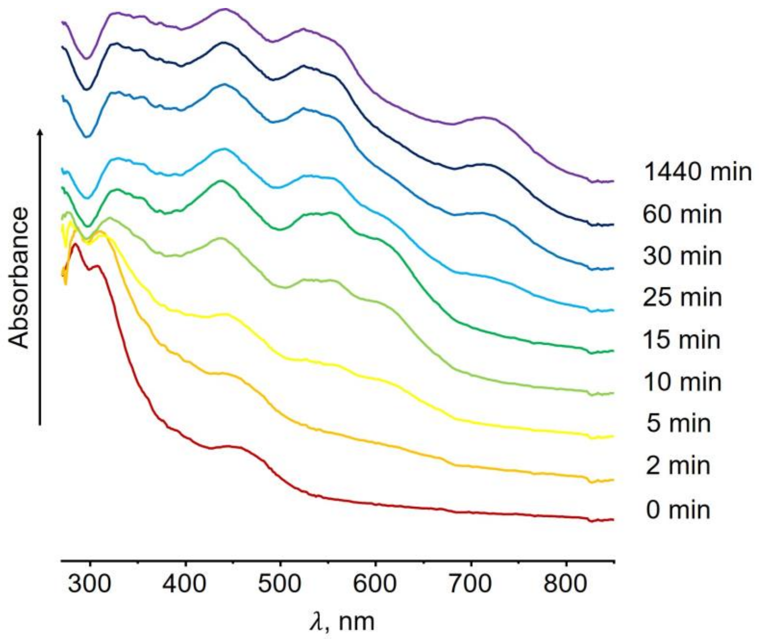

Figure S3. UV-vis absorption spectra of **2**/Rb/18-crown-6 in hexane.

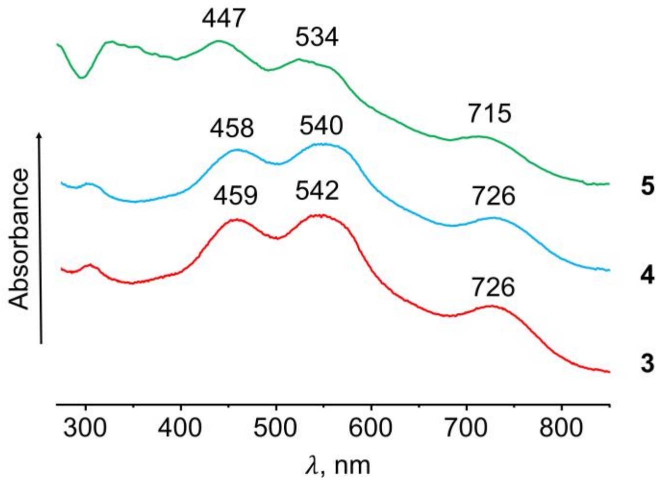

Figure S4. UV-vis absorption spectra of **3**–**5** dissolved in THF.

III. NMR Spectroscopic Investigation

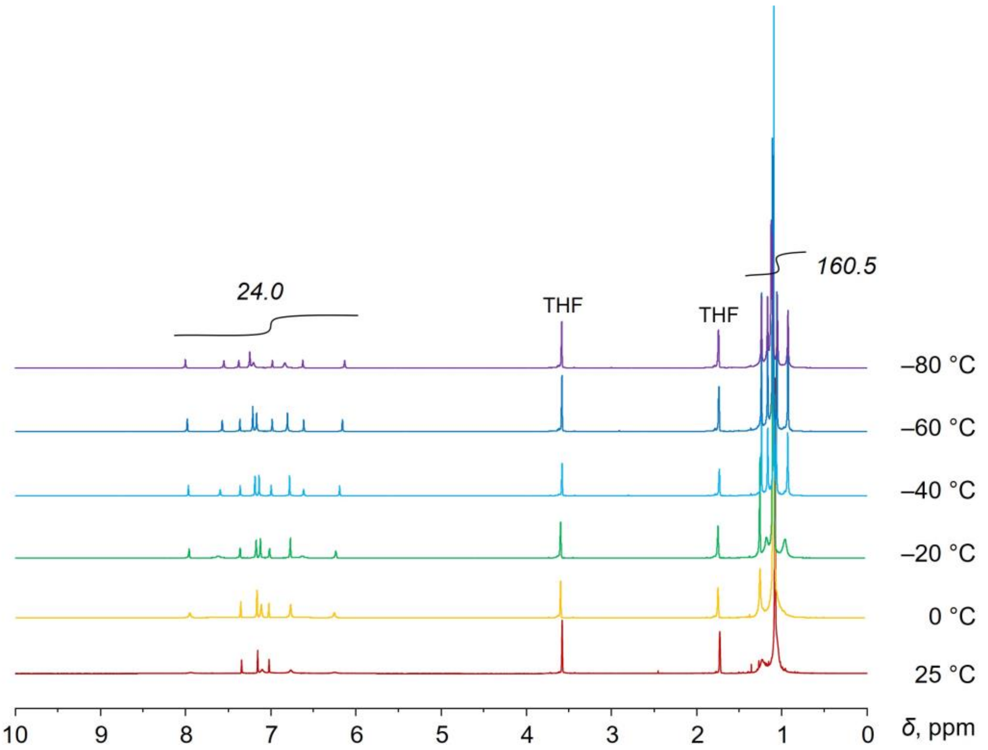

Figure S5. Variable-temperature 500 MHz ^{1}H NMR spectra of **2** in THF-d_8.

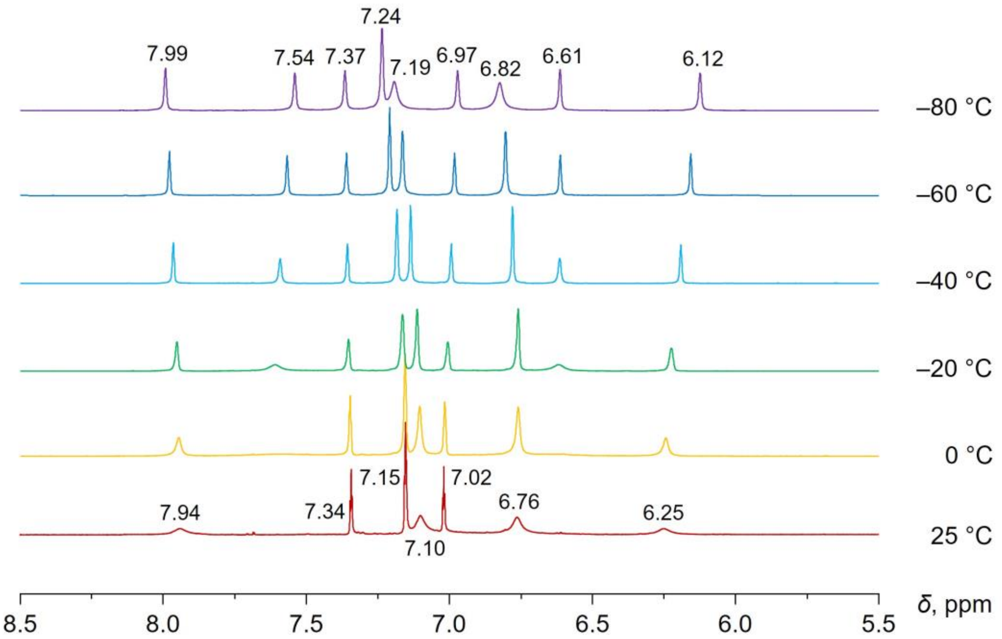

Figure S6. Variable-temperature 500 MHz ^{1}H NMR spectra of **2** in THF-d_8, aromatic region.

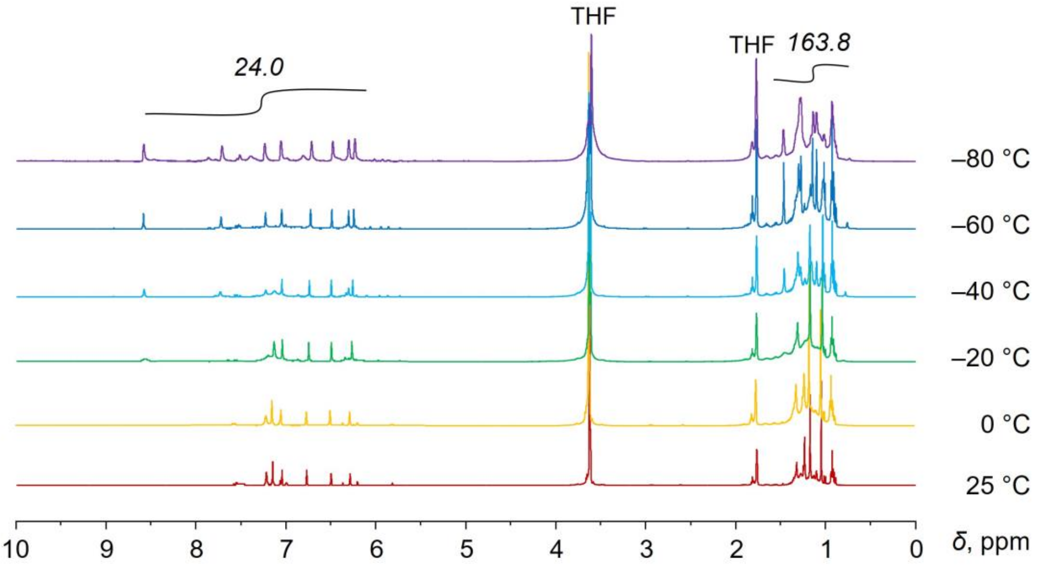

Figure S7. Variable-temperature 500 MHz ^{1}H NMR spectra of **3** in THF-d_8.

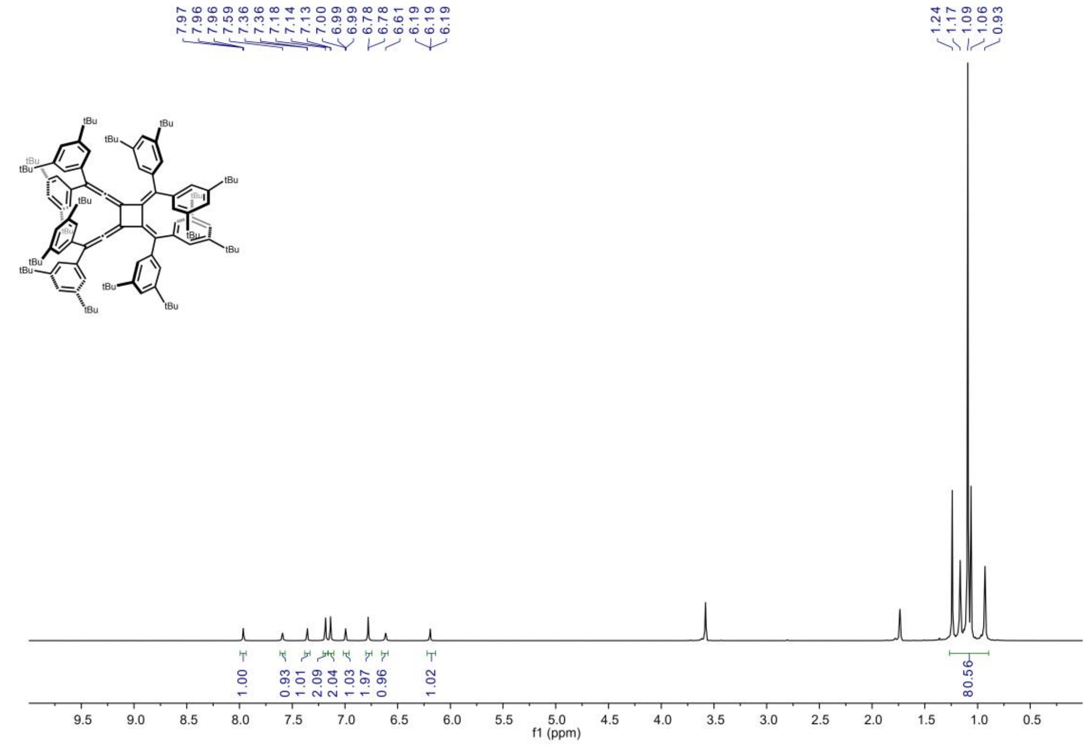

Figure S8. 500 MHz ^{1}H NMR spectrum of **2** in THF-d_8 at 25 °C.

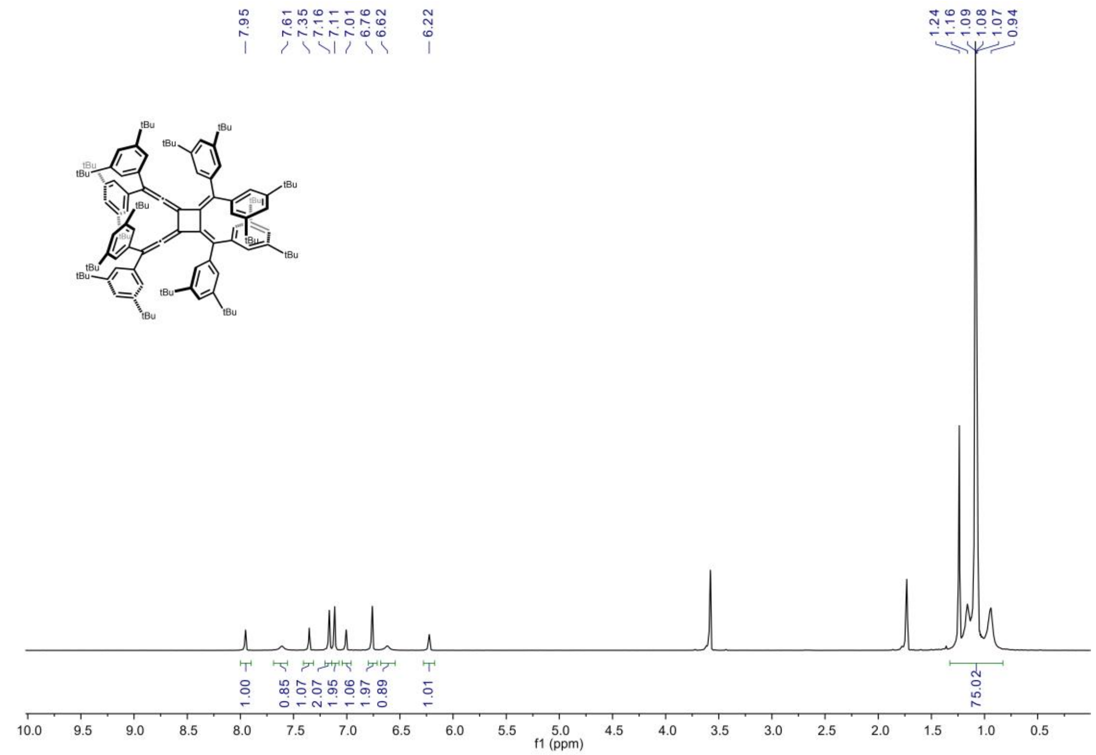

Figure S9. 500 MHz ^{1}H NMR spectrum of **2** in THF-d_8 at 0 °C.

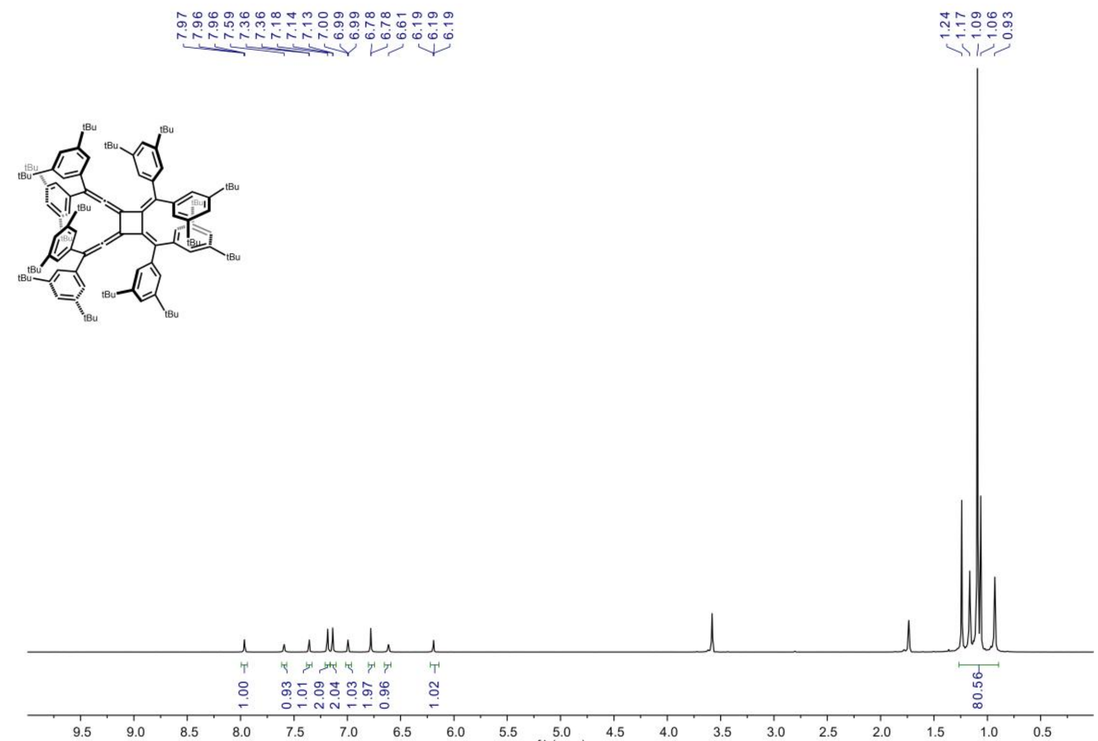

Figure S10. 500 MHz ^{1}H NMR spectrum of **2** in THF-d_8 at –20 °C.

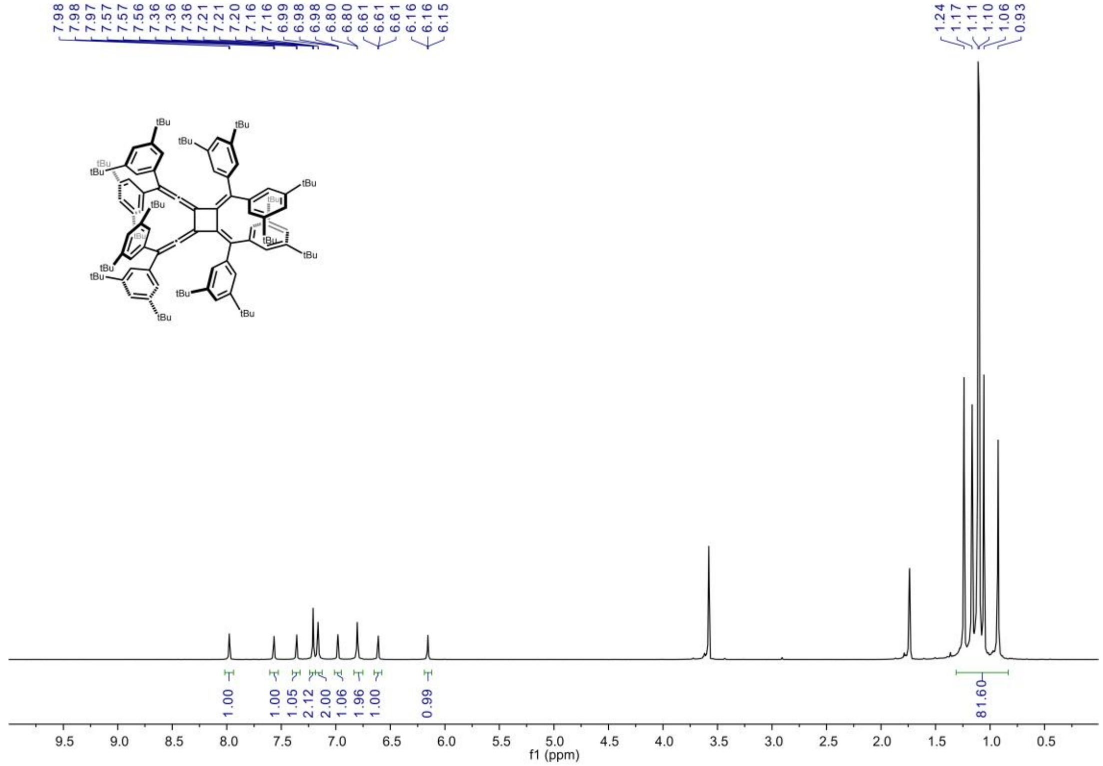

.

Figure S11. 500 MHz ^{1}H NMR spectrum of **2** in THF-d_8 at –40 °C.

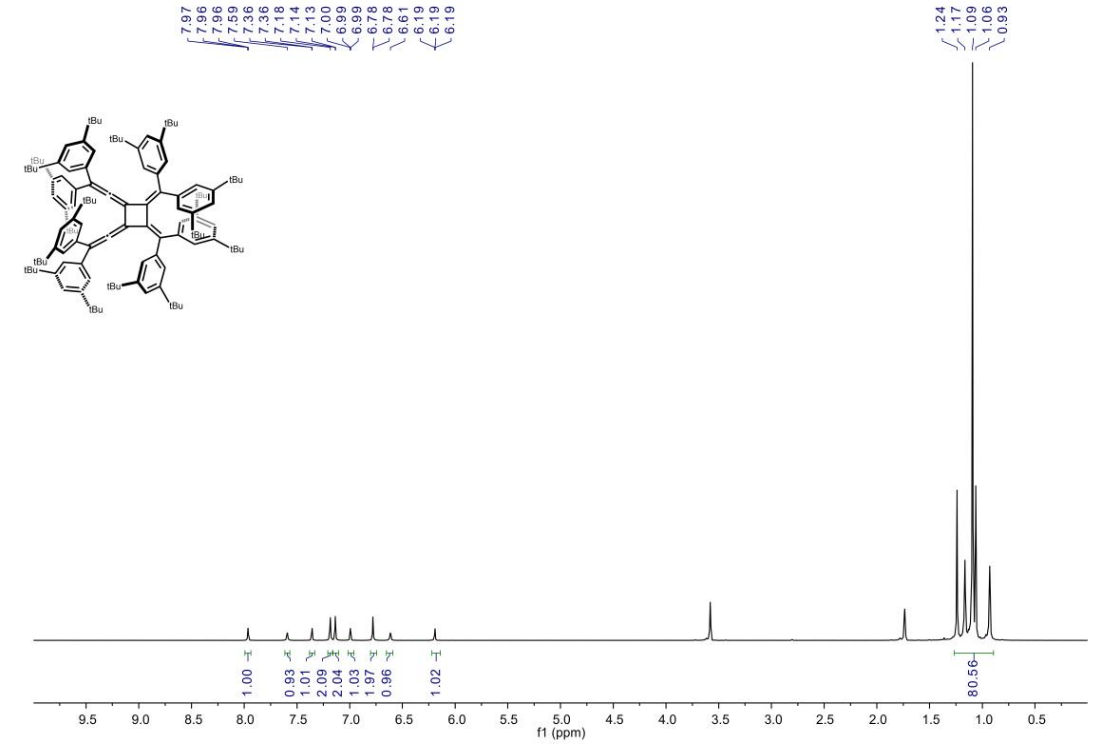

Figure S12. 500 MHz ^{1}H NMR spectrum of **2** in THF-d_8 at –60 °C.

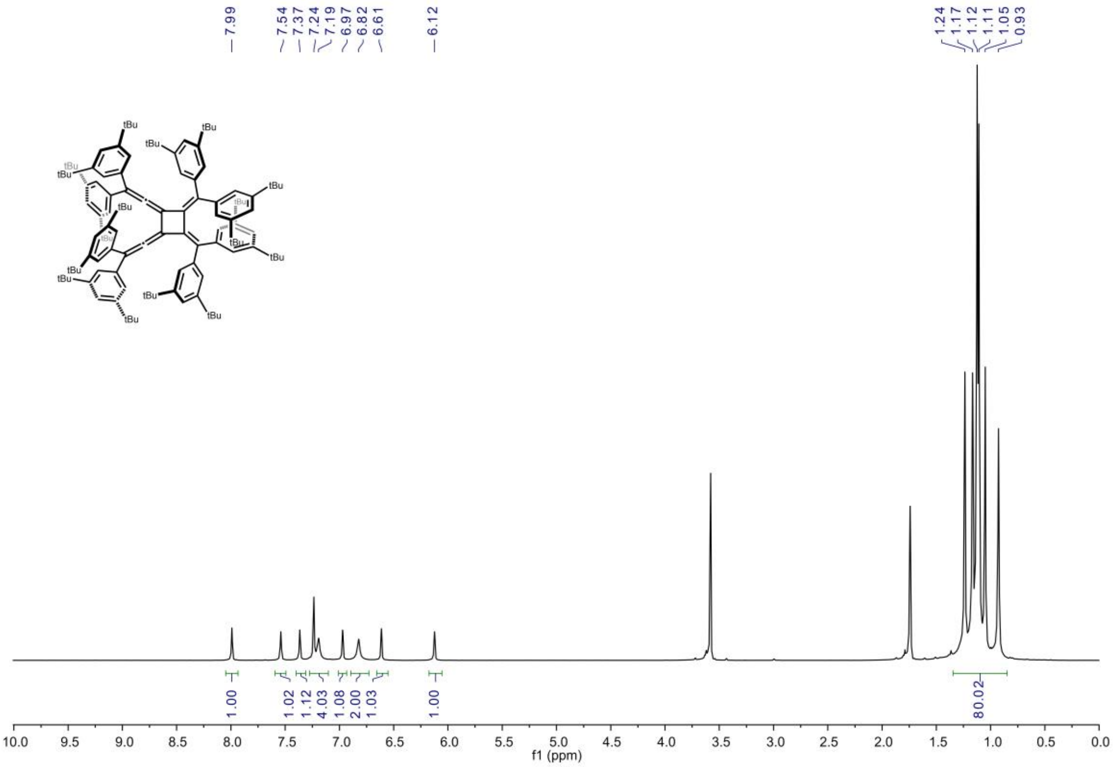

Figure S13. 500 MHz ^{1}H NMR spectrum of **2** in THF-d_8 at –80 °C.

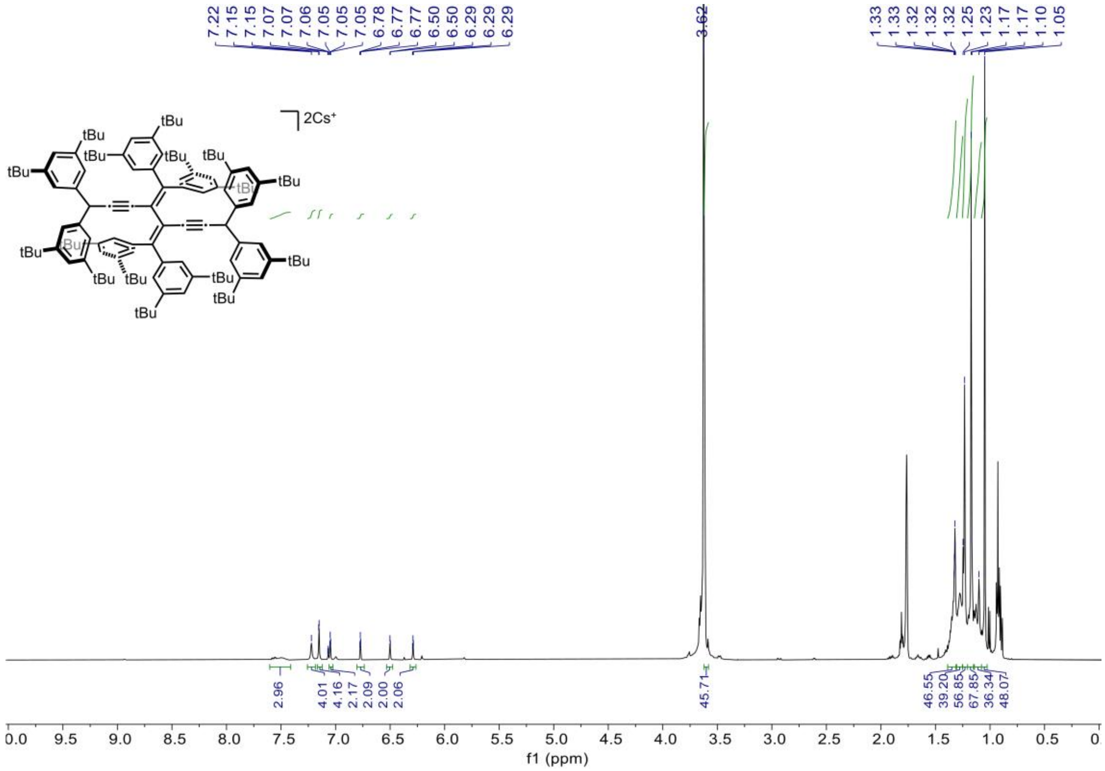

Figure S14. 500 MHz ^{1}H NMR spectrum of **3** in THF-d_8 at 25 °C.

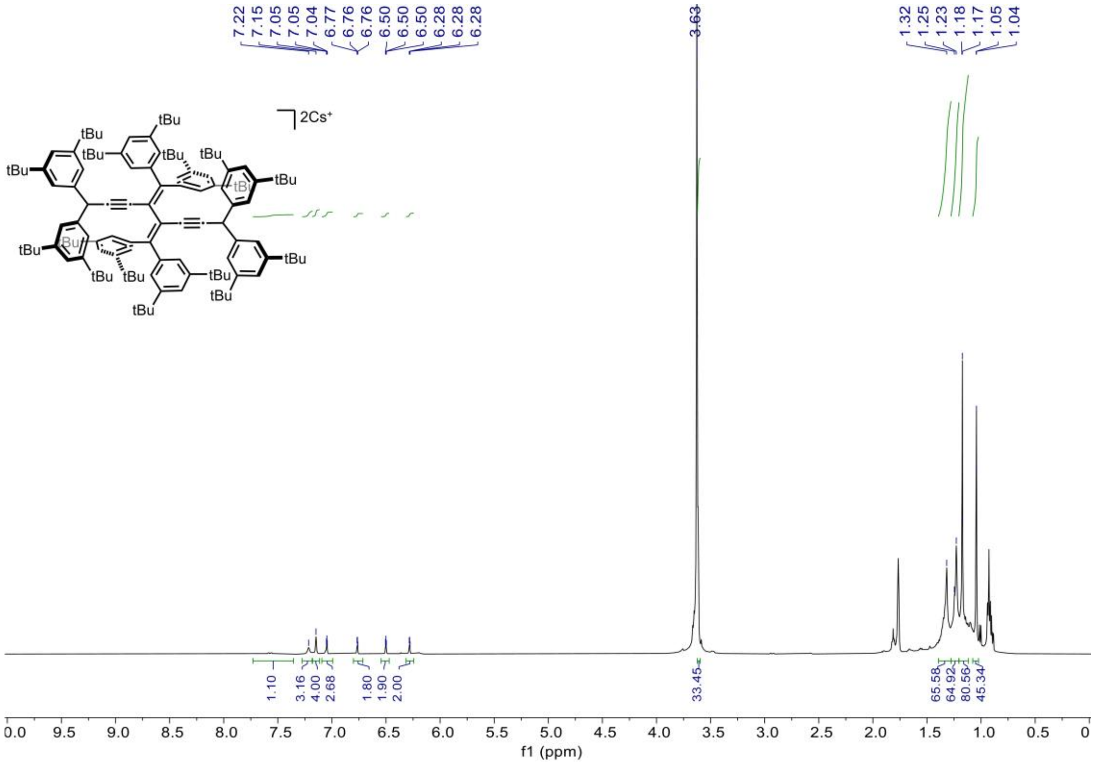

Figure S15. 500 MHz ^{1}H NMR spectrum of **3** in THF-d_8 at 0 °C.

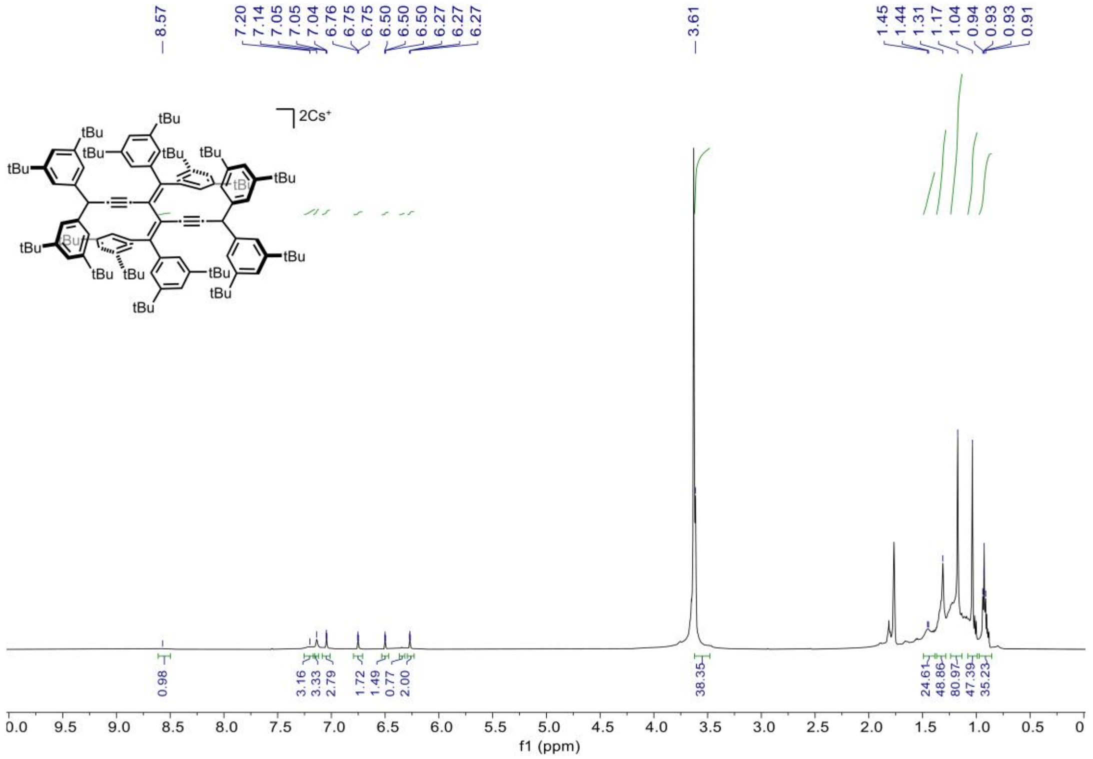

Figure S16. 500 MHz ^{1}H NMR spectrum of **2** in THF-d_8 at –20 °C.

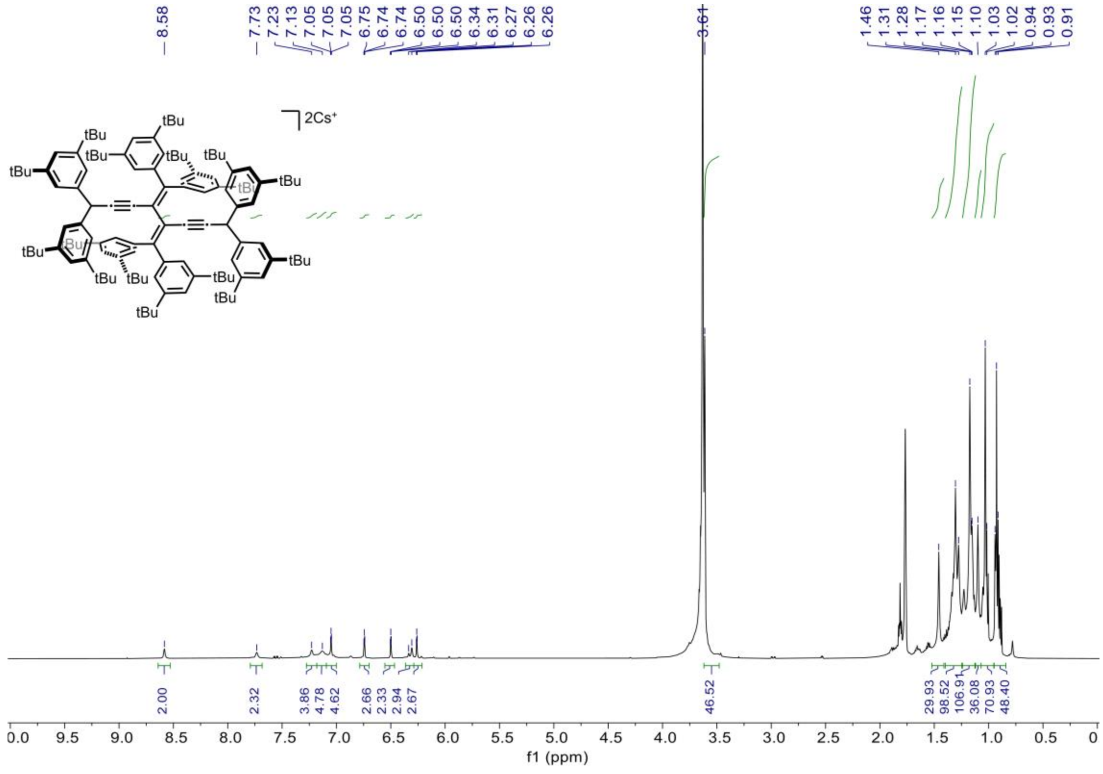

Figure S17. 500 MHz ^{1}H NMR spectrum of **2** in THF-d_8 at –40 °C.

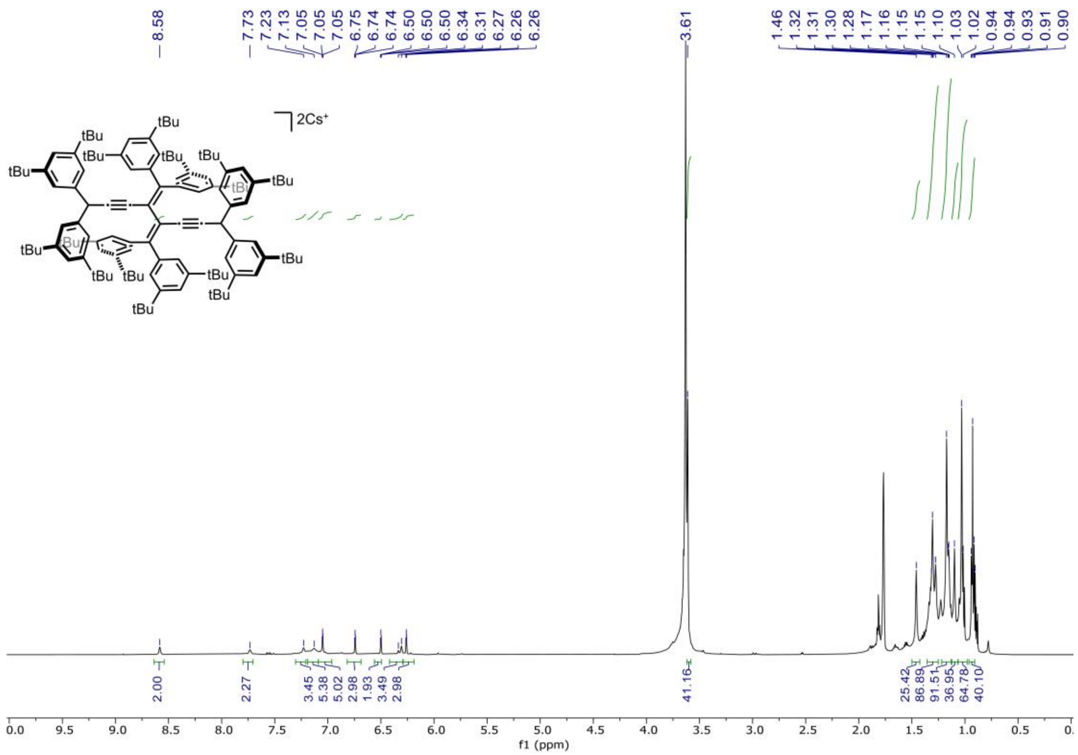

Figure S18. 500 MHz ^{1}H NMR spectrum of **3** in THF-d_8 at –60 °C.

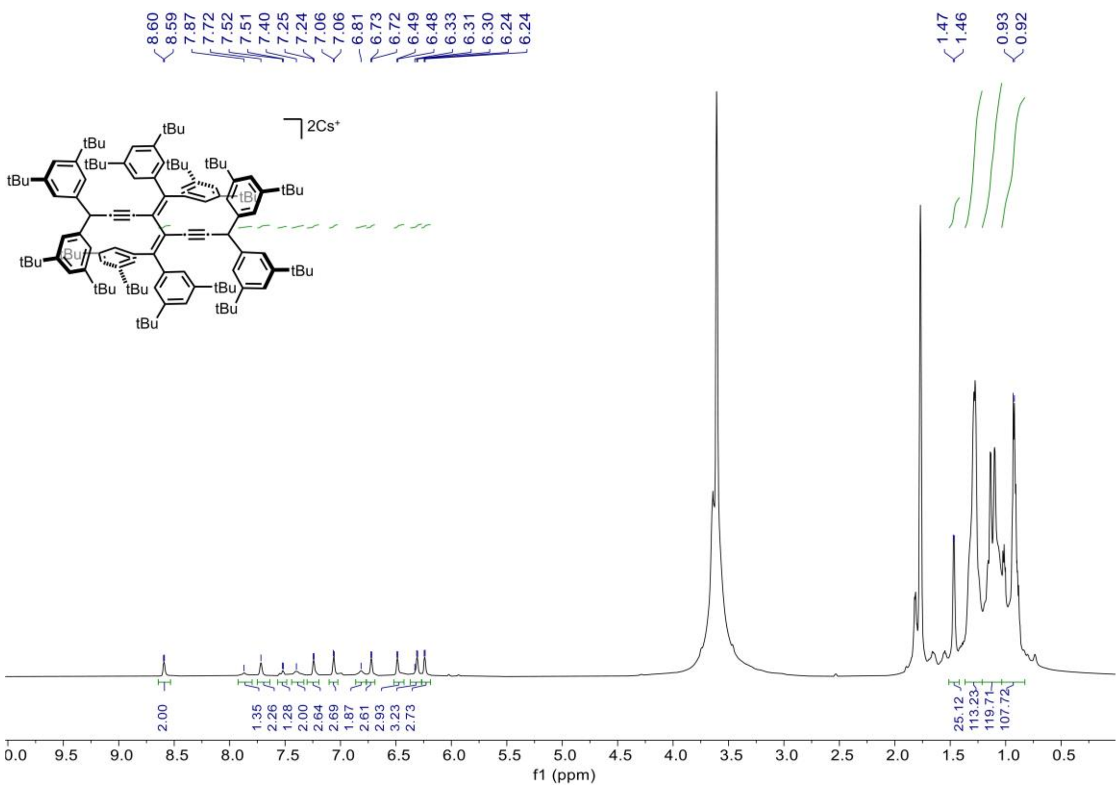

Figure S19. 500 MHz ^{1}H NMR spectrum of **3** in THF-d_8 at –80 °C.

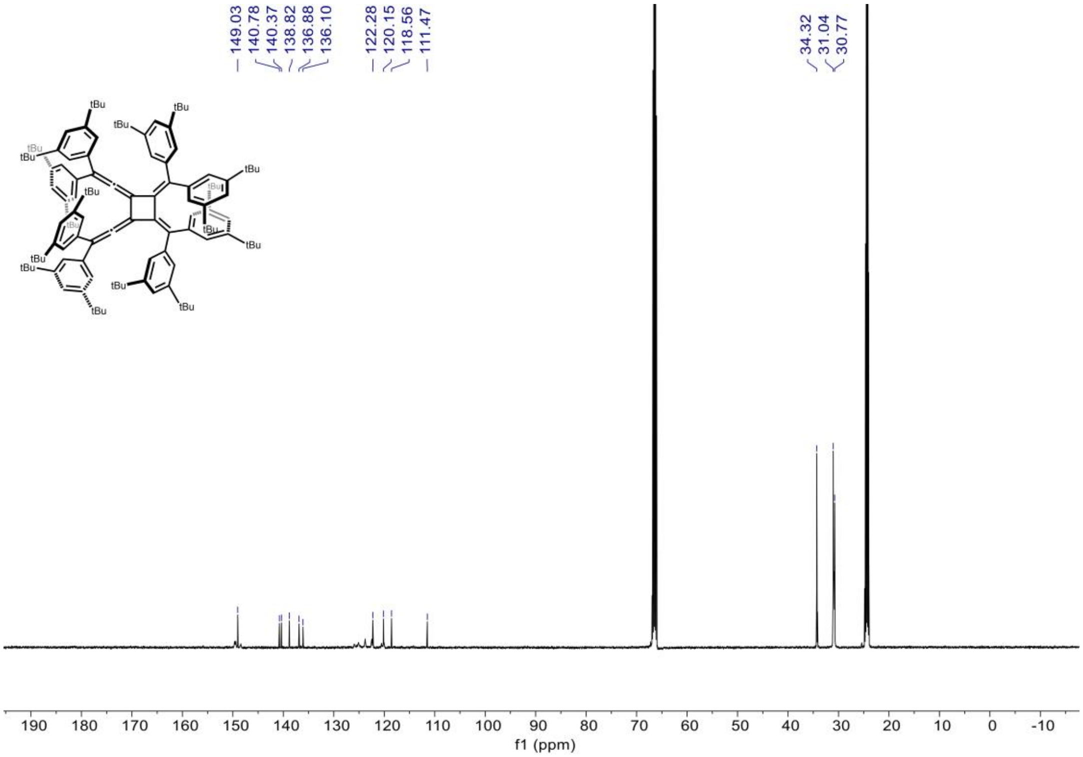

Figure S20. 126 MHz ^{13}C NMR spectrum of **2** in THF-d_8 at 25 °C.

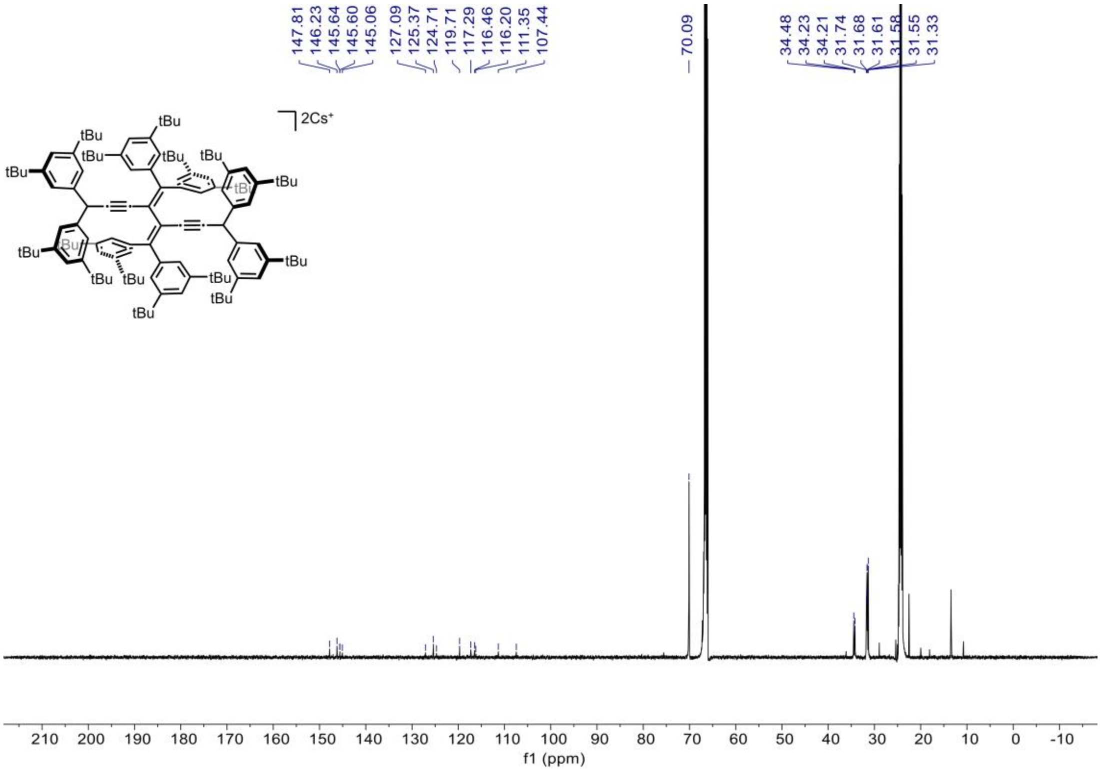

Figure S21. 126 MHz ^{13}C NMR spectrum of **3** in THF-d_8 at 25 °C.

IV. Redox Reversibility Test

Sample preparation: Crystals of **2** (2 mg, 0.0024 mmol), Cs metal (~2.8 mg, 0.072 mmol, 30 eq.) and 18-crown-6 ether (3 mg, 0.011 mmol) were added into an NMR tube, followed by addition of 0.7 mL of fresh THF-d_8. The tube was sealed under argon. The ^{1}H NMR spectrum of **2** was collected immediately, and that of *in situ* generated **3** was collected after 30 minutes. The solution was then exposed to air by opening the tube, and its spectrum was recorded as "quenched".

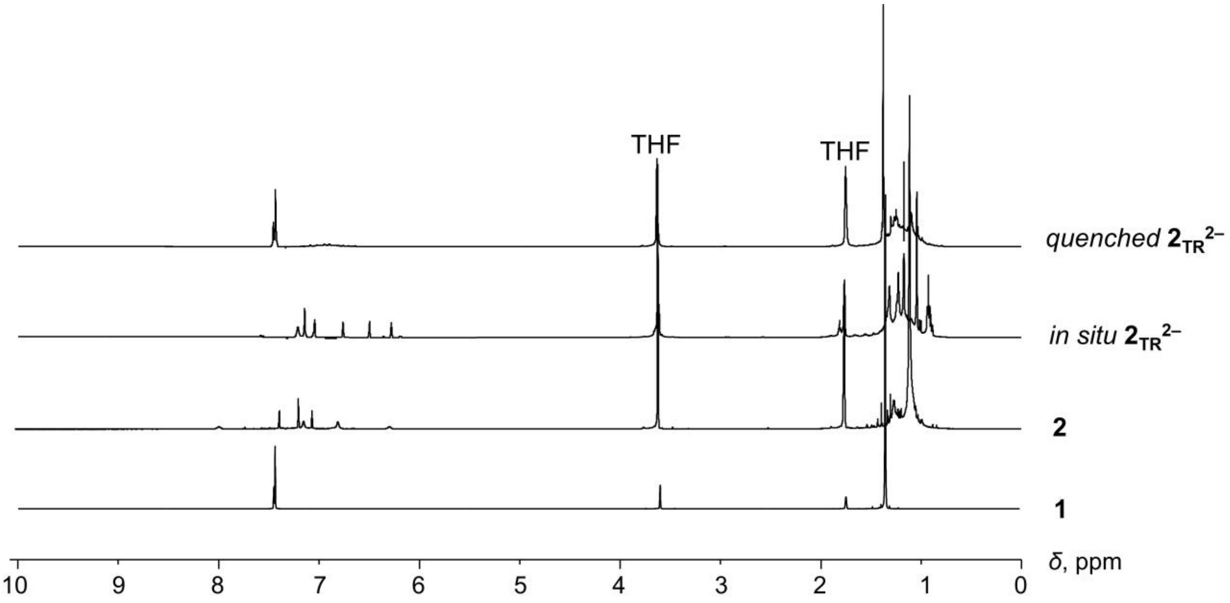

Figure S22. 500 MHz ^{1}H NMR spectra of **1**, **2**, *in situ* generated $\mathbf{2_{TR}}^{2-}$, and its air quenched product in THF-d_8 at 25 °C.

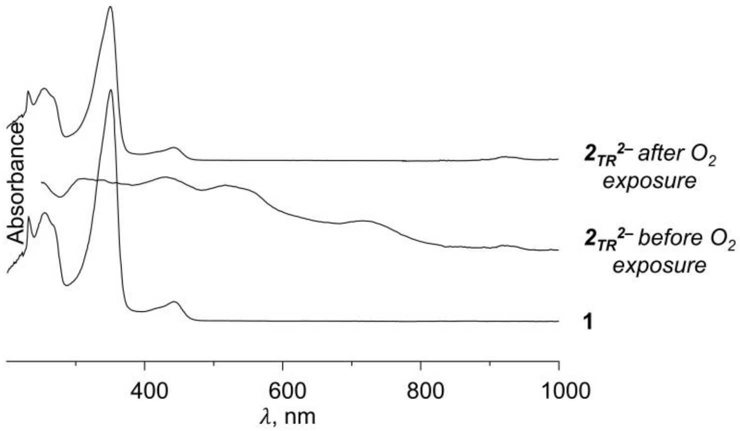

Figure S23. UV-vis absorption spectra of **1** and *in situ* generated $\mathbf{2_{TR}}^{2-}$ before and after O_2 exposure in THF at 25 °C.

V. Powder X-Ray Diffraction Investigation

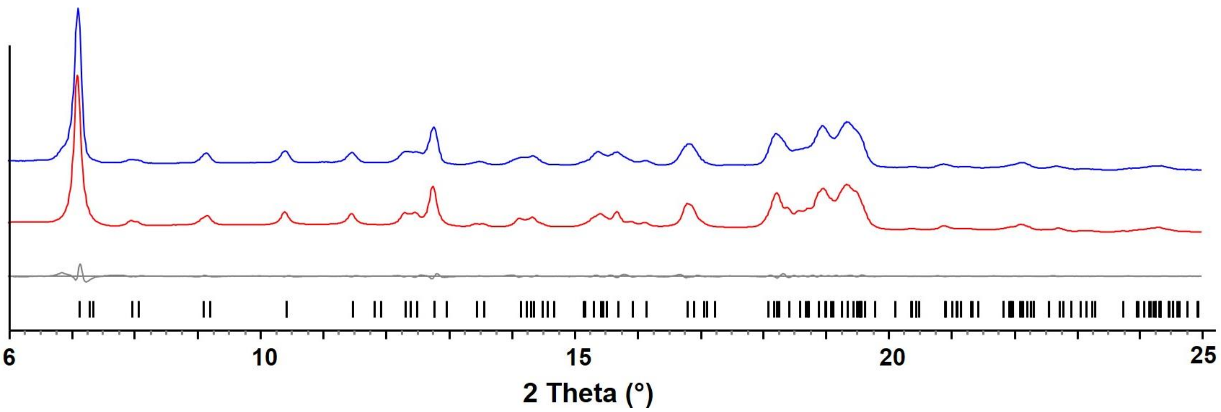

Figure S24. X-ray powder diffraction pattern of **1** and Le Bail fit. Blue and red lines are experimental and calculated patterns, respectively. Grey is the difference line with theoretical positions shown at the bottom in black.

Table S1. Cell parameters comparison of **1** from single crystal analysis and Le Bail fit.

**1**		
	Single crystal data (173K)	Le Bail fit data (298K)
Space Group	*P*–1	
a	14.1241(5) Å	14.3090(23) Å
b	14.4546(5) Å	14.5331(23) Å
c	16.2393(6) Å	16.1660(19) Å
α	72.675(2)°	72.367(10)°
β	81.918(2)°	81.851(10)°
γ	61.163(2)°	60.210(8)°
V	2772.5(2) Å^3	2780.2(8) Å^3

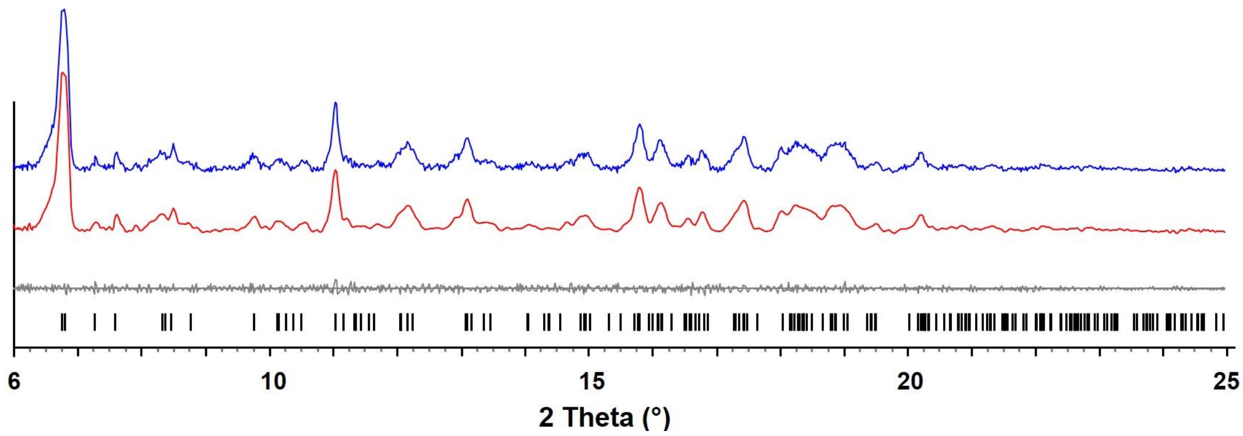

Figure S25. X-ray powder diffraction pattern of **2** and Le Bail fit. Blue and red lines are experimental and calculated patterns, respectively. Grey is the difference line with theoretical positions shown at the bottom in black.

Table S2. Cell parameters comparison of **2** from single crystal analysis and Le Bail fit.

**2**		
	Single crystal data (100K)	Le Bail fit data (298K)
Space Group	$P2_12_12_1$	
a	17.0752(3) Å	17.069(27) Å
b	17.1948(3) Å	17.279(29) Å
c	38.0274(7) Å	40.031(24) Å
α	90°	90°
β	90°	90°
γ	90°	90°
V	11165(1) Å^3	11807(33) Å^3

VI. Thermal Analysis Investigations

Sample preparation: A sample of **1** (10 mg) was checked by powder X-ray diffraction first, then it was transferred into a Schlenk tube under argon atmosphere. The Schlenk tube was heated in a sand bath at 230 °C under argon. The yellow color of **1** quickly darkened into orange. After 5 minutes, the Schlenk tube was removed from the sand bath and cooled to room temperature. The orange-red powder was collected and used for powder X-ray diffraction analysis.

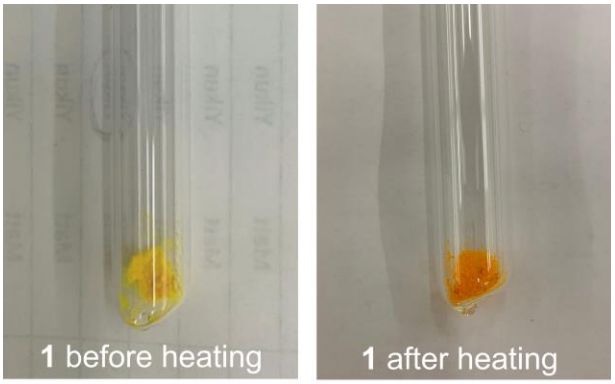

Figure S26. Colors of solid-state reactions before and after heating.

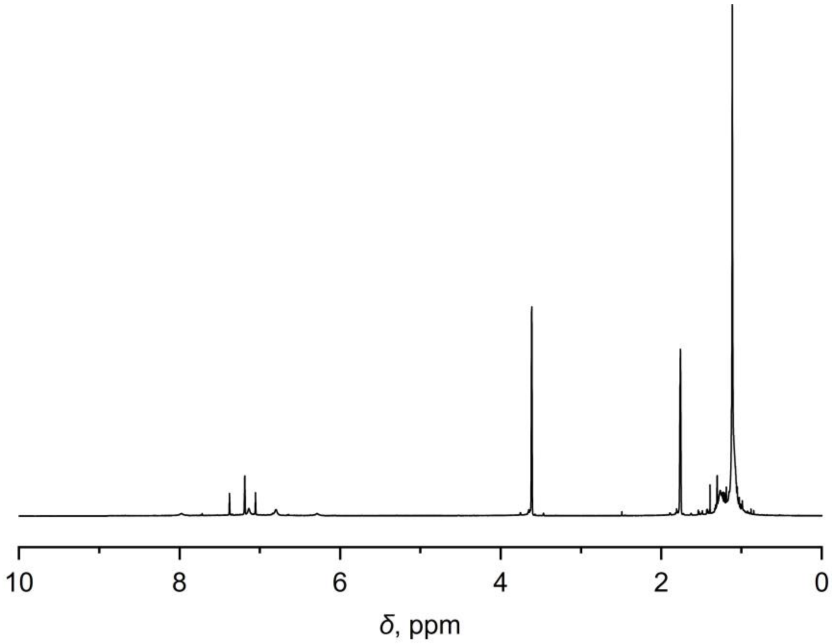

Figure S27. 500 MHz ^{1}H NMR spectrum in THF-d_8 after heating **1** at 235 °C.

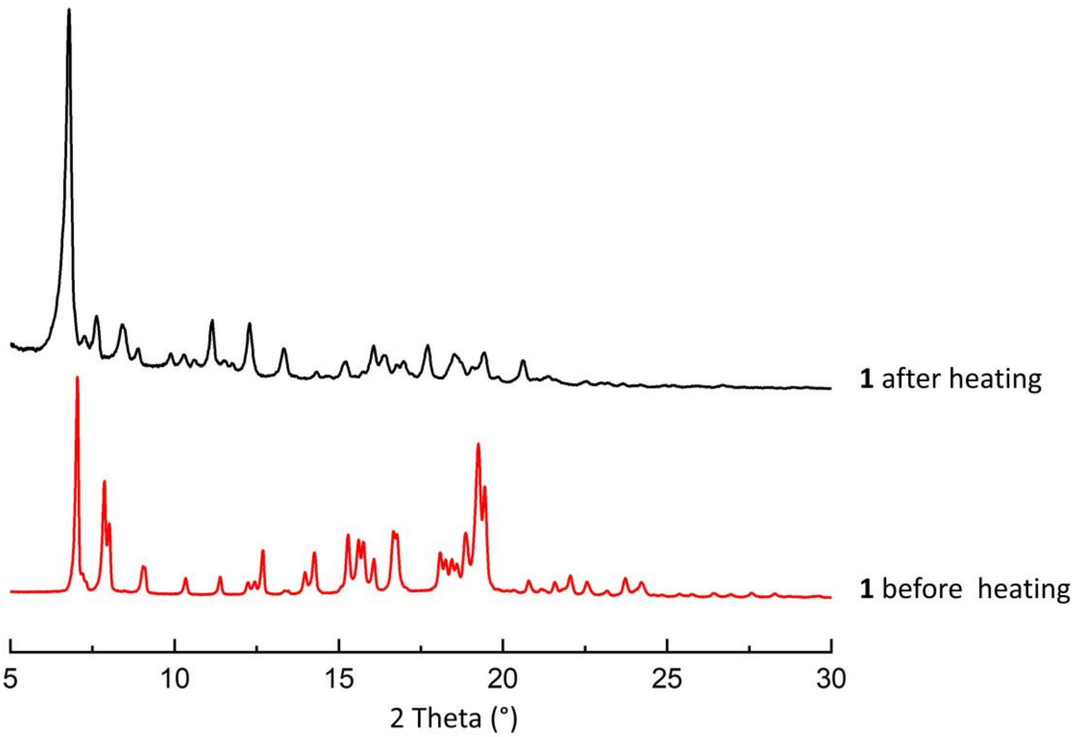

Figure S28. *Ex-situ* powder XRD spectra of **1** before and after heating.

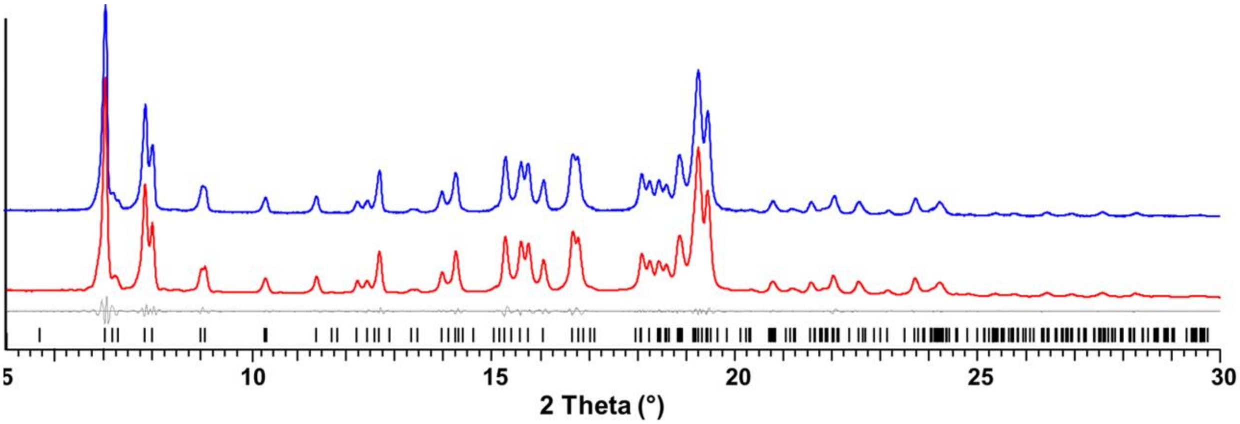

Figure S29. X-ray powder diffraction pattern of **1** before heating and Le Bail fit. Blue and red lines are experimental and calculated patterns, respectively. Grey is the difference line with theoretical positions shown at the bottom in black.

Table S3. Cell parameters comparison of Le Bail fit data of **1** before heating with single crystal data of **1**.

	**1**	**1** before heating
	Single crystal data (173 K)	Le Bail fit data (298 K)
Space Group	*P*–1	
a	14.1241(5) Å	14.3080(13) Å
b	14.4546(5) Å	14.5341(13) Å
c	16.2393(6) Å	16.1670(14) Å
α	72.675(2)°	72.347(10)°
β	81.918(2)°	81.871(10)°
γ	61.163(2)°	60.220(8) °
V	2772.5(2) Å^3	2780.4(9) Å^3

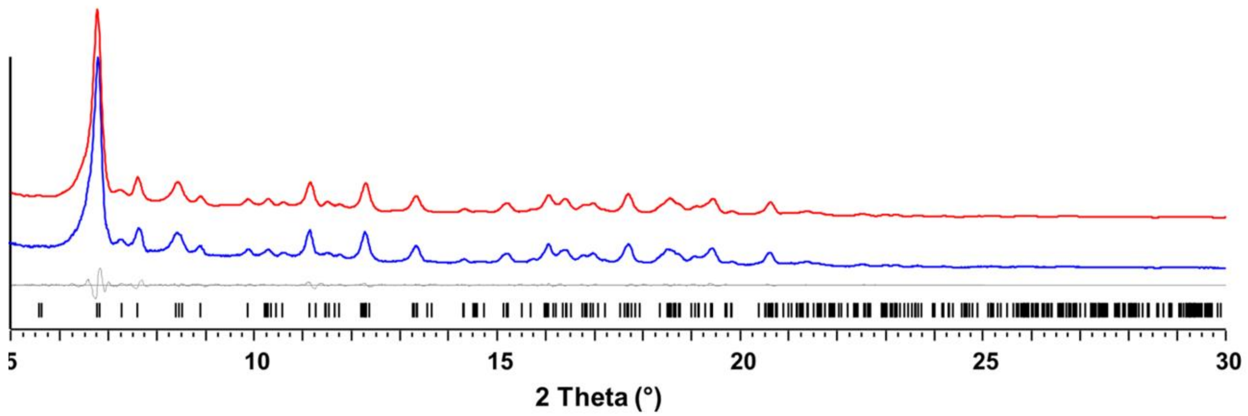

Figure S30. X-ray powder diffraction pattern of **1** after heating and Le Bail fit. Blue and red lines are experimental and calculated patterns, respectively. Grey is the difference line with theoretical positions shown at the bottom in black.

Table S4. Cell parameters comparison of Le Bail fit data of **1** after heating with single crystal data of **2**.

	**2**	**1** after heating
	Single crystal data (100 K)	Le Bail fit data (298 K)
Space Group	$P2_12_12_1$	
a	17.0752(3) Å	17.0491(17) Å
b	17.1948(3) Å	17.2992(19) Å
c	38.0274(7) Å	40.0513(24) Å
α	90°	90°
β	90°	90°
γ	90°	90°
V	11165(1) Å^3	11812.56(6) Å^3

DSC analysis was done on a microcrystalline sample of **1** (10 deg/min, Figure S30). A melting point is not observed. The analysis shows two exotherms, the first broad event (113–154 °C, peak 133.5 °C) is likely reorganization of the sample in the solid state from a meta-stable polymorph. This is important, in that dimerization is not possible in the sample based on the crystallographic analysis (Figure S31), so reorganization is necessary for a reaction to occur. The second, more intense (183–238 °C, peak 196.2 °C) event clearly indicates thermal reactions. The broadness of the exotherm suggests a lack of selectivity during the thermal reaction and/or a multistep process. The lack of selectivity is confirmed through analysis of the sample before and after DSC by MALDI MS (MALDI HRMS (DCTB) and TLC analysis (Figures S32 and S33). MS shows that monomer **1** present after the DSC (*m/z* calcd for $C_{61}H_{84}$ [M^+] 816. 6568, found 816.6571) but less than ca. 5% of the sample. The major products are the expected dimer **2** (*m/z* calcd for $C_{122}H_{168}$ [M^+] 1633.3141, found 1634.3146) and "baseline" material, in equal amounts. MS shows some oxidized species, as well, but it is unclear when the oxidation occurs (the DSC is run under N_2, albeit, ambient O_2 could still be present). From prior solution state experiments, the crystallographic data of an oxidized dimer was obtained (unpublished results, access upon request), and the formula was an excellent match for the MALDI MS signal observed at *m*/*z* 1666.3076 (calcd for $C1_{22}H_{168}O_2$: *m*/*z* 1666.3078).

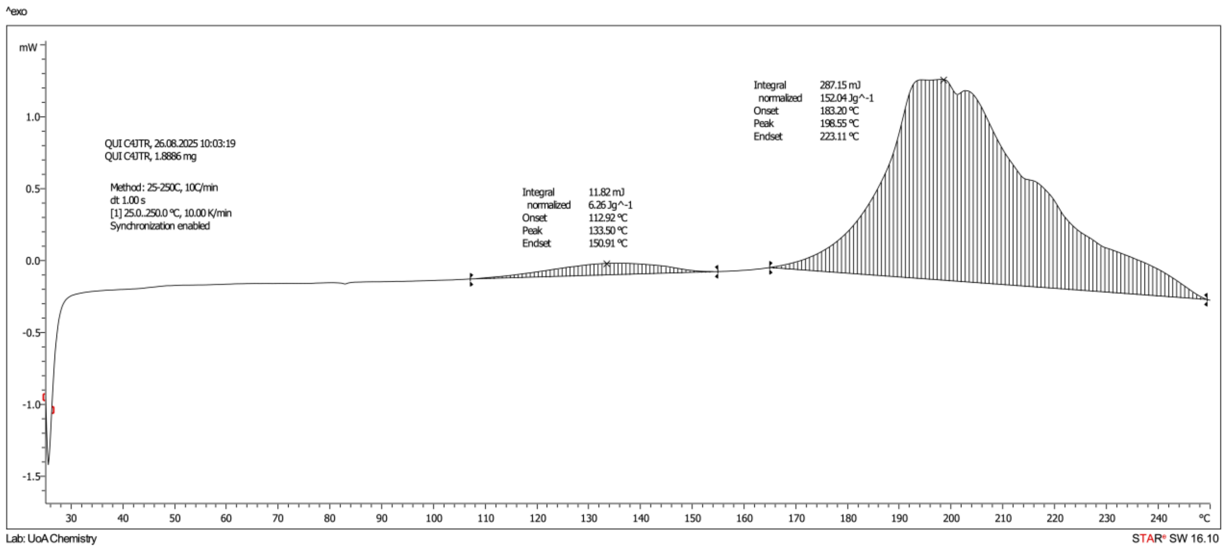

Figure S31. DSC analysis of **1** (10 deg/min).

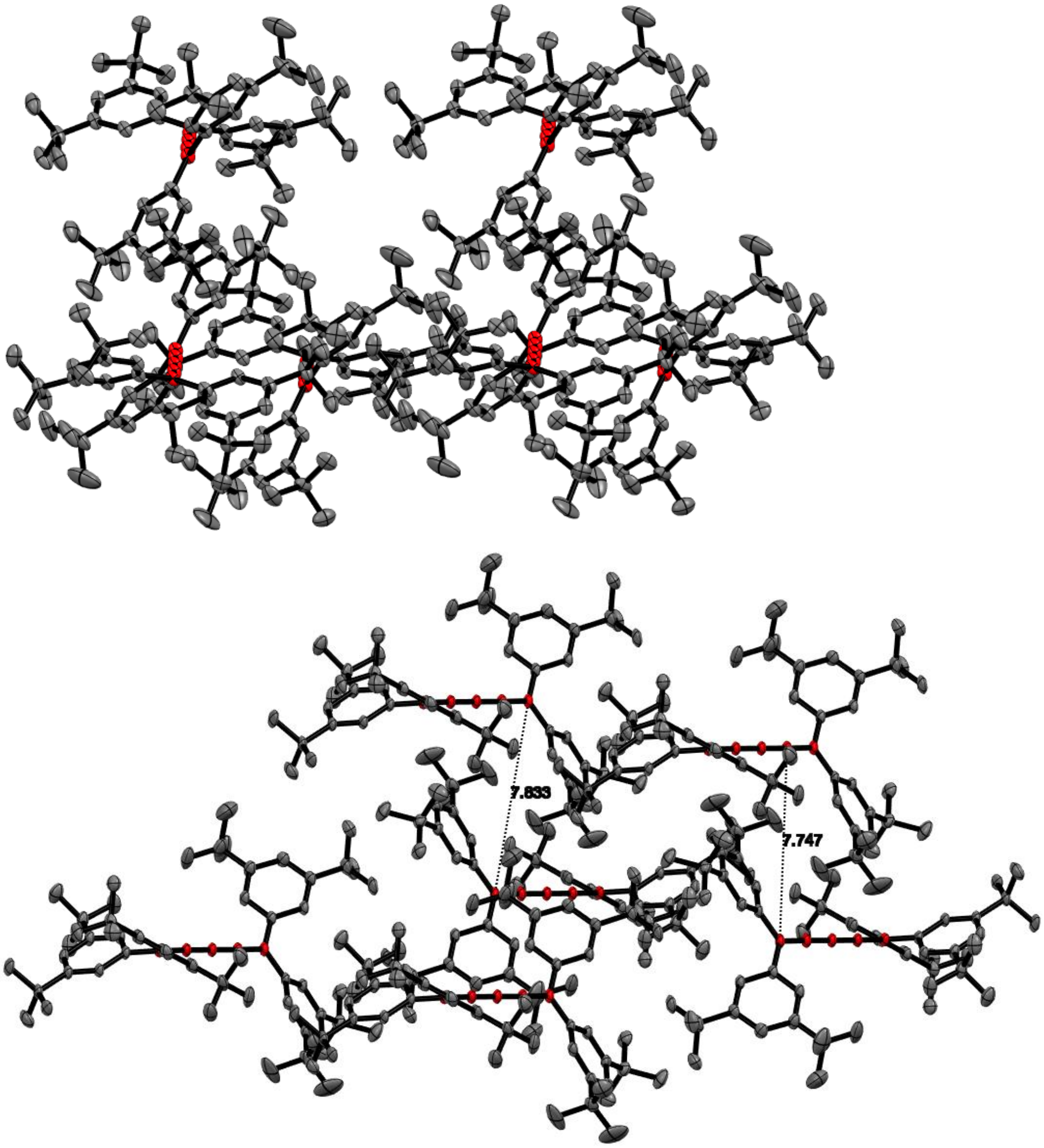

Figure S32. Packing of **1** based on X-ray crystallography before heating (CCDC 2300762); end-on view (top) and side view (bottom). Closest interatomic distances between sp-hybridized carbon atoms (shown in red) are all >7.5 Å.

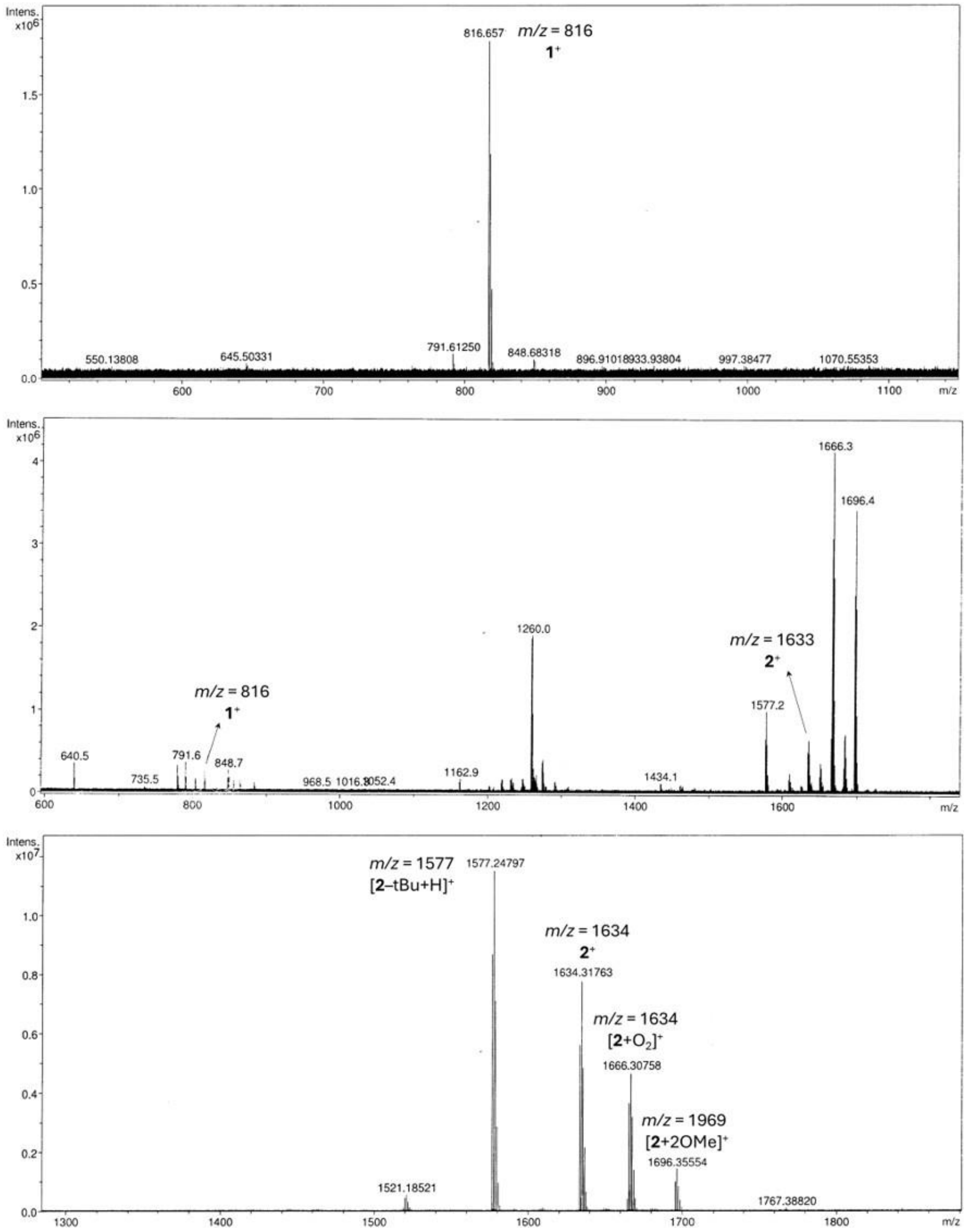

Figure S33. MALDI HRMS (DCTB) of **1** before DSC analysis (top) and after DSC analysis (middle), expansion of high-mass region after DSC analysis (bottom).

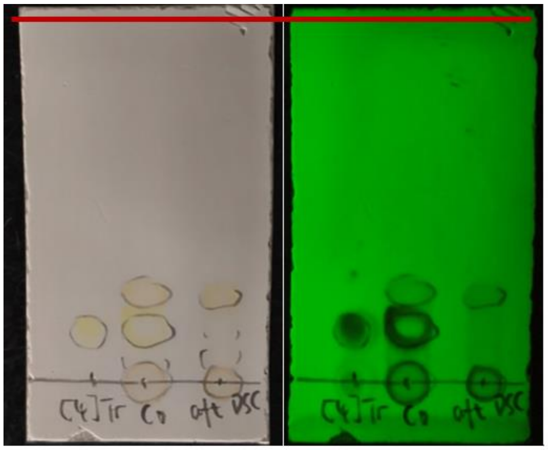

Figure S34. Thin layer chromatographic analysis (SiO_2, hexanes) of **1** before and after DSC analysis (left under ambient light and right under UV irradiation at 235 nm). In each case, the left lane is before DSC, the right lane is after DSC analysis, and the center lane is a co-spot of the two samples. Presumed dimer **2** at $R_f = 0.25$ (~40%), **1** at $R_f = 0.14$ (~5%), baseline (~50%).

VII. Crystal Structure Solution and Refinement Details

All data collections were performed on a Bruker D8 VENTURE dual source X-ray diffractometer with a PHOTON 100 CMOS detector at 100(2) K with 0.5° ϕ scan strategy. Data collections of **2** and **4** were performed using a Mo-target X-ray fine-focus sealed tube (λ = 0.71073 Å), while data collections of **3** and **5** were performed using a Cu-target Incoatec microfocus source IμS X-ray tube (λ = 1.54178 Å) as the light sources. Data reduction and integration were performed with the Bruker software package SAINT (version 8.37A).[3] Data were corrected for absorption effects using the empirical methods as implemented in SADABS (version 2016/2).[4] The structures were solved by SHELXT (version 2018/2)[5] and refined by full-matrix least-squares procedures using the Bruker SHELXTL (version 2018/3)[6] software package. All non-hydrogen atoms (including those in disordered parts) were refined anisotropically. The H-atoms were included at calculated positions and refined as riders, with U_{iso}(H) = 1.2 U_{eq}(C) and U_{iso}(H) = 1.5 U_{eq}(C) for methyl groups. In **2**, two *tert*-butyl groups were found to be disordered. In **3**, one 3,5-di-*tert*-butylphenyl group, six *tert*-butyl groups, and two 18-crown-6 molecules were found to be disordered. In **4**, three 3,5-di-*tert*-butylphenyl groups, two *tert*-butyl groups, one 18-crown-6 molecule, and one THF molecule were found to be disordered. In **5**, five *tert*-butyl groups and the 18-crown-6 molecule were found to be disordered. All disorders were modeled with two orientations. The anisotropic displacement parameters of the whole molecule in the direction of the bonds were restrained to be equal with a standard uncertainty of 0.004 Å^2. They were also restrained to have the same U_{ij} components, with a standard uncertainty of 0.01 Å^2. In each unit cell of **3**, eight *n*-hexane solvent molecules were found to be severely disordered and thus removed by the OLEX2's solvent mask.[6-7] The total void volume was 2504 Å^3, equivalent to 15.14% of the unit cell's total volume. In **4**, eight THF solvent molecules were found to be severely disordered and thus removed by the OLEX2's solvent mask.[6-7] The total void volume was 2007.6 Å^3, equivalent to 12.26% of the unit cell's total volume. In **5**, eight *n*-hexane solvent molecules were found to be severely disordered and thus removed by the OLEX2's solvent mask.[6-7] The total void volume was 2759.2 Å^3, equivalent to 16.75% of the unit cell's total volume. Further crystal and data collection details are listed in Table S5.

Table S5. Crystal data and structure refinement parameters for **2–5**.

Compound	**2**	**3**	**4**	**5**
Empirical formula	$C_{122}H_{168}$	$C_{164}H_{258}Cs_2O_{12}$	$C_{162}H_{248}Cs_2O_{16}$	$C_{164}H_{258}Rb_2O_{12}$
Formula weight	1634.55	2687.51	2717.41	2592.63
Temperature (K)	100(2)	100(2)	100(2)	100(2)
Wavelength (Å)	1.54178	0.71073	0.71073	1.54178
Crystal system	Orthorhombic	Monoclinic	Monoclinic	Monoclinic
Space group	$P2_12_12_1$	$C2/c$	$C2/c$	$C2/c$
a (Å)	17.0752(3)	31.909(3)	31.600(4)	31.6117(6)
b (Å)	17.1948(3)	21.5409(18)	21.628(3)	21.5353(4)
c (Å)	38.0274(7)	24.757(2)	24.618(3)	24.8472(5)
α (°)	90.00	90.00	90.00	90.00
β (°)	90.00	103.6500(10)	103.327(3)	103.1360(10)
γ (°)	90.00	90.00	90.00	90.00
V (Å^3)	11165.0(3)	16536(2)	16372(3)	16472.5(6)
Z	4	4	4	4
ρ_{calcd} (g·cm^{-3})	0.972	1.080	1.102	1.045
μ (mm^{-1})	0.397	0.493	0.501	1.169
F(000)	3600	5792	5832	5648
Crystal size (mm)	0.08×0.09×0.34	0.06×0.09×0.18	0.03×0.05×0.23	0.16×0.19×0.32
θ range for data collection (°)	2.82–74.29	2.91–25.11	2.80–25.08	2.90-72.40
Reflections collected	145300	135999	86364	55094
Independent reflections	22419 [R_{int} = 0.0714]	14685 [R_{int} = 0.1617]	14468 [R_{int} = 0.1785]	16107 [R_{int} = 0.0358]
Transmission factors (min/max)	0.5248/0.7196	0.6132/0.6711	0.5476/0.6260	0.6063/0.7536
Data/restraints/params.	22419/21/1209	14685/3052/1289	14468/3396/1368	16107/1506/1079
$R1$,[a] $wR2$[b] ($I > 2\sigma(I)$)	0.0541, 0.1340	0.1114, 0.2744	0.1560, 0.2925	0.1848, 0.4518
$R1$,[a] $wR2$[b] (all data)	0.0645, 0.1425	0.1857, 0.3214	0.2582, 0.3452	0.2066, 0.4856
Quality-of-fit[c]	1.016	1.029	1.069	1.814

[a] *$R1 = \Sigma||F_o|-|F_c||/\Sigma|F_o|$.* [b] *$wR2 = [\Sigma[w(F_o^2-F_c^2)^2]/\Sigma[w(F_o^2)^2]]$.*
[c] *Quality-of-fit = $[\Sigma[w(F_o^2-F_c^2)^2]/(N_{obs}-N_{params})]^{½}$, based on all data.*

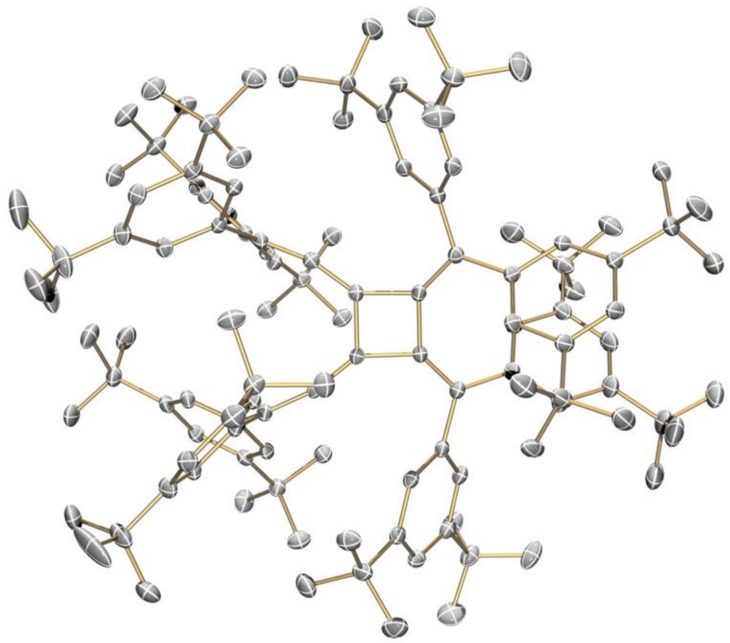

Figure S35. ORTEP drawing of the asymmetric unit of **2** with thermal ellipsoids at the 40% probability level. H-atoms are omitted for clarity. Color scheme used: C grey.

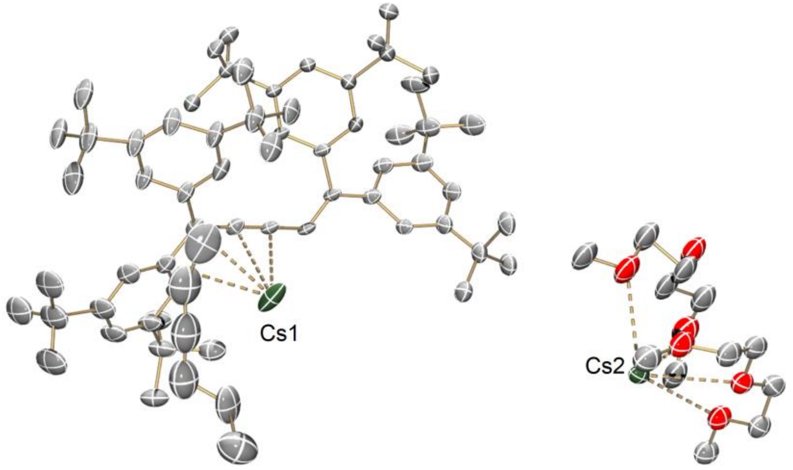

Figure S36. ORTEP drawing of the asymmetric unit of **3** with thermal ellipsoids at the 40% probability level. H-atoms are omitted for clarity. Color scheme used: Cs dark-green, O red, C grey.

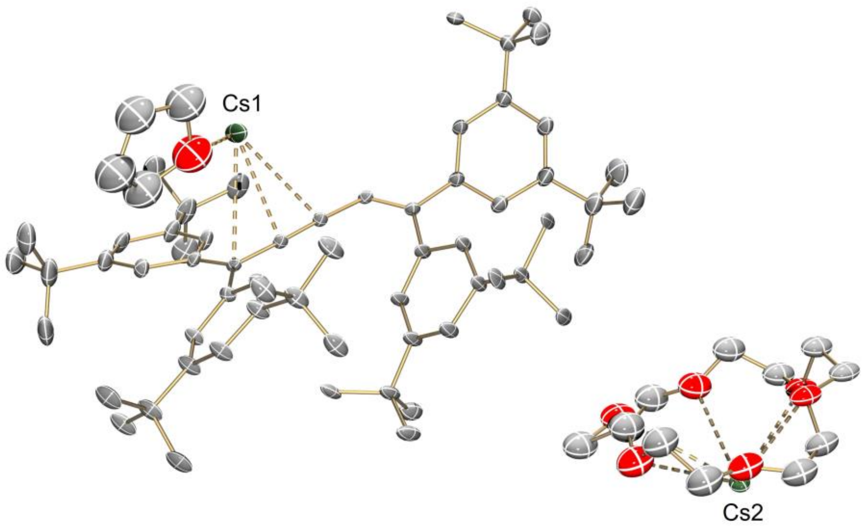

Figure S37. ORTEP drawing of the asymmetric unit of **4** with thermal ellipsoids at the 40% probability level. H-atoms are omitted for clarity. Color scheme used: Cs dark-green, O red, C grey.

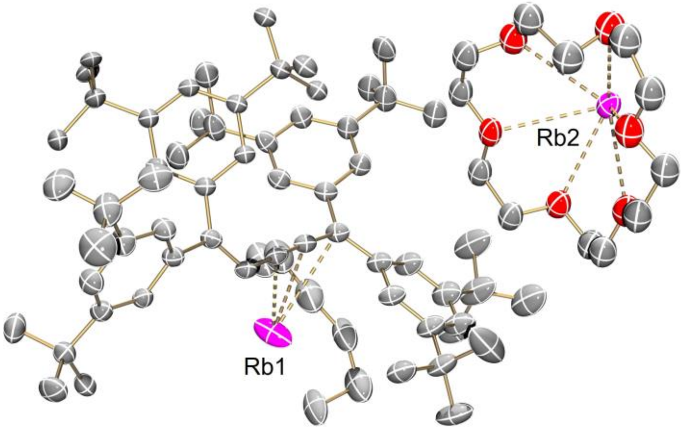

Figure S38. ORTEP drawing of the asymmetric unit of **5** with thermal ellipsoids at the 40% probability level. H-atoms are omitted for clarity. Color scheme used: Rb magenta, O red, C grey.

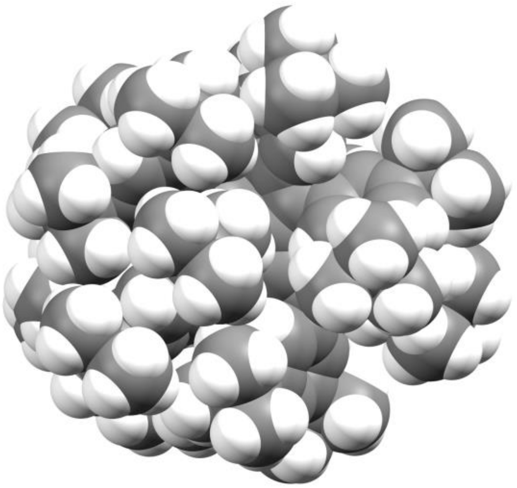

Figure S39. Crystal structure of **2**, space-filling model.

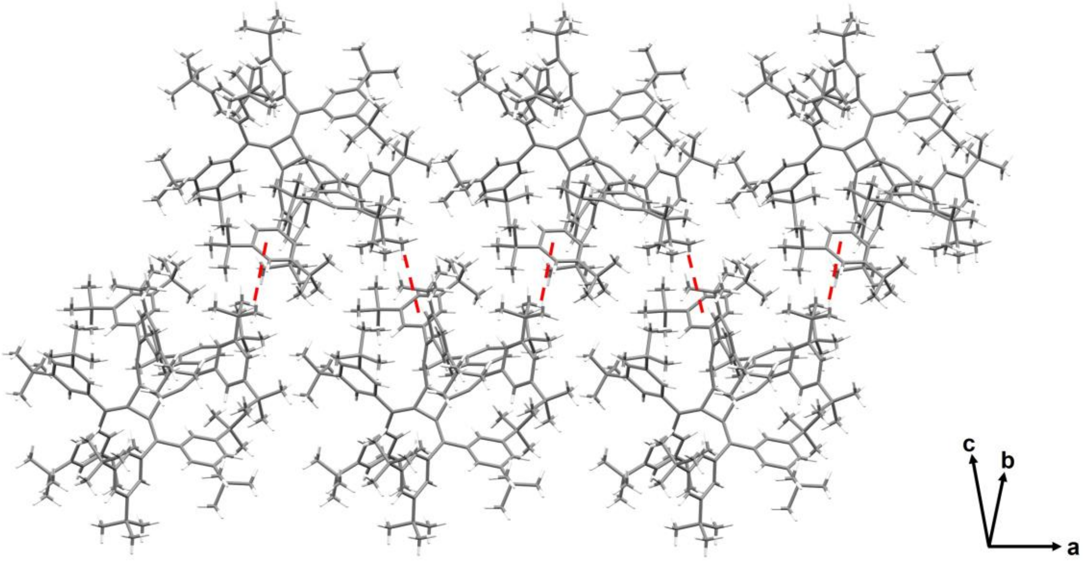

Figure S40. Zigzag columnar packing of **2**, capped-stick model. C–H···π interactions are shown in red dash lines.

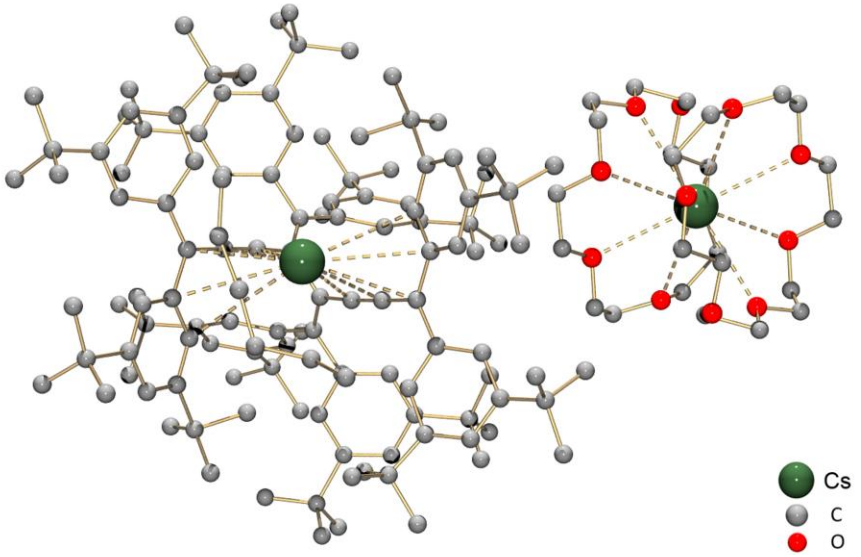

Figure S41. Crystal structure of **3**, ball-and-stick model. H-atoms are omitted for clarity.

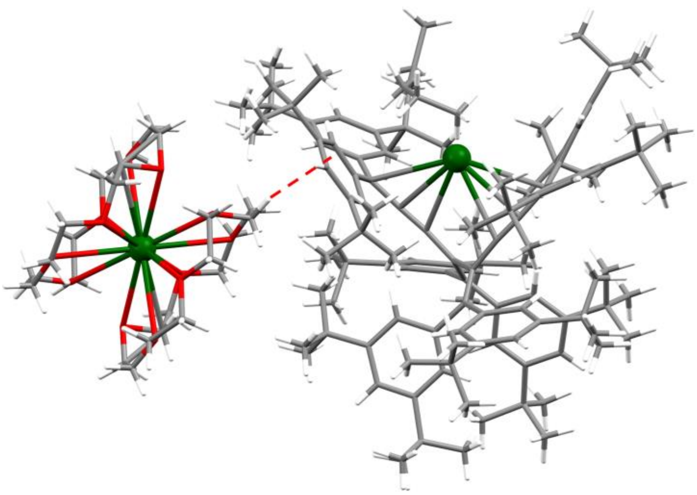

Figure S42. C–H$\cdots\pi$ interactions in **3**, mixed model.

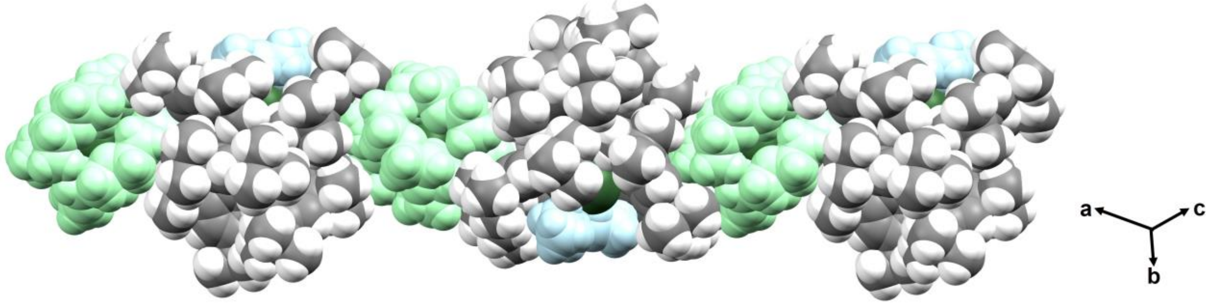

Figure S43. 1D column of **3**, space-filling model. Hexane molecules are shown in light blue, and [Cs^+(18-crown-6)$_2$] moieties are shown in light green.

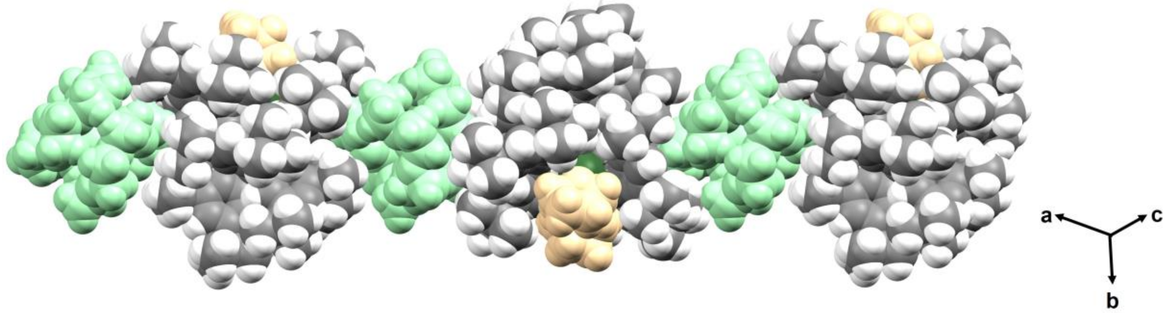

Figure S44. 1D column of **4**, space-filling model. THF molecules are shown in light orange, and [Cs^+(18-crown-6)$_2$] moieties are shown in light green.

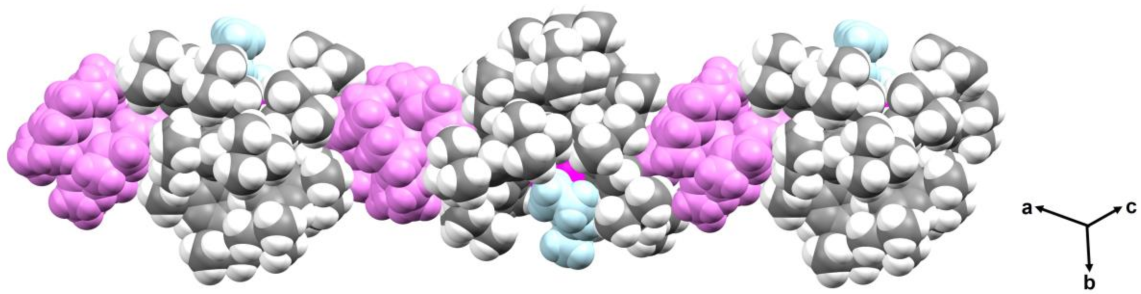

Figure S45. 1D column of **5**, space-filling model. Hexane molecules are shown in light blue, and [Rb^+(18-crown-6)$_2$] moieties are shown in pink.

Table S6. Selected C–C bond distances (Å) in **2**, along with C-atom labeling scheme.

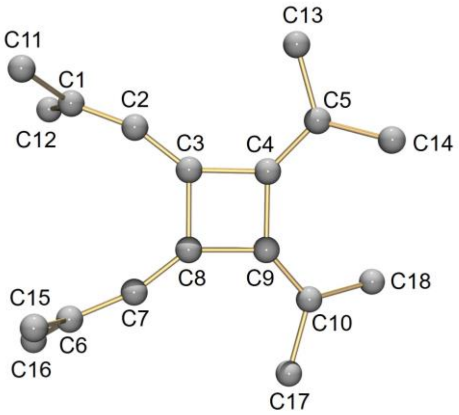

Bond	Distance	Bond	Distance
C1–C2	1.317(5)	C1–C11	1.496(5)
C2–C3	1.305(5)	C1–C12	1.488(5)
C3–C4	1.493(5)	C5–C13	1.483(5)
C3–C8	1.519(5)	C5–C14	1.485(5)
C4–C5	1.353(5)	C6–C15	1.495(5)
C4–C9	1.499(5)	C6–C16	1.491(5)
C6–C7	1.317(5)	C10–C17	1.481(5)
C7–C8	1.304(5)	C10–C18	1.480(5)
C8–C9	1.493(5)		
C9–C10	1.358(5)		

Table S7. Selected C–C bond distances (Å) in $\mathbf{2}_{\mathbf{TR}}^{2-}$, along with C-atom labeling scheme.

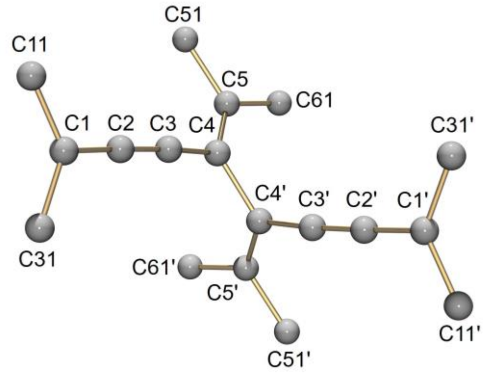

Bond	Distance	Bond	Distance
C1–C2	1.395(10)	C1–C11	1.472(10)
C2–C3	1.232(9)	C1–C31	1.484(10)
C3–C4	1.389(9)	C5–C51	1.504(10)
C4–C4′	1.539(12)	C5–C61	1.409(10)
C4–C5	1.373(9)		

Table S8. Comparison of selected angles (°) in **1**, **2**, and $\mathbf{2}_{\mathbf{TR}}^{2-}$, along with the labeling schemes.

Angle	**1**	**2**	$\mathbf{2}_{\mathbf{TR}}^{2-}$
	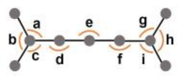	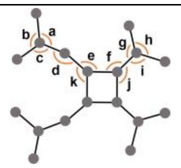	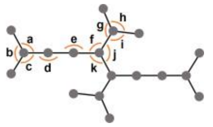
a	120.5(2)	122.2(3)	117.3(8)
b	120.9(1)	119.4(3)	125.6(6)
c	115.6(2)	118.5(3)	117.1(6)
d	178.9(3)	161.6(3)	177.7(8)
e	179.6(3)	142.5(3)	174.5(8)
f	178.8(3)	131.4(3)	125.6(6)
g	119.9(2)	120.3(3)	122.6(8)
h	121.3(2)	118.1(3)	110.5(8)
i	118.8(2)	120.0(3)	126.4(8)
j	–	136.7(3)	121.0(5)
k	–	127.9(3)	113.4(5)

*Values of two halves in **2** are averaged due to small differences.

VIII. Computational Details

All geometry optimization and energy calculations were performed with ORCA 5.0.3.[11] To save the computational cost due to the large structures, the geometry optimization and energy calculation were performed using B3LYP[8-9] functional in conjunction with 6-31g(d,p)[10] basis set, in conjunction with Grimme's D3 correction with Becke−Johnson damping.[11-12] The geometry of the initial model were extracted from crystal structure data of **1–3**. The localized orbital locator for π electrons (LOL-π) and interaction region indicators (IRI)[13] were calculated in Multiwfn 3.8 (dev)[14] and plotted in VMD. Calculations of Transition State (TS) were performed using nudged elastic band method (NEB-TS).

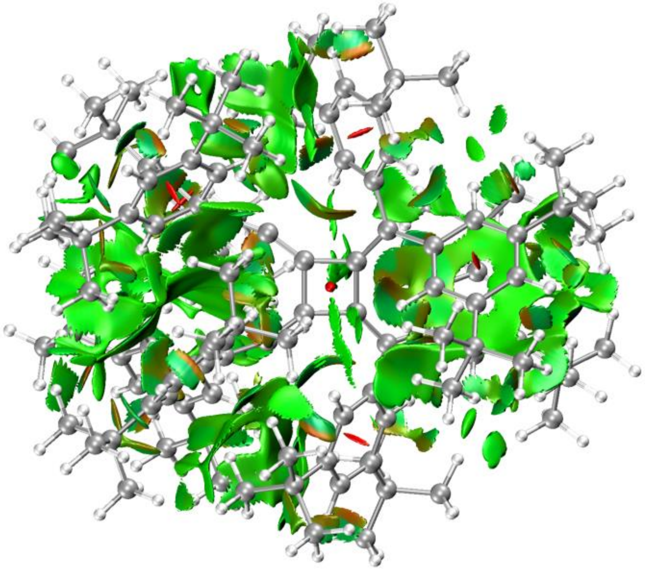

Figure S46. IRI-π isosurface plot for **2**.

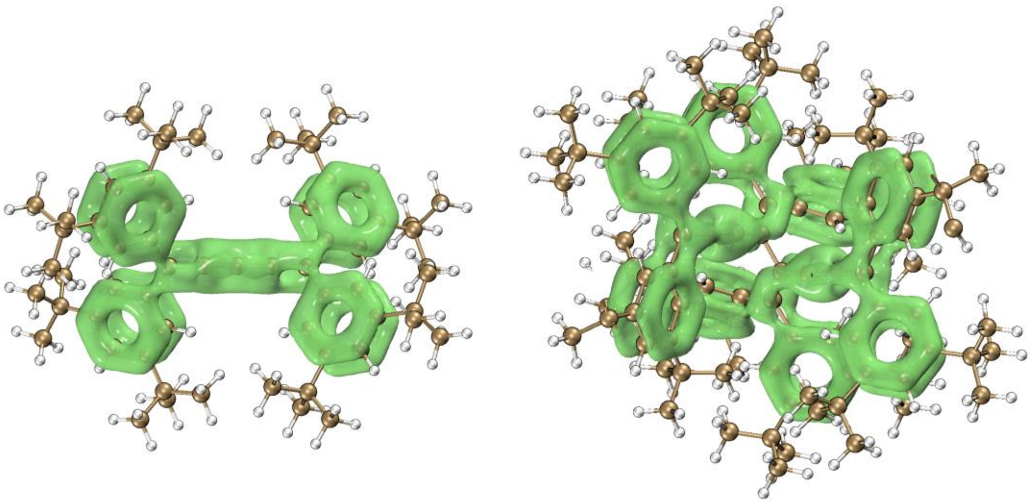

Figure S47. LOL-π isosurface plots for **1** and $\mathbf{2_{TR}}^{2-}$.

Table S9. Reaction pathways and calculated energies in this study.

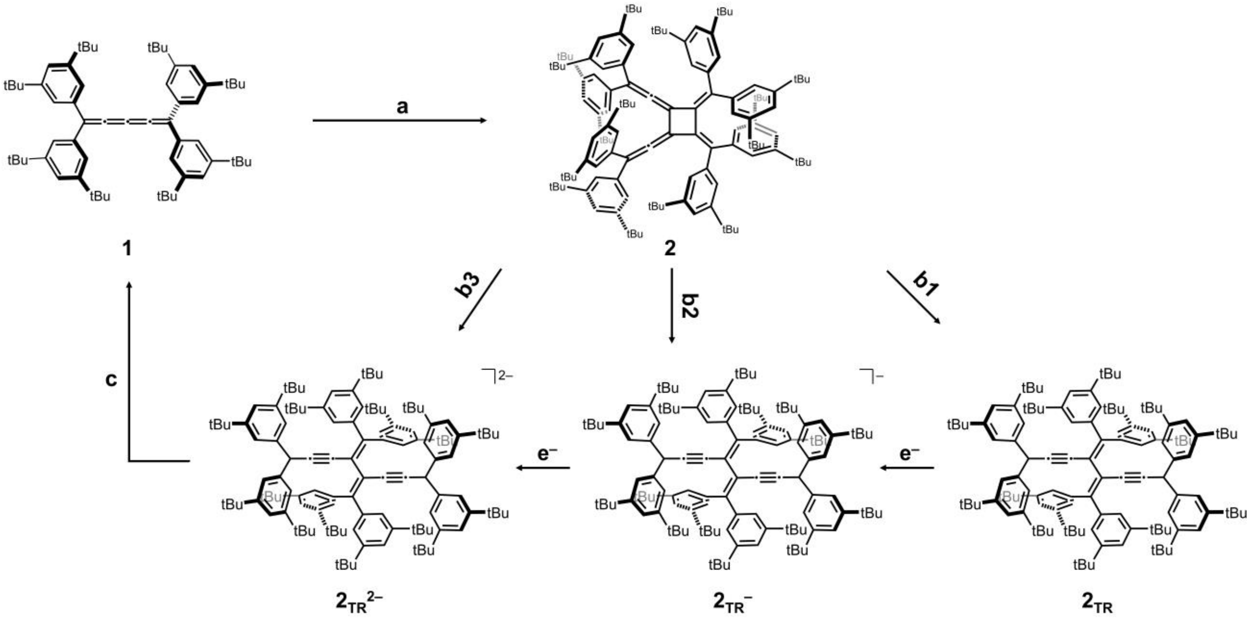

Reaction Step		Reaction Equation	ΔG
a		2[**1**] → [**2**]	+27.3 kcal/mol
Possible b	b1	[**2**] → [$\mathbf{2_{TR}}$]	+26.6 kcal/mol
	b2	[$\mathbf{2}^{-}$] → [$\mathbf{2_{TR}}^{-}$]	+6.3 kcal/mol
	b3	[$\mathbf{2}^{2-}$] → [$\mathbf{2_{TR}}^{2-}$]	–6.8 kcal/mol
c		[$\mathbf{2_{TR}}^{2-}$] + $2O_2$ → 2[**1**] + $2O_2^{-}$	–3.2 kcal/mol

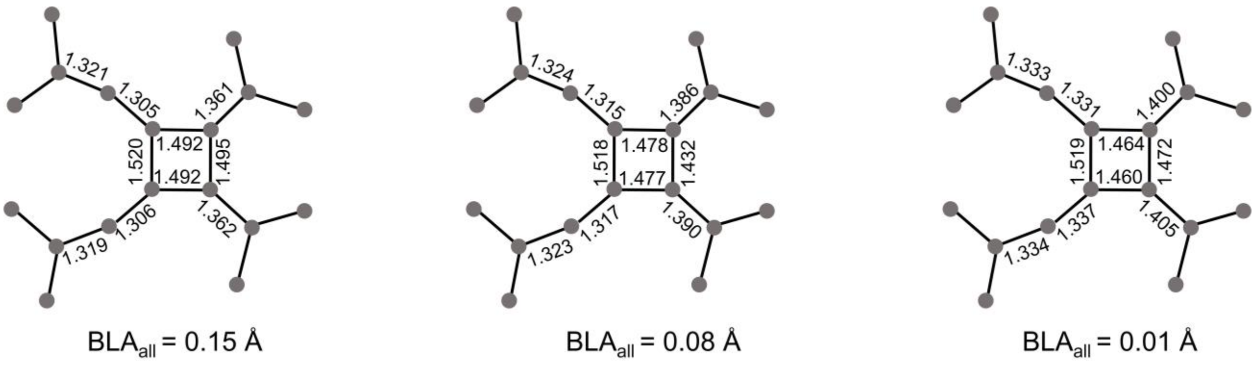

Figure S48. Comparison of C–C bond lengths in the optimized structures of **2**, $\mathbf{2}^{-}$, and $\mathbf{2}^{2-}$.

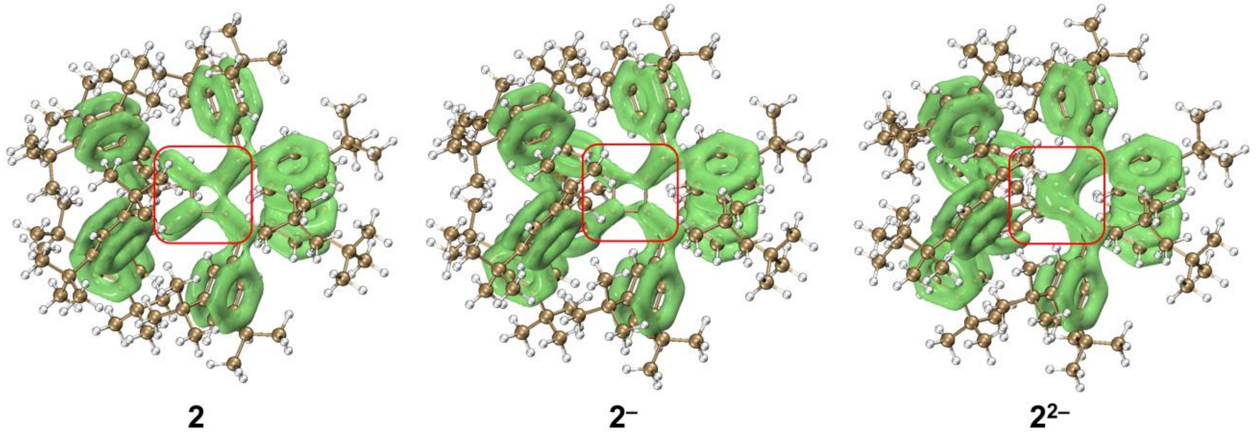

Figure S49. LOL-π isosurface plots of **2**, **2**$^{-}$, and **2**$^{2-}$.

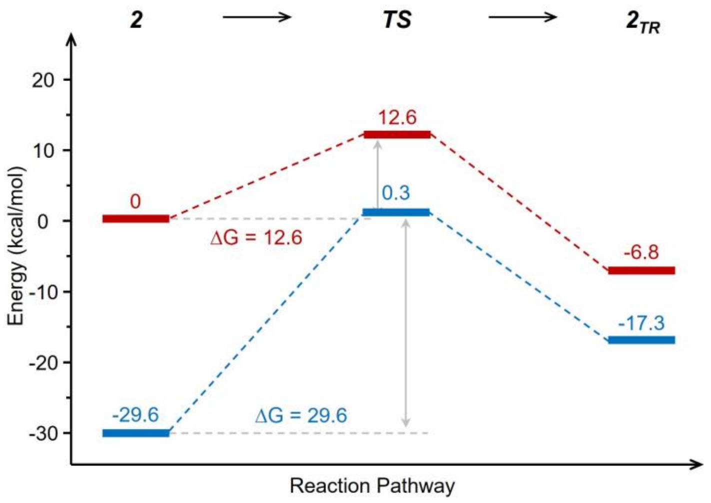

Figure S50. Transition state calculations using NEB-TS method for **2**$^{-}$ to $\mathbf{2_{TR}}^{-}$ (blue) and **2**$^{2-}$ to $\mathbf{2_{TR}}^{2-}$ (red).

Table S10. Cartesian coordinates of **1**.

Energy: -2372.32256704 Hartree, no imaginary frequence is found.

Atom	X	Y	Z	Atom	X	Y	Z
C	6.359283	7.767778	2.056283	H	12.915304	5.518218	1.841973
C	5.971358	7.025341	3.091954	H	11.527227	5.700784	0.751148
C	5.572503	6.321958	4.079591	C	3.934175	5.582662	7.157015
C	5.166962	5.619531	5.064788	C	3.999926	5.174022	8.500085
C	4.726363	4.904148	6.099091	H	4.661938	4.358789	8.758375
C	5.350748	8.211015	1.061232	C	3.234135	5.803807	9.479987
C	5.685854	8.395042	-0.282124	C	2.408720	6.875381	9.093503
H	6.702010	8.189479	-0.597582	H	1.808992	7.365415	9.850505
C	4.735914	8.833526	-1.215125	C	2.333798	7.321201	7.770864
C	3.433500	9.065857	-0.767126	C	3.102073	6.651367	6.811975
H	2.685652	9.408983	-1.471171	H	3.054872	6.945635	5.769071
C	3.055292	8.873459	0.575116	C	3.249972	5.351974	10.947870
C	4.028512	8.450914	1.475031	C	1.835685	4.868351	11.338639
H	3.794140	8.309198	2.522209	H	1.091033	5.661855	11.226267
C	5.164112	9.030806	-2.676770	H	1.526460	4.027755	10.708461
C	5.616027	7.672579	-3.257729	H	1.820001	4.537022	12.383126
H	4.796511	6.947225	-3.228595	C	4.235626	4.196290	11.191966
H	5.935291	7.788199	-4.299743	H	3.976381	3.309996	10.603230
H	6.453699	7.253653	-2.692901	H	5.263445	4.480814	10.944850
C	6.342976	10.028447	-2.732429	H	4.213500	3.910379	12.248447
H	6.050860	10.999194	-2.318306	C	3.658262	6.535490	11.852188
H	7.205046	9.667139	-2.163999	H	2.966949	7.377432	11.754663
H	6.666355	10.181501	-3.768103	H	3.665106	6.228027	12.903934
C	4.026103	9.579696	-3.552418	H	4.660159	6.893292	11.593894
H	3.666851	10.548297	-3.189195	C	1.441505	8.491100	7.329036
H	4.384038	9.720076	-4.577423	C	2.321387	9.581934	6.678144
H	3.175673	8.891113	-3.590892	H	3.062826	9.960773	7.389123
C	1.605465	9.145313	1.002903	H	2.860352	9.198152	5.807148
C	1.378300	8.873438	2.500940	H	1.702448	10.423392	6.346564
H	1.594278	7.831937	2.761307	C	0.409921	7.986901	6.294872
H	1.999012	9.518826	3.131045	H	0.895941	7.571989	5.407451
H	0.332175	9.069006	2.757483	H	-0.225101	7.206153	6.726018
C	1.256004	10.623460	0.722511	H	-0.234051	8.810947	5.967436
H	1.916906	11.291092	1.284748	C	0.678459	9.121413	8.505491
H	1.355395	10.870061	-0.338713	H	1.360029	9.518556	9.264937
H	0.222625	10.834190	1.020208	H	0.063797	9.951943	8.143900
C	0.657605	8.229920	0.196985	H	0.009832	8.400466	8.987441
H	0.884536	7.175653	0.385314	C	5.018795	3.451398	6.167225
H	-0.384271	8.413014	0.483013	C	4.158881	2.564220	6.830300
H	0.745058	8.404360	-0.879408	H	3.261958	2.958322	7.287677

C	7.789081	8.137726	1.909568	C	4.446410	1.198334	6.898350
C	8.170417	9.353969	1.324973	C	5.607529	0.736739	6.264626
H	7.396319	10.032291	0.992798	H	5.840616	-0.322119	6.310431
C	9.518948	9.689226	1.176769	C	6.483373	1.591524	5.579322
C	10.478600	8.778367	1.639072	C	6.175048	2.951241	5.548853
H	11.529303	9.022787	1.520457	H	6.828906	3.657324	5.052995
C	10.134223	7.559502	2.240943	C	3.541654	0.206629	7.645196
C	8.778585	7.254288	2.364927	C	4.357050	-0.477713	8.765183
H	8.457643	6.317725	2.802885	H	5.217653	-1.021534	8.365179
C	9.970184	10.999286	0.513750	H	4.732567	0.263389	9.478616
C	8.782318	11.893368	0.117904	H	3.731527	-1.192960	9.311040
H	8.122957	11.398927	-0.601878	C	3.023624	-0.861506	6.657518
H	8.184095	12.181024	0.988505	H	2.441372	-0.397300	5.854961
H	9.153734	12.811007	-0.349570	H	3.845196	-1.417012	6.195898
C	10.867616	11.791868	1.488649	H	2.380359	-1.581878	7.175367
H	11.759277	11.225869	1.773465	C	2.328986	0.897522	8.291638
H	11.199360	12.727966	1.025627	H	1.692426	1.384985	7.546458
H	10.321348	12.038077	2.404888	H	1.718626	0.155661	8.816393
C	10.768017	10.664057	-0.765974	H	2.636836	1.652313	9.023171
H	10.144837	10.109656	-1.475579	C	7.736262	1.017410	4.900883
H	11.109397	11.582909	-1.255986	C	8.589697	2.109208	4.231266
H	11.648295	10.052742	-0.545974	H	8.949239	2.846207	4.956637
C	11.237473	6.604634	2.720259	H	9.466239	1.653804	3.759264
C	10.665590	5.320319	3.346264	H	8.032500	2.640983	3.453265
H	10.039685	5.537016	4.217937	C	7.303078	0.007462	3.815097
H	10.065646	4.750846	2.629185	H	6.722197	-0.817308	4.238698
H	11.485738	4.675228	3.677483	H	6.683767	0.496730	3.056413
C	12.101378	7.318500	3.782802	H	8.180835	-0.420539	3.317697
H	12.567748	8.223793	3.382900	C	8.610218	0.300136	5.952614
H	11.493453	7.608816	4.645796	H	8.072165	-0.519041	6.438378
H	12.900972	6.656278	4.133810	H	9.505636	-0.120870	5.481619
C	12.123064	6.203403	1.519894	H	8.930487	0.998533	6.732530
H	12.599500	7.073267	1.058153				

Table S11. Cartesian coordinates of **2**.

Energy: -4744.60179771 Hartree, no imaginary frequence is found.

Atom	X	Y	Z	Atom	X	Y	Z
C	10.224138	10.011211	2.573616	C	-1.840579	7.120057	6.327770
C	9.586190	7.906473	1.476505	H	-1.199647	7.660376	7.030182
C	3.838931	8.424102	3.392580	H	-1.271940	6.269969	5.941474
C	4.588303	10.996530	5.507022	H	-2.699528	6.731864	6.886941
C	4.933824	5.129469	2.659587	C	-2.426069	13.008312	7.191197
H	5.865365	5.491644	2.238963	H	-2.294120	12.477036	6.244068

C	1.970539	10.653243	5.273561	H	-3.323000	13.632519	7.106597
C	-0.167428	9.365933	5.134927	H	-2.603140	12.260433	7.971478
H	-0.291075	9.532611	6.199073	C	1.114997	16.308491	3.351811
C	1.107962	9.729255	3.114183	H	1.720628	17.208522	3.499996
H	1.950098	10.200545	2.626824	H	0.363189	16.265931	4.145749
C	3.492181	15.533678	6.864815	H	0.589086	16.418218	2.397939
C	0.447545	12.275372	6.447170	C	11.267379	5.216400	5.750460
H	-0.108898	12.380460	5.522926	H	11.442763	6.261938	6.023514
C	3.287606	10.490382	4.971936	H	11.071128	5.173298	4.673975
C	4.228088	13.395841	5.955918	H	12.191189	4.662111	5.944967
C	5.142026	5.018307	7.543992	C	10.464400	4.710650	8.062803
C	7.054563	11.153131	7.119911	H	9.697914	4.246248	8.690252
H	6.444878	10.281040	7.310758	H	10.550323	5.760529	8.362772
C	8.782022	5.323653	6.312023	H	11.418753	4.214185	8.272366
C	3.903455	6.047052	2.880363	C	5.961403	2.827529	2.750759
C	2.264254	11.256521	7.665392	C	10.778754	14.707483	6.963171
H	3.100746	10.572593	7.665009	H	11.283000	15.665633	6.801094
C	6.470625	9.269603	4.780234	H	11.339885	13.940676	6.419093
C	7.475946	7.118342	5.316108	H	10.835607	14.480290	8.032775
C	1.141964	3.820902	4.323549	C	5.441146	5.000170	9.059171
C	7.214154	13.413043	6.278399	H	4.560768	4.659276	9.615498
H	6.743219	14.262321	5.796919	H	5.704745	6.001990	9.414224
C	3.917533	5.919350	7.307064	H	6.270011	4.329311	9.303431
H	3.628128	5.944584	6.252853	C	0.204019	8.966979	2.377295
H	4.105029	6.947074	7.635802	C	4.430156	6.967127	0.078265
H	3.064435	5.538208	7.876892	H	3.954208	6.008688	0.248328
C	5.298854	9.831949	4.903977	C	0.046831	12.971520	7.597534
C	4.004559	9.417956	4.222689	C	1.523613	9.557075	0.273086
C	8.692289	6.447608	5.485631	H	1.350484	10.631136	0.402506
H	9.568313	6.834242	4.983232	H	1.604198	9.356705	-0.800174
C	-0.886348	8.396893	3.059893	H	2.486757	9.306092	0.719864
H	-1.590598	7.796693	2.497885	C	2.629438	11.701612	10.158077
C	8.436545	8.641320	3.488886	C	-2.317178	8.026483	5.172398
C	5.227421	9.067929	0.958436	C	-1.193364	13.874674	7.533750
H	5.385608	9.711397	1.816018	C	8.554808	13.484648	6.677354
C	0.950053	9.919140	4.499751	C	0.968274	4.433135	5.729946
C	4.233416	14.356322	6.980387	H	1.028497	5.524742	5.702242
H	4.838857	14.167983	7.857852	H	1.746016	4.071641	6.408219
C	11.091535	11.276256	2.664642	H	-0.006066	4.155498	6.148201
C	2.700615	5.614537	3.444618	C	4.803042	3.590148	7.064535
H	1.922363	6.350445	3.617542	H	4.565498	3.587690	5.997065
C	6.379543	5.526936	6.793146	H	3.936434	3.199071	7.610679
C	7.617492	4.886198	6.956581	H	5.639287	2.903056	7.229786
H	7.676139	4.021864	7.610370	C	8.622151	7.708167	2.466628

C	5.056656	12.170765	6.014351	H	7.990964	6.826163	2.454373
C	3.476321	16.604033	7.967016	C	9.985118	3.115387	6.166818
C	10.387903	9.055419	1.560171	H	9.757401	3.018070	5.101105
H	11.157487	9.208509	0.816934	H	9.190043	2.612709	6.725111
C	3.545783	3.364656	3.504234	H	10.922744	2.584433	6.366665
H	3.401525	2.317683	3.730174	C	0.000482	4.322184	3.409643
C	2.495732	4.262887	3.747160	H	0.109770	3.927223	2.394150
C	10.232952	12.497588	2.271342	H	-0.020584	5.413443	3.345149
H	9.347887	12.581625	2.908523	H	-0.968063	3.992580	3.803114
H	9.894220	12.415046	1.234847	C	3.156446	13.051427	10.685541
H	10.814076	13.422023	2.367668	H	2.350779	13.773932	10.845516
C	9.125063	12.355024	7.274154	H	3.860329	13.485581	9.971129
H	10.162828	12.387688	7.581267	H	3.677291	12.913511	11.639609
C	0.783212	12.782952	8.769916	C	3.032042	15.188720	2.198935
H	0.481077	13.300282	9.672226	H	3.643427	14.286962	2.097038
C	1.559198	11.431971	6.463327	H	3.703484	16.028290	2.406433
C	8.390663	11.178677	7.513686	H	2.539359	15.371936	1.237229
C	3.485375	13.646349	4.799247	C	8.078045	8.839228	8.491009
H	3.530092	12.922128	3.995773	H	7.607970	8.450602	7.586277
C	6.324863	6.637017	5.952635	H	7.291155	9.168226	9.178760
H	5.394486	7.162023	5.785865	H	8.607751	8.005024	8.962296
C	1.893354	11.920327	8.828610	C	8.609991	15.915797	7.271570
C	7.411827	8.378836	4.532779	H	7.580401	16.057203	6.929233
C	9.218119	9.798344	3.524015	H	9.138173	16.868550	7.152421
H	9.038827	10.512216	4.320882	H	8.578019	15.672346	8.338731
C	5.597123	9.488776	-0.317880	C	0.373623	8.726116	0.868865
C	4.779719	3.779879	2.978998	C	5.271714	5.014629	-2.061523
C	2.729029	15.728493	5.699218	H	4.884794	4.567810	-1.141637
H	2.146192	16.636018	5.606389	H	6.351524	5.143392	-1.939514
C	4.823548	7.339738	-1.212139	H	5.100984	4.302980	-2.877232
C	9.070608	9.975398	8.187340	C	-3.229471	7.195152	4.255106
C	-1.100173	8.595944	4.428198	H	-2.701663	6.333935	3.832444
C	5.389979	8.608523	-1.391077	H	-3.627232	7.793712	3.429230
H	5.680933	8.917091	-2.384959	H	-4.080261	6.814975	4.829616
C	6.516415	11.215853	-1.956788	C	9.977564	5.477297	0.950691
H	5.627337	11.169659	-2.594482	H	9.168147	5.158293	1.613365
H	7.265518	10.527490	-2.362373	H	10.906836	5.472504	1.530532
H	6.924633	12.229054	-2.028974	H	10.067304	4.730406	0.153894
C	1.028860	2.292282	4.445504	C	0.676596	7.229164	0.633938
H	1.778368	1.883580	5.130868	H	0.807656	7.031770	-0.435642
H	1.144822	1.798581	3.474992	H	-0.135932	6.592821	0.998513
H	0.042799	2.027335	4.840434	H	1.593298	6.932020	1.148285
C	9.323493	14.798754	6.475492	C	-1.463667	14.598155	8.863460
C	7.478836	11.016182	0.341115	H	-2.352893	15.228950	8.764252

H	8.235333	10.301295	0.005350	H	-0.627837	15.243431	9.150871
H	7.306171	10.827644	1.403644	H	-1.649538	13.891587	9.678894
H	7.892767	12.025505	0.244362	C	2.099810	16.565350	8.666654
C	4.113648	7.471360	2.520347	H	1.900985	15.566154	9.065287
C	6.454626	12.255100	6.479926	H	1.295080	16.809329	7.966132
C	9.339419	15.171171	4.977601	H	2.060683	17.286161	9.491715
H	9.859202	14.406794	4.394161	C	9.714823	10.418734	9.518767
H	9.855906	16.126119	4.827978	H	8.959363	10.825654	10.198967
H	8.328301	15.271688	4.572816	H	10.481521	11.185043	9.370851
C	2.720368	14.805250	4.649816	H	10.191439	9.563254	10.010188
C	4.617666	7.825364	1.166925	C	-3.142403	9.199663	5.747875
C	12.312499	11.215342	1.730952	H	-3.487405	9.861267	4.946316
H	12.019200	11.173678	0.677193	H	-2.555415	9.801406	6.447668
H	12.940458	10.344826	1.947318	H	-4.021422	8.822637	6.282872
H	12.923591	12.113636	1.865416	C	4.589853	6.364795	-2.375382
C	10.113875	4.601908	6.562227	C	3.070565	6.142175	-2.545789
C	10.166161	9.428158	7.244679	H	2.562731	7.088550	-2.760195
H	10.930489	10.183113	7.035958	H	2.623545	5.720349	-1.641268
H	9.736941	9.110022	6.291686	H	2.874080	5.451864	-3.374053
H	10.660977	8.561945	7.699691	C	1.648079	11.094766	11.184773
C	-0.926336	9.105820	0.127309	H	1.261788	10.133589	10.830163
H	-1.171605	10.161319	0.285349	H	0.793558	11.755082	11.360126
H	-1.779852	8.509062	0.461834	H	2.149611	10.930003	12.145179
H	-0.807440	8.939724	-0.949135	C	5.155543	6.892006	-3.704393
C	6.185590	10.894648	-0.490442	H	6.232969	7.075205	-3.638902
C	4.576916	16.374798	9.018107	H	4.666857	7.821815	-4.012695
H	5.569703	16.341264	8.557162	H	4.989015	6.153316	-4.495041
H	4.428533	15.448338	9.577284	C	3.831546	10.751541	10.007781
H	4.568897	17.196541	9.741421	H	4.563628	11.134055	9.288679
C	3.706947	18.004284	7.357381	H	3.522829	9.752767	9.682259
H	2.926518	18.281202	6.643894	H	4.336525	10.642736	10.973082
H	4.669503	18.047515	6.837041	C	10.855154	7.207080	-0.625328
H	3.712608	18.761781	8.148796	H	11.824948	7.233178	-0.117370
C	11.601362	11.462856	4.111559	H	10.699565	8.172976	-1.117068
H	12.182643	10.594018	4.437573	H	10.909039	6.443084	-1.407651
H	10.781085	11.603490	4.819879	C	8.388009	6.844061	-0.449118
H	12.244795	12.348076	4.170871	H	8.184708	7.813059	-0.912953
C	1.069954	13.833686	3.003179	H	7.530033	6.610024	0.186577
H	0.277633	13.735524	3.749576	H	8.439549	6.088285	-1.241463
H	1.620249	12.889584	2.983536	C	6.396146	2.891743	1.269254
H	0.597191	13.966965	2.023740	H	6.716323	3.897182	0.981618
C	-0.996945	14.940690	6.433943	H	5.574613	2.599813	0.606441
H	-1.848904	15.629474	6.412464	H	7.236777	2.211978	1.090654
H	-0.913134	14.484899	5.444141	C	5.612422	1.369837	3.091516

H	-0.084217	15.516246	6.610978	H	4.784939	0.997145	2.478553
C	1.981204	15.038406	3.323695	H	5.338317	1.255751	4.145411
C	5.154715	11.931360	0.014865	H	6.480535	0.729738	2.904178
H	4.922714	11.782694	1.073662	C	7.139482	3.268106	3.647965
H	4.217446	11.856059	-0.546728	H	6.869523	3.218566	4.705776
H	5.546605	12.947998	-0.103407	H	7.449094	4.297356	3.445538
C	9.711773	6.874797	0.346991	H	8.004565	2.615350	3.483470

Table S12. Cartesian coordinates of **2**$^{-}$.

Energy: -4744.61929812 Hartree, no imaginary frequence is found.

Atom	X	Y	Z	Atom	X	Y	Z
C	10.342568	10.001025	2.541743	C	-1.885831	7.099980	6.270664
C	9.680507	7.924166	1.411463	H	-1.236450	7.622952	6.977865
C	3.801800	8.309134	3.506433	H	-1.322157	6.256325	5.862239
C	4.576186	10.998172	5.445933	H	-2.743789	6.702649	6.827156
C	4.890281	5.041522	2.661548	C	-2.413413	12.918295	7.439373
H	5.828581	5.410382	2.261099	H	-2.326000	12.380938	6.490915
C	1.945866	10.634005	5.294308	H	-3.319680	13.535160	7.397403
C	-0.213052	9.374932	5.120178	H	-2.542737	12.173593	8.232460
H	-0.351193	9.538806	6.183418	C	0.900059	16.139269	3.327789
C	1.098139	9.715521	3.124038	H	1.503095	17.050441	3.403587
H	1.956211	10.170599	2.648770	H	0.187577	16.130217	4.158644
C	3.467909	15.578508	6.742575	H	0.328189	16.195975	2.394935
C	0.422634	12.190422	6.562915	C	11.204931	5.230452	5.766971
H	-0.196238	12.266099	5.675836	H	11.389779	6.279210	6.019521
C	3.286994	10.443509	5.000027	H	11.028518	5.167230	4.688144
C	4.205970	13.400260	5.905334	H	12.116017	4.665713	5.993187
C	5.040673	5.172754	7.427124	C	10.346829	4.791221	8.071905
C	7.010922	11.137993	7.119347	H	9.558530	4.355034	8.692680
H	6.395128	10.263214	7.276492	H	10.442178	5.846559	8.348245
C	8.709280	5.390342	6.272504	H	11.288868	4.284276	8.314086
C	3.862304	5.960852	2.887003	C	5.923091	2.743754	2.748673
C	2.356192	11.270696	7.665211	C	10.777217	14.651187	7.041652
H	3.219376	10.623695	7.619281	H	11.298687	15.600991	6.878803
C	6.498395	9.367959	4.612525	H	11.334078	13.869901	6.514032
C	7.459713	7.198499	5.219845	H	10.812274	14.430190	8.113604
C	1.087261	3.725076	4.288654	C	5.295223	5.231869	8.949657
C	7.200133	13.409689	6.329196	H	4.399547	4.916523	9.497755
H	6.748379	14.271562	5.850689	H	5.544874	6.252330	9.258586
C	3.825855	6.062216	7.109571	H	6.120704	4.579372	9.250955
H	3.581376	6.055925	6.044212	C	0.198657	8.961481	2.376089
H	3.997568	7.100641	7.410001	C	4.448064	6.971435	0.124557
H	2.950189	5.697754	7.656383	H	3.977556	6.002811	0.249101

C	5.299742	9.867877	4.830187	C	0.083132	12.902685	7.720393
C	3.983506	9.371454	4.259231	C	1.546033	9.552057	0.286955
C	8.656959	6.501248	5.425655	H	1.377490	10.625945	0.422837
H	9.551077	6.864465	4.937216	H	1.637538	9.356710	-0.787169
C	-0.910645	8.397872	3.036268	H	2.501510	9.294178	0.744498
H	-1.610383	7.801370	2.464324	C	2.889456	11.792919	10.109100
C	8.496197	8.668706	3.403185	C	-2.359381	8.035634	5.137208
C	5.185746	9.052416	1.087711	C	-1.169106	13.792639	7.709026
H	5.303600	9.673095	1.967254	C	8.537632	13.456632	6.735802
C	0.930081	9.920178	4.512437	C	0.910853	4.333103	5.697097
C	4.241632	14.426262	6.872375	H	0.975505	5.423944	5.672320
H	4.894585	14.294896	7.726049	H	1.686435	3.968858	6.376976
C	11.225309	11.253164	2.660394	H	-0.066502	4.058278	6.111583
C	2.654476	5.516312	3.432179	C	4.712620	3.722237	7.012353
H	1.881637	6.255429	3.615738	H	4.501866	3.665169	5.940935
C	6.303590	5.642485	6.692913	H	3.830923	3.359718	7.554926
C	7.524880	4.981753	6.899001	H	5.542919	3.043075	7.233802
H	7.553281	4.130115	7.571967	C	8.688656	7.740530	2.377660
C	5.032712	12.186595	6.003980	H	8.032422	6.876966	2.340042
C	3.479366	16.698029	7.795848	C	9.882063	3.160528	6.203852
C	10.508640	9.052193	1.521227	H	9.673730	3.043472	5.136175
H	11.295849	9.195753	0.794216	H	9.066113	2.684735	6.755520
C	3.495067	3.264233	3.474800	H	10.806819	2.618339	6.435513
H	3.350416	2.215492	3.694054	C	-0.049814	4.236887	3.374755
C	2.444835	4.163042	3.718007	H	0.059776	3.845100	2.357759
C	10.392769	12.486489	2.247301	H	-0.062738	5.328415	3.313874
H	9.492418	12.576377	2.860892	H	-1.022147	3.912883	3.765078
H	10.078127	12.408222	1.202205	C	3.394352	13.174551	10.571270
H	10.980341	13.405409	2.362018	H	2.571043	13.865305	10.776616
C	9.098432	12.314421	7.319539	H	4.019700	13.619287	9.793971
H	10.134272	12.328111	7.635050	H	3.993096	13.081501	11.485276
C	0.905753	12.776351	8.844677	C	2.764641	14.971091	2.140270
H	0.658007	13.314729	9.751542	H	3.378120	14.069259	2.058934
C	1.566224	11.383693	6.503652	H	3.439687	15.824535	2.266221
C	8.340998	11.144861	7.527972	H	2.222049	15.096990	1.195228
C	3.381292	13.585987	4.785587	C	7.996605	8.787948	8.456995
H	3.388370	12.819861	4.021243	H	7.542737	8.421145	7.535744
C	6.287175	6.741776	5.837174	H	7.196585	9.112413	9.131570
H	5.371450	7.281988	5.642142	H	8.510829	7.941588	8.925463
C	2.045986	11.951251	8.835032	C	8.627396	15.900528	7.296401
C	7.430417	8.449484	4.416852	H	7.606261	16.053494	6.935834
C	9.310867	9.802068	3.465502	H	9.174710	16.843053	7.172533
H	9.120978	10.515507	4.260334	H	8.572213	15.672608	8.366194
C	5.604748	9.509556	-0.159745	C	0.386006	8.724937	0.867998

C	4.732681	3.688252	2.964640	C	5.333068	5.077344	-2.046999
C	2.629896	15.703557	5.617704	H	4.913077	4.610575	-1.152055
H	2.018669	16.591817	5.516684	H	6.410388	5.186353	-1.889327
C	4.879991	7.385728	-1.140864	H	5.176578	4.391560	-2.888359
C	8.999738	9.924603	8.193601	C	-3.282936	7.230746	4.207491
C	-1.137970	8.609790	4.401530	H	-2.765365	6.370693	3.769898
C	5.445942	8.661370	-1.266882	H	-3.669615	7.848439	3.390222
H	5.773043	8.999853	-2.239980	H	-4.139791	6.851620	4.775422
C	6.658268	11.265381	-1.684142	C	10.047518	5.495045	0.866905
H	5.836460	11.230446	-2.407743	H	9.231343	5.186359	1.525994
H	7.441847	10.578279	-2.022297	H	10.974351	5.473436	1.450722
H	7.073754	12.278495	-1.701838	H	10.128555	4.750891	0.065703
C	0.966130	2.196570	4.403907	C	0.682804	7.227899	0.626652
H	1.712805	1.782119	5.089243	H	0.829039	7.034276	-0.442644
H	1.084170	1.706725	3.431316	H	-0.139399	6.595483	0.977226
H	-0.022717	1.933955	4.794899	H	1.589779	6.923530	1.153543
C	9.330581	14.756989	6.530814	C	-1.377986	14.533502	9.040276
C	7.384025	11.066596	0.698257	H	-2.281088	15.151163	8.981643
H	8.181763	10.362761	0.443879	H	-0.536042	15.193989	9.269929
H	7.104050	10.877545	1.737058	H	-1.504478	13.836821	9.875669
H	7.790447	12.082495	0.643483	C	2.120479	16.698971	8.531275
C	4.069672	7.398819	2.583520	H	1.924124	15.714686	8.967249
C	6.412807	12.257335	6.494188	H	1.299787	16.925725	7.843840
C	9.378024	15.105206	5.027384	H	2.108488	17.448883	9.332270
H	9.893581	14.320627	4.466589	C	9.615157	10.339218	9.547842
H	9.912841	16.049537	4.866822	H	8.842334	10.730242	10.218139
H	8.374251	15.210433	4.606851	H	10.380496	11.112635	9.432343
C	2.586727	14.721765	4.622802	H	10.083582	9.474450	10.032990
C	4.586394	7.797129	1.246654	C	-3.176422	9.198619	5.743130
C	12.469956	11.178171	1.759503	H	-3.516526	9.881772	4.957495
H	12.203144	11.143953	0.698264	H	-2.582649	9.779702	6.453875
H	13.078071	10.296276	1.987899	H	-4.058316	8.816398	6.271790
H	13.091356	12.066797	1.913835	C	4.680986	6.446564	-2.340451
C	10.024338	4.653782	6.567480	C	3.166429	6.249034	-2.572882
C	10.114243	9.387679	7.267764	H	2.679383	7.208606	-2.776375
H	10.887835	10.142096	7.092978	H	2.683488	5.811575	-1.694826
H	9.704138	9.094291	6.298642	H	2.989053	5.583853	-3.426763
H	10.593113	8.507212	7.714592	C	2.019118	11.165704	11.219507
C	-0.902237	9.115947	0.111514	H	1.653655	10.180157	10.912198
H	-1.140998	10.172618	0.273114	H	1.148026	11.788734	11.446024
H	-1.763851	8.524866	0.435739	H	2.598676	11.044950	12.143019
H	-0.772975	8.954254	-0.965359	C	5.302410	7.004137	-3.631840
C	6.184925	10.925887	-0.261618	H	6.377873	7.175683	-3.517694
C	4.599536	16.517645	8.835447	H	4.835130	7.947767	-3.931176

H	5.583793	16.460756	8.359273	H	5.160302	6.289298	-4.449718
H	4.459938	15.615691	9.434787	C	4.120566	10.895501	9.884670
H	4.604687	17.371656	9.522035	H	4.772422	11.292248	9.100410
C	3.699606	18.067304	7.116160	H	3.832978	9.877019	9.604081
H	2.915754	18.298710	6.390118	H	4.705219	10.832759	10.809392
H	4.659578	18.085933	6.589101	C	10.960314	7.227256	-0.685595
H	3.705713	18.867606	7.866025	H	11.925735	7.236976	-0.168350
C	11.698189	11.431072	4.121084	H	10.820426	8.200650	-1.167253
H	12.253737	10.551172	4.462608	H	11.011573	6.470103	-1.475524
H	10.861355	11.584722	4.806636	C	8.488357	6.891749	-0.536321
H	12.355044	12.305487	4.199851	H	8.288133	7.874372	-0.972536
C	0.859617	13.650887	3.115199	H	7.624923	6.639716	0.084330
H	0.090081	13.607047	3.889750	H	8.547545	6.159421	-1.350878
H	1.410682	12.709406	3.154926	C	6.371769	2.807563	1.271465
H	0.358475	13.712762	2.141743	H	6.678991	3.817128	0.984767
C	-1.033060	14.847726	6.588858	H	5.559484	2.503657	0.602185
H	-1.881705	15.542827	6.605695	H	7.223761	2.138616	1.101947
H	-1.004170	14.379070	5.602224	C	5.582290	1.283887	3.088711
H	-0.106190	15.415469	6.706831	H	4.761532	0.905173	2.469981
C	1.775992	14.875376	3.325508	H	5.298994	1.171192	4.140360
C	5.086393	11.936285	0.145928	H	6.456683	0.648891	2.909992
H	4.754424	11.766774	1.174201	C	7.090045	3.194238	3.655909
H	4.211458	11.845828	-0.506942	H	6.804081	3.162063	4.710367
H	5.464082	12.963410	0.079092	H	7.403105	4.220549	3.444977
C	9.804033	6.900943	0.273613	H	7.957298	2.538322	3.512745

Table S13. Cartesian coordinates of $\mathbf{2}^{2-}$.

Energy: -4744.58057433 Hartree, no imaginary frequence is found.

Atom	X	Y	Z	Atom	X	Y	Z
C	10.485694	10.030275	2.531709	C	-1.986061	7.116155	6.195926
C	9.857553	7.955579	1.385309	H	-1.328834	7.609122	6.916780
C	3.768213	8.171708	3.611687	H	-1.442532	6.262163	5.782095
C	4.610157	10.993341	5.338668	H	-2.863727	6.737189	6.736645
C	4.802909	4.919901	2.645973	C	-2.341032	12.698918	7.740151
H	5.759313	5.261092	2.263289	H	-2.302795	12.167206	6.785862
C	1.970425	10.564433	5.320761	H	-3.270391	13.283496	7.766230
C	-0.229450	9.361616	5.103565	H	-2.384448	11.948736	8.537635
H	-0.381731	9.511076	6.167419	C	0.644878	15.934401	3.367592
C	1.117986	9.689477	3.133995	H	1.228417	16.861823	3.377899
H	1.998087	10.120521	2.677155	H	-0.018529	15.939187	4.238590
C	3.420097	15.556571	6.640612	H	0.018361	15.943221	2.467661
C	0.453340	12.043728	6.695802	C	11.074391	5.176272	5.766396
H	-0.231733	12.083199	5.856085	H	11.300273	6.224612	5.984019

C	3.320021	10.370287	5.001922	H	10.905631	5.084377	4.688493
C	4.204179	13.380084	5.816387	H	11.958319	4.580553	6.023408
C	4.895707	5.442227	7.349302	C	10.173873	4.857250	8.073697
C	7.031200	11.145579	7.039237	H	9.358180	4.481017	8.698366
H	6.425022	10.258440	7.161010	H	10.312668	5.916752	8.312169
C	8.580602	5.462262	6.235058	H	11.089900	4.317160	8.346999
C	3.806791	5.876005	2.870075	C	5.772390	2.595650	2.765485
C	2.505111	11.235336	7.660363	C	10.778976	14.677591	7.064490
H	3.390532	10.625775	7.563923	H	11.301062	15.630043	6.912675
C	6.576529	9.518906	4.383391	H	11.344743	13.897782	6.544337
C	7.426996	7.302980	5.114732	H	10.795386	14.451270	8.135899
C	0.954188	3.727218	4.254670	C	5.136190	5.524520	8.873357
C	7.214687	13.430860	6.298304	H	4.220315	5.265664	9.418955
H	6.769434	14.299360	5.824479	H	5.430358	6.539789	9.159318
C	3.723153	6.376184	6.999937	H	5.927752	4.841897	9.200133
H	3.499829	6.379242	5.930556	C	0.212735	8.971238	2.364238
H	3.929763	7.410092	7.293389	C	4.524692	6.946118	0.173017
H	2.823368	6.050461	7.533545	H	4.067893	5.966763	0.263190
C	5.334409	9.895683	4.703472	C	0.171187	12.762985	7.861009
C	4.004820	9.310927	4.258423	C	1.607083	9.566161	0.305190
C	8.587609	6.550168	5.358276	H	1.461538	10.640209	0.464153
H	9.504273	6.859078	4.873870	H	1.708110	9.391562	-0.772426
C	-0.925574	8.423724	2.994492	H	2.549564	9.276618	0.770272
H	-1.631581	7.853359	2.403487	C	3.184411	11.814872	10.054084
C	8.605640	8.703642	3.339161	C	-2.411562	8.083427	5.070341
C	5.183826	9.024282	1.190661	C	-1.109129	13.612273	7.920664
H	5.266384	9.635542	2.079477	C	8.546427	13.475057	6.721490
C	0.947545	9.883568	4.529590	C	0.791344	4.367327	5.650879
C	4.253131	14.446618	6.746761	H	0.874515	5.455683	5.601138
H	4.959938	14.366647	7.563353	H	1.564641	4.006376	6.335199
C	11.351559	11.292134	2.670439	H	-0.189479	4.118586	6.075256
C	2.581077	5.458860	3.401696	C	4.510132	3.998154	6.963551
H	1.838137	6.226312	3.592945	H	4.303455	3.925299	5.892570
C	6.188383	5.835728	6.620023	H	3.610716	3.684084	7.508160
C	7.373371	5.120392	6.858328	H	5.310737	3.290106	7.205750
H	7.354621	4.286564	7.554143	C	8.837113	7.772539	2.322811
C	5.054012	12.194326	5.916364	H	8.172964	6.916025	2.258102
C	3.435715	16.692993	7.676475	C	9.661172	3.182126	6.260741
C	10.684482	9.082159	1.514626	H	9.462555	3.034758	5.194987
H	11.489055	9.228494	0.806773	H	8.816582	2.763768	6.815560
C	3.349062	3.180101	3.448422	H	10.558833	2.608985	6.526860
H	3.173232	2.136922	3.673134	C	-0.163733	4.257078	3.327549
C	2.326534	4.114616	3.682882	H	-0.060686	3.844788	2.317613
C	10.511074	12.515464	2.243060	H	-0.140784	5.347147	3.248135

H	9.602900	12.595404	2.845632
H	10.207290	12.427999	1.195020
H	11.086038	13.442781	2.361124
C	9.112591	12.330926	7.297481
H	10.143657	12.343915	7.628559
C	1.077580	12.698793	8.927851
H	0.875478	13.243979	9.842119
C	1.630152	11.286478	6.549131
C	8.349050	11.156792	7.476098
C	3.292692	13.507824	4.748454
H	3.280376	12.721696	4.005121
C	6.231310	6.915322	5.741437
H	5.341716	7.489224	5.529848
C	2.248284	11.922077	8.839191
C	7.471034	8.534492	4.286815
C	9.427407	9.832344	3.422511
H	9.199847	10.553739	4.200254
C	5.634115	9.507984	-0.035850
C	4.600823	3.570891	2.947608
C	2.503066	15.621084	5.571015
H	1.845170	16.477876	5.490668
C	4.990940	7.387416	-1.070043
C	8.995754	9.932947	8.148135
C	-1.162413	8.632006	4.359313
C	5.535547	8.675570	-1.162472
H	5.889327	9.035825	-2.118521
C	6.649745	11.330277	-1.507698
H	5.828398	11.289754	-2.232464
H	7.453312	10.675790	-1.864193
H	7.035942	12.355549	-1.496838
C	0.786887	2.205319	4.396546
H	1.521092	1.781649	5.090053
H	0.894176	1.695809	3.432425
H	-0.210753	1.977869	4.789126
C	9.339905	14.778503	6.530770
C	7.386315	11.071522	0.867306
H	8.193253	10.386531	0.589580
H	7.108699	10.848089	1.900649
H	7.778369	12.095092	0.839049
C	4.056884	7.319579	2.628124
C	6.422703	12.270010	6.419517
C	9.413025	15.126286	5.028161
H	9.938814	14.340328	4.478546
H	9.948537	16.072487	4.872504

H	-1.148619	3.970289	3.717783
C	3.646247	13.222551	10.482325
H	2.803438	13.872464	10.736914
H	4.196302	13.691657	9.663566
H	4.307859	13.165818	11.356223
C	2.463112	14.768586	2.112027
H	3.089375	13.876833	2.024387
H	3.127617	15.637948	2.171265
H	1.862329	14.852821	1.196885
C	7.984561	8.803828	8.412353
H	7.546363	8.423502	7.489406
H	7.172619	9.145252	9.063985
H	8.486272	7.963764	8.906712
C	8.624942	15.922795	7.282975
H	7.609423	16.071366	6.906463
H	9.172325	16.867829	7.168265
H	8.551448	15.693907	8.351610
C	0.421447	8.754348	0.855327
C	5.502327	5.094387	-1.987362
H	5.051550	4.612276	-1.115988
H	6.570603	5.215157	-1.782779
H	5.389903	4.416815	-2.843603
C	-3.348149	7.321167	4.117826
H	-2.849814	6.453692	3.672616
H	-3.703781	7.962820	3.304647
H	-4.224108	6.959093	4.669448
C	10.196724	5.524905	0.820634
H	9.354790	5.225691	1.450716
H	11.103931	5.479978	1.433781
H	10.289726	4.787819	0.013153
C	0.695255	7.256380	0.593531
H	0.862140	7.075767	-0.476119
H	-0.146010	6.634356	0.917601
H	1.585111	6.929547	1.135978
C	-1.258164	14.361835	9.255396
H	-2.184376	14.949116	9.250661
H	-0.424483	15.050437	9.423386
H	-1.304349	13.669498	10.103014
C	2.129730	16.629565	8.500289
H	2.018136	15.640274	8.954043
H	1.255833	16.799474	7.863955
H	2.125689	17.388714	9.294413
C	9.605960	10.345158	9.505916
H	8.829312	10.741045	10.169422

H	8.415323	15.224330	4.592578	H	10.374793	11.115975	9.394944
C	2.444297	14.606112	4.609406	H	10.067003	9.478136	9.996220
C	4.608819	7.751430	1.319038	C	-3.202312	9.259214	5.685410
C	12.614666	11.235674	1.794153	H	-3.499519	9.971665	4.908221
H	12.368003	11.199603	0.727825	H	-2.601174	9.801502	6.419307
H	13.228422	10.359739	2.031794	H	-4.109104	8.898501	6.188877
H	13.222491	12.131956	1.962610	C	4.848143	6.460895	-2.288012
C	9.860451	4.680748	6.571318	C	3.346016	6.252050	-2.582407
C	10.110683	9.384936	7.229968	H	2.860843	7.210017	-2.797746
H	10.886505	10.137067	7.053303	H	2.833303	5.809100	-1.724304
H	9.698852	9.090636	6.261959	H	3.205133	5.590006	-3.446846
H	10.585720	8.503112	7.680544	C	2.424698	11.146586	11.220594
C	-0.845123	9.183600	0.082681	H	2.095958	10.141230	10.936209
H	-1.061609	10.243292	0.257245	H	1.535276	11.720890	11.500353
H	-1.725215	8.608651	0.386522	H	3.068165	11.060883	12.106213
H	-0.703021	9.034802	-0.995405	C	5.514755	7.039395	-3.547631
C	6.187707	10.938344	-0.094789	H	6.582062	7.222697	-3.385269
C	4.628213	16.597535	8.644171	H	5.049718	7.982223	-3.853158
H	5.581062	16.588281	8.105505	H	5.414340	6.332721	-4.379792
H	4.582755	15.698725	9.262354	C	4.446045	10.986775	9.749091
H	4.627075	17.462578	9.318702	H	5.016282	11.418378	8.922734
C	3.530580	18.062804	6.968868	H	4.198709	9.953289	9.485561
H	2.689759	18.233618	6.291219	H	5.094620	10.959720	10.633058
H	4.451969	18.127548	6.379840	C	11.189410	7.256831	-0.679906
H	3.537136	18.876188	7.706313	H	12.137325	7.249124	-0.130639
C	11.792984	11.474455	4.140369	H	11.079493	8.236416	-1.156681
H	12.364477	10.606255	4.486575	H	11.254679	6.504764	-1.474683
H	10.939283	11.599998	4.810517	C	8.707670	6.970403	-0.610469
H	12.425559	12.365761	4.237633	H	8.543819	7.963637	-1.038998
C	0.656857	13.439724	3.245689	H	7.821047	6.734491	-0.017504
H	-0.075512	13.415643	4.056411	H	8.773869	6.246143	-1.432954
H	1.231721	12.515218	3.310966	C	6.278938	2.653702	1.307248
H	0.110939	13.441052	2.293546	H	6.614484	3.658070	1.035776
C	-1.080897	14.658605	6.784640	H	5.485734	2.367572	0.607382
H	-1.948371	15.329627	6.848148	H	7.124490	1.968735	1.166869
H	-1.099758	14.177368	5.804050	C	5.382965	1.141944	3.080528
H	-0.164905	15.253374	6.832744	H	4.568313	0.793309	2.436033
C	1.551459	14.691912	3.358907	H	5.066454	1.027635	4.122398
C	5.073661	11.917238	0.345866	H	6.244655	0.483952	2.919605
H	4.750710	11.712680	1.370004	C	6.914718	3.010284	3.720358
H	4.197229	11.830802	-0.306179	H	6.584516	2.976239	4.762250
H	5.433709	12.952581	0.308592	H	7.255051	4.031780	3.531347
C	9.997987	6.942700	0.239882	H	7.773003	2.335835	3.606270

Table S14. Cartesian coordinates of **2**$_{\mathbf{TR}}$.

Energy: -4744.55954807 Hartree, no imaginary frequence is found.

Atom	X	Y	Z	Atom	X	Y	Z
C	15.844116	14.522143	15.458148	C	7.301753	14.524122	20.626739
H	15.265279	15.170072	14.795369	H	7.880892	15.172651	21.288676
H	16.901007	14.774758	15.324224	H	6.244949	14.777122	20.760617
H	15.574191	14.774846	16.485118	H	7.571494	14.775670	19.599437
C	16.561166	12.182014	16.025048	C	6.584429	12.183418	20.062282
H	16.285368	12.271554	17.080232	H	6.860413	12.271697	19.007040
H	17.601101	12.507986	15.912044	H	5.544520	12.509658	20.174751
H	16.509327	11.121607	15.759903	H	6.636107	11.123311	20.328665
C	15.623161	13.032781	15.139840	C	7.522450	13.035054	20.946648
C	14.176747	12.589114	15.399757	C	8.968799	12.590894	20.687248
C	12.490741	10.807817	15.298532	C	10.654359	10.809245	20.789569
C	15.978841	12.810978	13.653004	C	7.166601	12.814967	22.433692
H	15.896554	11.759027	13.364335	H	7.248593	11.763326	22.723555
H	17.008493	13.131773	13.458560	H	6.137009	13.136226	22.627688
H	15.313060	13.391274	13.006313	H	7.832464	13.395823	23.079799
C	12.041602	9.387939	14.925507	C	11.103140	9.389469	21.163407
C	13.189319	8.537672	14.357595	C	9.955178	8.539782	21.731694
H	13.606045	8.974797	13.444177	H	9.538409	8.977554	22.644783
H	12.818729	7.538207	14.107760	H	10.325540	7.540400	21.982199
H	14.001590	8.419411	15.082401	H	9.142988	8.421225	21.006845
C	13.795060	11.269340	15.101629	C	9.350172	11.271231	20.986262
H	14.537351	10.597263	14.689635	H	8.607708	10.599583	21.398642
C	10.930823	9.484766	13.854000	C	12.213861	9.486627	22.234945
H	10.074050	10.062707	14.212464	H	13.070761	10.064236	21.876250
H	10.574409	8.485846	13.578043	H	12.570078	8.487780	22.511421
H	11.308610	9.974156	12.950102	H	11.836100	9.976533	23.138573
C	11.488401	8.678426	16.179376	C	11.656270	8.679140	19.909972
H	12.262939	8.589692	16.947122	H	10.881758	8.590112	19.142234
H	11.137982	7.671166	15.926927	H	12.006484	7.671957	20.163014
H	10.649764	9.227261	16.616659	H	12.495036	9.227566	19.472421
C	11.550633	11.704820	15.817352	C	11.594684	11.705700	20.270188
H	10.523644	11.391551	15.968908	H	12.621579	11.392066	20.118774
C	11.887714	13.028399	16.121445	C	11.257876	13.029123	19.965101
C	13.212210	13.456363	15.911412	C	9.933489	13.457526	20.174866
H	13.459122	14.486866	16.119086	H	9.686744	14.487874	19.966251
C	10.823078	13.974908	16.517727	C	12.322728	13.975162	19.568300
C	9.606479	13.966004	15.674074	C	13.539394	13.966302	20.411862
C	9.745558	13.700319	14.297961	C	13.400463	13.700561	21.787980
H	10.741678	13.548892	13.906886	H	12.404385	13.549168	22.179184
C	8.641548	13.628267	13.451666	C	14.504579	13.628472	22.634140

C	7.373362	13.837108	14.010790	C	15.772703	13.837307	22.074864
H	6.503196	13.767703	13.366913	H	16.642956	13.767750	22.718610
C	7.184137	14.116280	15.370266	C	15.961776	14.116413	20.715360
C	8.315299	14.171984	16.193661	C	14.830519	14.172141	19.892105
H	8.208322	14.345022	17.254834	H	14.937382	14.344937	18.830883
C	8.772688	13.306307	11.955952	C	14.373689	13.306068	24.129778
C	10.235867	13.091254	11.535029	C	12.910572	13.091061	24.550938
H	10.838301	13.988890	11.701805	H	12.308225	13.988832	24.384570
H	10.282009	12.852484	10.467603	H	12.864629	12.851972	25.618302
H	10.698505	12.265415	12.084632	H	12.447692	12.265448	24.001200
C	7.989270	12.010774	11.648369	C	15.157042	12.010354	24.436768
H	8.387220	11.172186	12.229376	H	14.758888	11.171989	23.855580
H	8.069559	11.758620	10.584894	H	15.076975	11.757861	25.500181
H	6.926999	12.111312	11.889349	H	16.219264	12.110888	24.195568
C	8.194124	14.470679	11.123784	C	14.952540	14.470099	24.962225
H	7.141128	14.651634	11.357797	H	16.005521	14.651005	24.728109
H	8.268576	14.249622	10.053002	H	14.878245	14.248695	26.032946
H	8.741874	15.397652	11.321198	H	14.404871	15.397201	24.765202
C	5.752950	14.255389	15.918926	C	17.392875	14.255901	20.166575
C	5.101649	12.853567	15.914225	C	18.044439	12.854205	20.170847
H	5.048554	12.442087	14.901067	H	18.097714	12.442479	21.183896
H	4.083070	12.901119	16.316467	H	19.062966	12.902043	19.768507
H	5.680265	12.155957	16.528825	H	17.465884	12.156641	19.556135
C	4.909876	15.211511	15.048898	C	18.235847	15.211977	21.036761
H	5.309336	16.227247	15.091566	H	17.836173	16.227646	20.994393
H	3.881263	15.240320	15.426122	H	19.264413	15.241099	20.659433
H	4.863950	14.899901	14.001470	H	18.281951	14.900112	22.084106
C	5.736369	14.804824	17.351980	C	17.409180	14.805737	18.733665
H	6.196541	14.109339	18.053146	H	16.949040	14.110369	18.032346
H	4.703448	14.959310	17.679739	H	18.442034	14.960497	18.405816
H	6.256793	15.762760	17.420401	H	16.888574	15.763599	18.665583
C	10.980054	14.845071	17.579206	C	12.165796	14.845113	18.506641
C	10.123567	15.900921	17.865100	C	13.022417	15.900774	18.220504
C	9.364424	16.794050	18.257628	C	13.781658	16.793948	17.828290
C	8.487077	17.737388	18.715741	C	14.658767	17.737487	17.370134
C	7.426719	18.176385	17.796658	C	15.719129	18.176548	18.289167
C	7.699511	18.263362	16.420742	C	15.446358	18.263526	19.665088
H	8.706499	18.038314	16.087610	H	14.439342	18.038568	19.998244
C	6.719343	18.641820	15.503235	C	16.426577	18.641914	20.582575
C	5.431287	18.915846	15.989351	C	17.714650	18.915825	20.096431
H	4.660515	19.208036	15.291093	H	18.485441	19.208028	20.794662
C	5.110578	18.808787	17.350816	C	18.035272	18.808938	18.734933
C	6.121656	18.445200	18.242816	C	17.024155	18.445410	17.842960
H	5.899377	18.319768	19.295770	H	17.246345	18.320198	16.789960

C	7.090156	18.762395	14.016359	C	16.055706	18.762926	22.069396
C	7.620142	17.407308	13.500087	C	15.525153	17.408203	22.586003
H	8.437300	17.022320	14.112821	H	14.707825	17.023340	21.973415
H	7.988204	17.512089	12.473652	H	15.157127	17.513431	23.612405
H	6.832609	16.651515	13.500471	H	16.312370	16.652085	22.585847
C	5.890432	19.170363	13.145094	C	17.255537	19.170630	22.940644
H	5.079500	18.437326	13.208345	H	18.066303	18.437410	22.877343
H	6.201085	19.230655	12.097090	H	16.944917	19.230992	23.988653
H	5.492849	20.149910	13.430637	H	17.653317	20.150098	22.655111
C	8.191228	19.835387	13.862464	C	14.955060	19.836423	22.222877
H	7.841160	20.808391	14.223059	H	15.305612	20.809179	21.862085
H	8.474439	19.940927	12.808998	H	14.671736	19.942363	23.276273
H	9.090327	19.574219	14.427461	H	14.055963	19.575490	21.657767
C	2.734770	19.569168	16.800151	C	20.411382	19.568364	19.285645
H	3.091168	20.519909	16.390045	H	20.055320	20.519131	19.695984
H	1.744156	19.741034	17.233198	H	21.402026	19.739995	18.852574
H	2.614272	18.862505	15.972879	H	20.531702	18.861477	20.112752
C	3.708340	20.032766	19.051746	C	19.437861	20.032767	17.034195
H	4.113231	20.997697	18.729500	H	19.033321	20.997771	17.356662
H	4.319766	19.673094	19.884185	H	18.826271	19.673499	16.201699
H	2.694477	20.197053	19.432979	H	20.451768	20.196777	16.652961
C	3.133662	17.671769	18.379953	C	20.011702	17.671410	17.705474
H	3.110894	16.942132	17.564166	H	20.034137	16.941562	18.521085
H	2.114695	17.788613	18.766505	H	21.030733	17.787926	17.318991
H	3.755265	17.258449	19.179743	H	19.389962	17.258541	16.905556
C	3.685019	19.026544	17.880651	C	19.460882	19.026293	18.205084
C	8.019730	19.421715	20.523175	C	15.125553	19.422316	15.563060
H	7.435974	19.991744	19.814448	H	15.709573	19.992111	16.271764
C	8.182176	19.878774	21.827890	C	14.962501	19.879892	14.258607
C	8.983633	19.126523	22.707847	C	14.160755	19.127920	13.378676
H	9.105133	19.475957	23.725483	H	14.038780	19.477763	12.361238
C	9.632359	17.953727	22.304482	C	13.512492	17.954780	13.781767
C	9.448538	17.524762	20.989808	C	13.696829	17.525342	15.096216
H	9.939715	16.624240	20.643147	H	13.206039	16.624526	15.442655
C	8.635739	18.229538	20.083554	C	14.509714	18.229980	16.002498
C	7.518463	21.169290	22.331173	C	15.625800	21.170708	13.755568
C	6.727943	21.890342	21.226086	C	16.417370	21.890987	14.860396
H	7.371495	22.180191	20.389226	H	15.774605	22.180526	15.697968
H	6.278258	22.802234	21.631978	H	16.866904	22.803019	14.454646
H	5.917367	21.268768	20.833641	H	17.228144	21.269015	15.251833
C	6.539336	20.818856	23.473363	C	16.603846	20.821071	12.612207
H	5.761087	20.135377	23.117948	H	17.382360	20.137242	12.966357
H	6.053025	21.724675	23.852915	H	17.089867	21.727155	12.232924
H	7.051408	20.336277	24.311029	H	16.090959	20.339217	11.774626

C	8.601965	22.136066	22.857927	C	14.541784	22.137809	13.230458
H	9.313100	22.389167	22.064948	H	13.831417	22.390437	14.024277
H	8.143030	23.064431	23.216109	H	15.000353	23.066386	12.872355
H	9.166325	21.700914	23.687617	H	13.976630	21.703088	12.401082
C	10.517534	17.118410	23.241918	C	12.627294	17.119589	12.844251
C	9.881162	15.722445	23.427244	C	13.264556	15.724233	12.657424
H	8.856202	15.807981	23.803907	H	14.289268	15.810798	12.280326
H	10.457699	15.132419	24.147862	H	12.688075	15.134402	11.936598
H	9.852633	15.161323	22.489576	H	13.293952	15.162363	13.594614
C	10.677803	17.767903	24.626130	C	12.465657	17.770069	11.460661
H	11.127963	18.763691	24.554871	H	12.014801	18.765463	11.533016
H	11.333679	17.149714	25.247639	H	11.809838	17.151890	10.839082
H	9.718778	17.860374	25.146818	H	13.424270	17.863686	10.939418
C	11.920320	16.961429	22.617535	C	11.225028	16.961120	13.469404
H	11.886490	16.468331	21.642342	H	11.259801	16.467406	14.444248
H	12.563205	16.355604	23.264119	H	10.582192	16.355233	12.822833
H	12.395088	17.938656	22.486674	H	10.749603	17.937902	13.601214

Table S15. Cartesian coordinates of $\mathbf{2}_{\mathbf{TR}}^{-}$.

Energy: -4744.60915422 Hartree, no imaginary frequence is found.

Atom	X	Y	Z	Atom	X	Y	Z
C	15.828123	14.482416	15.477201	H	7.787548	13.274135	23.034075
H	15.278017	15.118951	14.780116	H	12.289198	17.984359	22.568816
H	16.893424	14.708772	15.360384	C	7.316408	14.480036	20.609528
H	15.539019	14.780898	16.485904	H	7.866501	15.116780	21.306427
C	16.468049	12.147403	16.153730	H	6.251053	14.705908	20.726773
H	16.162003	12.292950	17.194218	H	7.604973	14.778690	19.600722
H	17.521901	12.434722	16.060579	C	6.677377	12.144801	19.932966
H	16.386067	11.078468	15.931621	H	6.983104	12.290581	18.892421
C	15.583517	12.987901	15.205380	H	5.623419	12.431630	20.026403
C	14.120283	12.582364	15.433010	H	6.759901	11.075883	20.154957
C	12.394779	10.846373	15.316238	C	7.561764	12.985612	20.881175
C	15.989536	12.707164	13.741883	C	9.025123	12.580704	20.653172
H	15.899874	11.647386	13.484273	C	10.751326	10.845356	20.769364
H	17.031417	13.005006	13.575130	C	7.156181	12.704568	22.344736
H	15.358035	13.276488	13.052463	H	7.246403	11.644813	22.602239
C	11.910703	9.439208	14.936459	C	11.236138	9.438491	21.149311
C	13.035251	8.564260	14.359159	C	10.112544	8.563720	21.728735
H	13.461956	9.000499	13.449875	H	9.687223	9.000328	22.638489
H	12.639619	7.575629	14.101602	H	10.508636	7.575226	21.986110
H	13.845333	8.421786	15.082061	H	9.301343	8.420889	21.007157
C	13.712275	11.272581	15.127451	C	9.433725	11.271064	20.958560
H	14.442349	10.582342	14.723051	H	8.704109	10.580628	21.363447

C	10.798740	9.565733	13.869697	C	12.349624	9.565898	22.214374
H	9.960566	10.166905	14.232732	H	13.187169	10.166964	21.849687
H	10.414222	8.576814	13.592127	H	12.734708	8.577228	22.492048
H	11.185850	10.048243	12.966049	H	11.963762	10.048967	23.118262
C	11.346552	8.733944	16.187359	C	11.798590	8.732484	19.898062
H	12.126743	8.613556	16.944791	H	11.017350	8.611573	19.141802
H	10.960089	7.740317	15.929733	H	12.185465	7.739040	20.155774
H	10.533096	9.307534	16.639684	H	12.611384	9.305822	19.444240
C	11.475887	11.763278	15.837133	C	11.669881	11.762841	20.248910
H	10.441770	11.472216	15.986748	H	12.704185	11.472330	20.099495
C	11.836570	13.082124	16.140405	C	11.308675	13.081644	19.946072
C	13.172629	13.474041	15.935271	C	9.972410	13.472912	20.151141
H	13.446065	14.496310	16.150743	H	9.698626	14.495216	19.936229
C	10.787515	14.047632	16.541480	C	12.357337	14.047678	19.545227
C	9.563242	14.039933	15.701226	C	13.581797	14.040115	20.385206
C	9.691287	13.760129	14.326283	C	13.454122	13.760682	21.760261
H	10.686036	13.608510	13.931264	H	12.459509	13.608814	22.155527
C	8.583094	13.664496	13.486763	C	14.562605	13.664572	22.599347
C	7.315224	13.871402	14.045534	C	15.830307	13.871526	22.040206
H	6.441278	13.788010	13.407551	H	16.704408	13.788759	22.678063
C	7.136560	14.174639	15.401355	C	16.008503	14.175788	20.684550
C	8.271309	14.251355	16.218149	C	14.873522	14.252295	19.868053
H	8.168803	14.459498	17.272917	H	14.975648	14.461297	18.813410
C	8.709708	13.321753	11.994532	C	14.436280	13.323646	24.092019
C	10.172204	13.119054	11.565365	C	12.973723	13.123335	24.522092
H	10.761759	14.028051	11.712641	H	12.385300	14.032954	24.374123
H	10.213918	12.861487	10.501369	H	12.932171	12.866954	25.586382
H	10.651431	12.311724	12.127592	H	12.493168	12.316044	23.960940
C	7.939940	12.013249	11.708668	C	15.204412	12.014424	24.378986
H	8.352353	11.187592	12.298061	H	14.790665	11.188686	23.790638
H	8.014224	11.747021	10.647316	H	15.130170	11.749435	25.440651
H	6.878975	12.105483	11.959048	H	16.265405	12.104958	24.128118
C	8.112654	14.466391	11.148194	C	15.035280	14.468440	24.936776
H	7.058820	14.637144	11.386338	H	16.089176	14.637641	24.697774
H	8.185245	14.232951	10.079050	H	14.963081	14.236220	26.006212
H	8.649225	15.402090	11.333247	H	14.499742	15.404607	24.751076
C	5.706922	14.328750	15.951578	C	17.437987	14.329849	20.133888
C	5.033807	12.937286	15.936166	C	18.110773	12.938210	20.148254
H	4.975397	12.533196	14.920013	H	18.169432	12.533499	21.164146
H	4.015186	12.997000	16.338597	H	19.129272	12.997915	19.745513
H	5.601874	12.227203	16.546311	H	17.542328	12.228625	19.537876
C	4.879574	15.303298	15.086463	C	18.265882	15.303688	20.999273
H	5.290470	16.313730	15.143304	H	17.855202	16.314251	20.943176
H	3.847952	15.340465	15.456721	H	19.297379	15.340824	20.628664

H	4.839984	15.002209	14.035301	H	18.305772	15.001970	22.050244
C	5.694501	14.867626	17.388522	C	17.450054	14.869555	18.697249
H	6.145330	14.161141	18.084441	H	16.998653	14.163650	18.001106
H	4.662358	15.034038	17.714711	H	18.482141	15.035713	18.370748
H	6.227502	15.817449	17.465864	H	16.917429	15.819664	18.620661
C	10.962544	14.920994	17.595962	C	12.181994	14.921128	18.490888
C	10.092515	15.961731	17.914647	C	13.051708	15.962176	18.172345
C	9.313519	16.829636	18.319745	C	13.830934	16.829927	17.767364
C	8.409263	17.754329	18.789243	C	14.734998	17.754846	17.297953
C	7.372942	18.208943	17.851884	C	15.772147	18.208519	18.234857
C	7.676228	18.302190	16.480053	C	15.469580	18.302096	19.606838
H	8.689181	18.066026	16.173445	H	14.456585	18.066718	19.913948
C	6.725772	18.685050	15.535071	C	16.420726	18.684538	20.551309
C	5.424922	18.973933	15.978569	C	17.721574	18.972435	20.107154
H	4.676833	19.272919	15.258647	H	18.470125	19.271331	20.826636
C	5.072119	18.866548	17.331687	C	18.073632	18.864747	18.753865
C	6.053351	18.489428	18.251894	C	17.091721	18.488125	17.834185
H	5.794876	18.359558	19.296447	H	17.349582	18.358084	16.789501
C	7.142985	18.793882	14.058886	C	16.004071	18.794870	22.027546
C	7.678560	17.433074	13.564489	C	15.468565	17.434620	22.523525
H	8.486849	17.053829	14.192433	H	14.660046	17.054768	21.896248
H	8.061354	17.526278	12.541683	H	15.086124	17.528906	23.546362
H	6.891216	16.677006	13.563267	H	16.255864	16.678515	22.525275
C	5.973470	19.204573	13.148235	C	17.173963	19.206356	22.937352
H	5.155565	18.477784	13.193712	H	17.991862	18.479539	22.892157
H	6.315894	19.255399	12.109002	H	16.831971	19.258062	23.976685
H	5.574067	20.188598	13.416528	H	17.573233	20.190162	22.668060
C	8.255998	19.857159	13.928408	C	14.891190	19.858363	22.157381
H	7.902778	20.835070	14.273498	H	15.244329	20.835895	21.811129
H	8.571731	19.953607	12.882667	H	14.575894	19.955917	23.203153
H	9.134888	19.591812	14.521742	H	14.012030	19.592448	21.564690
C	2.714784	19.645720	16.721015	C	20.431967	19.641742	19.363453
H	3.089672	20.596437	16.327336	H	20.058045	20.592648	19.757599
H	1.712491	19.820340	17.127410	H	21.434161	19.815693	18.956525
H	2.615520	18.946144	15.884955	H	20.531175	18.941865	20.199267
C	3.625208	20.085213	19.001897	C	19.520754	20.082433	17.083118
H	4.054972	21.046877	18.702737	H	19.091931	21.044403	17.382653
H	4.205052	19.710072	19.849304	H	18.940197	19.707926	16.235918
H	2.600556	20.259662	19.351202	H	20.545385	20.256096	16.733360
C	3.056452	17.735311	18.295506	C	20.087784	17.731898	17.788810
H	3.049923	17.013859	17.472175	H	20.094200	17.010330	18.612043
H	2.028342	17.855018	18.659212	H	21.115771	17.850767	17.424483
H	3.657854	17.310076	19.103926	H	19.485513	17.307288	16.980707
C	3.632435	19.089112	17.822550	C	19.513256	19.086133	18.262297

C	7.868129	19.354605	20.665224	C	15.275725	19.355496	15.422110
H	7.265647	19.932320	19.978377	H	15.878704	19.932885	16.108793
C	8.009101	19.766572	21.987903	C	15.134765	19.767428	14.099414
C	8.836294	19.019755	22.847655	C	14.307236	19.020832	13.239786
H	8.939854	19.334234	23.878847	H	14.203773	19.335203	12.208551
C	9.530700	17.891838	22.394878	C	13.612218	17.893388	13.692806
C	9.365094	17.503844	21.065315	C	13.777976	17.505335	15.022320
H	9.898220	16.640422	20.686797	H	13.244888	16.641915	15.400848
C	8.527837	18.203946	20.173888	C	14.615949	18.204889	15.913483
C	7.291635	21.010160	22.537202	C	15.853259	21.010269	13.549764
C	6.469220	21.739913	21.462008	C	16.676707	21.739343	14.624630
H	7.099850	22.089275	20.638376	H	16.046681	22.089294	15.448477
H	5.977939	22.614447	21.902974	H	17.168599	22.613424	14.183446
H	5.690130	21.097616	21.041371	H	17.455391	21.096380	15.045006
C	6.330598	20.579525	23.667325	C	16.813527	20.578582	12.419386
H	5.582877	19.875049	23.287749	H	17.560744	19.873501	12.798834
H	5.805183	21.448502	24.082347	H	17.339584	21.447013	12.004032
H	6.867490	20.087323	24.483470	H	16.275915	20.086711	11.603513
C	8.332675	22.003605	23.098881	C	14.813020	22.004678	12.988312
H	9.029799	22.313830	22.313873	H	14.116412	22.315624	13.773493
H	7.836735	22.899190	23.492722	H	15.309718	22.899760	12.594285
H	8.919158	21.560181	23.908473	H	14.225888	21.561784	12.178904
C	10.451821	17.058311	23.300009	C	12.691624	17.059400	12.787562
C	9.874904	15.631039	23.432887	C	13.267526	15.631440	12.657566
H	8.848082	15.662473	23.813746	H	14.294905	15.661461	12.278079
H	10.477172	15.035761	24.128360	H	12.665723	15.035633	11.962146
H	9.865691	15.107765	22.473802	H	13.275001	15.109582	13.617452
C	10.585269	17.658430	24.709605	C	12.560812	17.657618	11.376916
H	10.993557	18.674098	24.676842	H	12.153697	18.673820	11.407645
H	11.265229	17.043113	25.308752	H	11.880927	17.042239	10.777748
H	9.621493	17.692809	25.229219	H	13.525327	17.690077	10.858549
C	11.860725	16.982704	22.673424	C	11.281679	16.985989	13.412104
H	11.847299	16.517138	21.684569	H	11.293116	16.521783	14.401616
H	12.532456	16.389813	23.303479	H	10.610345	16.392883	12.781836
H	6.114191	13.001897	22.511727	H	10.854005	17.988190	13.514664

Table S16. Cartesian coordinates of $\mathbf{2}_{\mathbf{TR}}^{2-}$.

Energy: -4744.59139204 Hartree, no imaginary frequence is found.

Atom	X	Y	Z	Atom	X	Y	Z
C	15.827707	14.508506	15.483034	C	7.317652	14.508924	20.602870
H	15.288799	15.144236	14.776852	H	7.855713	15.144409	21.309912
H	16.896330	14.724534	15.373151	H	6.248895	14.724910	20.711528
H	15.531439	14.822852	16.484303	H	7.615161	14.823607	19.602078

C	16.443409	12.172486	16.181955	C	6.702338	12.173131	19.902884
H	16.136451	12.335143	17.219423	H	7.010055	12.336075	18.865685
H	17.501847	12.444656	16.088726	H	5.643836	12.445292	19.995412
H	16.345954	11.101924	15.971601	H	6.799635	11.102512	20.113020
C	15.571481	13.014188	15.222973	C	7.573586	13.014534	20.862754
C	14.103278	12.621430	15.444671	C	9.041926	12.621766	20.642021
C	12.363838	10.905021	15.319801	C	10.781286	10.905350	20.767742
C	15.984786	12.719036	13.764085	C	7.159190	12.719044	22.321260
H	15.891184	11.657441	13.513344	H	7.252593	11.657389	22.571835
H	17.029305	13.011623	13.600697	H	6.114546	13.011586	22.483917
H	15.359276	13.286903	13.068078	H	7.784161	13.286751	23.017887
C	11.866293	9.501000	14.943245	C	11.278583	9.501259	21.144375
C	12.980756	8.614984	14.362728	C	10.163577	8.614925	21.723364
H	13.408921	9.048985	13.452965	H	9.734340	9.048590	22.632783
H	12.575938	7.628849	14.106556	H	10.568226	7.628757	21.979680
H	13.791958	8.467761	15.083478	H	9.353206	8.467834	21.001657
C	13.686706	11.315633	15.132235	C	9.458313	11.315951	20.954612
H	14.412196	10.619173	14.729394	H	8.732518	10.619353	21.356667
C	10.751156	9.632431	13.880973	C	12.392502	9.632414	22.207963
H	9.924126	10.248253	14.244112	H	13.219919	10.248390	21.845984
H	10.351805	8.646338	13.610822	H	12.791589	8.646254	22.478257
H	11.140327	10.104980	12.972939	H	12.002264	10.104651	23.115699
C	11.301931	8.800893	16.196741	C	11.844434	8.801677	19.891258
H	12.086533	8.672394	16.948175	H	11.060679	8.673313	19.138914
H	10.901115	7.811016	15.942731	H	12.245137	7.811779	20.145362
H	10.500535	9.386036	16.655167	H	12.646235	9.387124	19.433922
C	11.453654	11.829961	15.840044	C	11.691560	11.830034	20.247193
H	10.416920	11.547070	15.989194	H	12.728275	11.547009	20.098163
C	11.819780	13.149857	16.143618	C	11.325569	13.149892	19.943290
C	13.163717	13.522130	15.946438	C	9.981651	13.522308	20.140291
H	13.450242	14.538696	16.172290	H	9.695086	14.538669	19.913601
C	10.773897	14.116987	16.548882	C	12.371503	14.116891	19.537846
C	9.541537	14.092386	15.720438	C	13.604035	14.092158	20.366006
C	9.655947	13.791488	14.346205	C	13.489951	13.791500	21.740318
H	10.649268	13.647053	13.944685	H	12.496721	13.647270	22.142134
C	8.543076	13.663866	13.517220	C	14.603039	13.664010	22.569038
C	7.275073	13.863702	14.077897	C	15.870903	13.863652	22.007977
H	6.396622	13.758814	13.448506	H	16.749521	13.758782	22.637142
C	7.107999	14.199257	15.428424	C	16.037639	14.198945	20.657347
C	8.246553	14.308508	16.235094	C	14.898873	14.308236	19.850988
H	8.149140	14.556545	17.281300	H	14.995989	14.556291	18.804754
C	8.664469	13.298146	12.029241	C	14.482046	13.298563	24.057108
C	10.127086	13.131362	11.585325	C	13.019564	13.131381	24.501312
H	10.689113	14.060576	11.711047	H	12.457177	14.060346	24.375367

H	10.164559	12.855815	10.524850	H	12.982360	12.856172	25.561884
H	10.638166	12.350863	12.156778	H	12.508687	12.350501	23.930197
C	7.928870	11.964044	11.773791	C	15.218148	11.964763	24.312690
H	8.373623	11.160732	12.370260	H	14.773565	11.161181	23.716461
H	7.994215	11.681875	10.714992	H	15.153087	11.682786	25.371558
H	6.870167	12.032089	12.041477	H	16.276781	12.033115	24.044802
C	8.025876	14.409014	11.168139	C	15.120424	14.409837	24.917847
H	6.969563	14.551983	11.413702	H	16.176649	14.553091	24.672071
H	8.096786	14.160668	10.101310	H	15.049770	14.161719	25.984745
H	8.534635	15.363126	11.335171	H	14.611326	15.363747	24.750676
C	5.679472	14.361103	15.981696	C	17.466018	14.360787	20.103720
C	5.001723	12.971614	15.977536	C	18.143915	12.971368	20.108042
H	4.941097	12.559546	14.964355	H	18.204879	12.559558	21.121305
H	3.983257	13.036753	16.381513	H	19.162254	13.036511	19.703748
H	5.569480	12.265124	16.592266	H	17.576050	12.264665	19.493657
C	4.852789	15.331501	15.110668	C	18.292774	15.331475	20.974348
H	5.259687	16.343476	15.169978	H	17.885827	16.343417	20.914784
H	3.818314	15.365976	15.475402	H	19.327206	15.365876	20.609487
H	4.821856	15.028519	14.059311	H	18.323833	15.028811	22.025796
C	5.668349	14.911238	17.413974	C	17.476755	14.910553	18.671296
H	6.125371	14.212352	18.113134	H	17.019589	14.211458	17.972449
H	4.635565	15.075843	17.741359	H	18.509450	15.075095	18.343606
H	6.197298	15.863497	17.483370	H	16.947742	15.862772	18.601756
C	10.952392	14.998703	17.602574	C	12.192957	14.998673	18.484225
C	10.061425	16.012511	17.941884	C	13.083844	16.012612	18.145031
C	9.263396	16.856639	18.358863	C	13.882502	16.855935	17.727584
C	8.327399	17.757555	18.836312	C	14.818698	17.756534	17.249950
C	7.312140	18.214082	17.887320	C	15.833731	18.213432	18.199022
C	7.631499	18.286675	16.511726	C	15.514270	18.285758	19.574599
H	8.644545	18.026476	16.225087	H	14.501429	18.024715	19.861201
C	6.708801	18.674765	15.545248	C	16.436761	18.674112	20.541166
C	5.401474	18.996633	15.952265	C	17.743838	18.997037	20.134188
H	4.673765	19.301035	15.213810	H	18.471267	19.302055	20.872665
C	5.028692	18.911065	17.301955	C	18.116597	18.912285	18.784440
C	5.983890	18.529943	18.247733	C	17.161694	18.530507	17.838618
H	5.696281	18.414747	19.286652	H	17.449250	18.416176	16.799584
C	7.164962	18.766192	14.078231	C	15.980361	18.765629	22.008100
C	7.700413	17.398778	13.603104	C	15.445921	17.397940	22.483573
H	8.491647	17.015421	14.250031	H	14.655250	17.013652	21.836521
H	8.106377	17.483361	12.587789	H	15.039517	17.482583	23.498708
H	6.907974	16.648170	13.589700	H	16.238999	16.648020	22.497562
C	6.024217	19.182241	13.133432	C	17.120684	19.182845	22.952897
H	5.196631	18.465495	13.165756	H	17.948892	18.466813	22.920759
H	6.393899	19.219929	12.102251	H	16.750900	19.220407	23.984045

H	5.629004	20.172922	13.384197	H	17.515047	20.173826	22.701977
C	8.290941	19.817893	13.967042	C	14.853553	19.816492	22.118843
H	7.938011	20.800882	14.299072	H	15.205804	20.799686	21.786701
H	8.635013	19.905637	12.928716	H	14.509184	19.904175	23.157077
H	9.150393	19.546546	14.584858	H	13.994443	19.544373	21.500883
C	2.691360	19.715257	16.638586	C	20.453247	19.718338	19.447933
H	3.086555	20.659548	16.248956	H	20.057240	20.662221	19.837727
H	1.681292	19.902148	17.022305	H	21.463150	19.906170	19.064239
H	2.605115	19.013619	15.802835	H	20.540103	19.016629	20.283562
C	3.555196	20.152959	18.936123	C	19.589056	20.155664	17.150461
H	4.010889	21.105734	18.646709	H	19.132453	21.107973	17.439978
H	4.107923	19.770440	19.797409	H	19.036759	19.772735	16.289087
H	2.523993	20.346115	19.258765	H	20.620104	20.349829	16.827930
C	2.977233	17.808294	18.225778	C	20.169009	17.811396	17.860445
H	2.981954	17.084772	17.404116	H	20.165017	17.087784	18.682026
H	1.941972	17.938568	18.568629	H	21.204120	17.942636	17.517509
H	3.559948	17.379576	19.045589	H	19.586604	17.382238	17.040645
C	3.580426	19.153000	17.760916	C	19.564662	19.155510	18.325511
C	7.727406	19.285329	20.768170	C	15.418255	19.284795	15.318362
H	7.095723	19.860883	20.105994	H	16.050207	19.860067	15.980535
C	7.868101	19.667199	22.100256	C	15.277296	19.667017	13.986399
C	8.733791	18.939520	22.938251	C	14.411446	18.939551	13.148391
H	8.835053	19.230979	23.976703	H	14.310154	19.231138	12.109977
C	9.469602	17.856088	22.439567	C	13.675736	17.855985	13.646924
C	9.306158	17.496454	21.103480	C	13.839434	17.495995	14.982895
H	9.877199	16.670476	20.697580	H	13.268540	16.669847	15.388670
C	8.428697	18.176792	20.227119	C	14.717176	18.176026	15.859228
C	7.111695	20.871354	22.687627	C	16.034060	20.870928	13.398968
C	6.232947	21.585883	21.647791	C	16.913489	21.584920	14.438590
H	6.828479	21.969796	20.813921	H	16.318427	21.968953	15.272742
H	5.717873	22.433991	22.115024	H	17.428749	22.432884	13.971300
H	5.472031	20.917718	21.234977	H	17.674274	20.916376	14.851004
C	6.197305	20.387081	23.834548	C	16.947813	20.386414	12.251639
H	5.471058	19.656970	23.461475	H	17.673900	19.655959	12.624347
H	5.646015	21.227371	24.277334	H	17.499282	21.226525	11.808736
H	6.775783	19.903990	24.627256	H	16.368825	19.903651	11.459101
C	8.123964	21.898991	23.240709	C	15.022070	21.899119	12.846406
H	8.783966	22.251340	22.441481	H	14.362476	22.251607	13.645909
H	7.603585	22.766817	23.667347	H	15.542701	22.766797	12.419774
H	8.753529	21.462865	24.021255	H	14.392070	21.463426	12.065973
C	10.431320	17.032867	23.313484	C	12.714063	17.032869	12.772849
C	9.892485	15.590059	23.436448	C	13.253145	15.590199	12.649402
H	8.871627	15.593131	23.835276	H	14.273975	15.593594	12.250502
H	10.521194	14.997281	24.111437	H	12.624497	14.997509	11.974274

H	9.877361	15.083244	22.469034	H	13.268433	15.083097	13.616659
C	10.575591	17.613850	24.730650	C	12.569580	17.614259	11.355871
H	10.949030	18.643164	24.704800	H	12.196026	18.643523	11.382078
H	11.287350	17.011043	25.306308	H	11.857828	17.011542	10.780107
H	9.621109	17.609981	25.268915	H	13.524000	17.610664	10.817494
C	11.831992	16.997767	22.665256	C	11.313439	16.997323	13.421136
H	11.815439	16.541491	21.672486	H	11.330096	16.540654	14.413712
H	12.528229	16.416184	23.279985	H	10.617250	16.415893	12.806215
H	12.234314	18.010500	22.562125	H	10.910981	18.009956	13.524693

IX. References

[1] N. V. Kozhemyakina, J. Nuss, M. Jansen, Demonstration of the “Break-and-Seal” Approach to Fullerides of Complex Cations at the Example of $KC_{60}(THF)_5 \cdot 2THF$, *Z. Anorg. Allg. Chem.* **2009**, *635*, 1355-1361.

[2] Z. Zhou, M. A. Johnson, Z. Wei, M. U. Bühringer, M. H. Garner, R. Tykwinski, M. A. Petrukhina, Bending a Cumulene with Electrons: Stepwise Chemical Reduction and Structural Study of a Tetraaryl[4]Cumulene, *Chem. Eur. J.* **2024**, *30*, e202304145.

[3] SAINT; part of Bruker APEX3 software package (version 2021.9-4): Bruker AXS, **2021**.

[4] SADABS; part of Bruker APEX3 software package (version 2021.9-4): Bruker AXS, **2021**.

[5] G. M. Sheldrick, SHELXT - Integrated Space-Group and Crystal-Structure Determination, *Acta Crystallogr.* **2015**, *A71*, 3-8.

[6] G. M. Sheldrick, Crystal Structure Refinement with SHELXL, *Acta Crystallogr.* **2015**, *C71*, 3-8.

[7] O. V. Dolomanov, L. J. Bourhis, R. J. Gildea, J. A. K. Howard, H. Puschmann, OLEX2: A Complete Structure Solution, Refinement and Analysis Program, *J. Appl. Crystallogr.* **2009**, *42*, 339-341.

[8] C. Lee, W. Yang, R. G. Parr, Development of the Colle-Salvetti correlation-energy formula into a functional of the electron density, *Phys. Rev. B*, **1988**, *37*, 785-789.

[9] A. D. Becke, Density-functional thermochemistry. III. The role of exact exchange, *J. Chem. Phys.* **1993**, *98*, 5648-5652.

[10] F. Weigend, R. Ahlrichs, Balanced Basis Sets of Split Valence, Triple Zeta Valence and Quadruple Zeta Valence Quality for H to Rn: Design and Assessment of Accuracy, *Phys. Chem. Chem. Phys.* **2005**, *7*, 3297-3305.

[11] S. Grimme, J. Antony, S. Ehrlich, H. Krieg, A Consistent and Accurate *ab* Initio Parametrization of Density Functional Dispersion Correction (DFT-D) for the 94 Elements H-Pu, *J. Chem. Phys.* **2010**, *132*, 154104.

[12] S. Grimme, S. Ehrlich, L. Goerigk, Effect of the Damping Function in Dispersion Corrected Density Functional Theory, *J. Comput. Chem.* **2011**, *32*, 1456-1465.

[13] T. Lu, Q. Chen, Interaction Region Indicator: A Simple Real Space Function Clearly Revealing Both Chemical Bonds and Weak Interactions**, *Chemistry–Methods*, **2021**, *1*, 231-239.

[14] T. Lu, F. Chen, Multiwfn: A Multifunctional Wavefunction Analyzer*, J. Comput. Chem.* **2012**, *33*, 580-592.
